# PROTOCOL: Impact of social protection on gender equality in low‐ and middle‐income countries: A systematic review of reviews

**DOI:** 10.1002/cl2.1161

**Published:** 2021-05-04

**Authors:** Camila Perera, Shivit Bakrania, Alessandra Ipince, Zahrah Nesbitt‐Ahmed, Oluwaseun Obasola, Dominic Richardson

**Affiliations:** ^1^ UNICEF Office of Research—Innocenti Florence Italy

## Abstract

This is the protocol for a Campbell review. The review aims to systematically collect, appraise, map and synthesise the evidence from systematic reviews on the differential gender impacts of social protection programmes in Low‐ and Middle‐Income Countries (LMICs). Therefore, it will answer the following questions: (1) What is known from systematic reviews on the gender‐differentiated impacts of social protection programmes in LMICs? (2) What is known from systematic reviews about the factors that determine these gender‐differentiated impacts? (3) What is known from existing systematic reviews about design and implementation features of social protection programmes and their association with gender outcomes?

## 1 BACKGROUND

Gender and age determine how people experience opportunities, vulnerabilities and risks. In low‐income settings, adult women tend to have fewer economic resources to cope with crises such as sickness or death of family members, extreme weather events or emergencies (Wenham et al., 2020) and adult men are also affected by restrictive gender norms which translate into negative social and health outcomes for all (Heise et al., 2019). In conflict‐affected settings, adolescent girls are at higher risk of child marriage, which further hinders school enrolment and attendance, while adolescent boys are more likely to engage or be forced into child labour (Jones, 2019). In emergencies, older adults and children are more vulnerable to protection harms and health risks such as poor nutrition and violence (Karunakara & Stevenson, 2012; Stark & Landis, 2016).

Social protection programmes, such as cash transfers, pensions or unemployment benefits, aim to tackle poverty and adversity, manage risks and improve quality of life from childhood through to old age. Increased socioeconomic insecurity, inadequate resources and limited access to services mean that demand for social protection is higher in low and middle‐income settings. Various systematic reviews point to positive effects of social protection programmes on food security (Bastagli et al., 2016), school enrolment and attendance (Baird et al., 2014), sexual and reproductive health (Owusu‐Addo et al., 2018), poverty reduction (Owusu‐Addo & Cross, 2014), access to health (Erlangga et al., 2019; Habib et al., 2016), employment (Chinen et al., 2017; Kluve, 2010) and child development (Leroy et al., 2012) in Low‐ and Middle‐Income Countries (LMICs). Gender differences on the effectiveness of social protection programmes have been identified in some settings (Cluver et al., 2016; Gibbs et al., 2012; Manley et al., 2013). In addition, programme design and implementation may have different intended and unintended consequences for women and men at varying ages and stages of their life (Holmes & Jones, 2010).

The current evidence on the benefits and risks of social protection across gender (i.e. girls, boys, women and men) in LMICs is yet to be consistently appraised and systematically examined. Evidence on whether the impact of different social protection programmes differ by gender has not been synthesised and analysed. Research is needed on the factors that determine these differential impacts. Notably, questions remain as to whether programme outcomes vary according to intervention implementation and design. As a result, governments and organisations seeking to design, implement, de‐implement, scale up, down or close social protection programmes face challenges when examining the evidence on social protection as a whole and its impact on gender equality indicators.

A systematic review of reviews is needed to generate a clearer picture of the differential impact of social protection on women and men and translate this knowledge into policy actions that improve gender equality outcomes across the life course. The primary aim of this review is therefore to synthesise evidence from systematic reviews on the differential gender impacts of social protection programmes. In doing so, this review places itself at the intersection of sustainable development goals (SDGs) 1 (end poverty in all its forms everywhere) and 5 (achieve gender equality and empower all women and girls). In addition, this review will inform specific targets within the rest of the SDG Agenda, such as health (target 3.8), decent work and economic growth (target 8.5) and equality (target 10.4). In the context of meeting these goals, it will synthesise the evidence on social protection by gender to inform the use, design and implementation of programmes in LMICs, contribute to building the evidence base of the 2030 Agenda for Sustainable Development and strengthening national initiatives for achieving gender equality and reducing poverty.

### 1.1 Description of the intervention

More than half of the global population is not effectively protected by any type of social protection benefit, with very low coverage in Africa and Asia and the Pacific (16% and 32%, respectively) compared to Europe and Central Asia, and the Americas (82% and 61%, respectively) (International Labour Organization, 2017). Social protection coverage for children is also insufficient: approximately only one in three children (35%) are covered, pointing to significant underinvestment in children and families (Ortiz et al., 2017). Only 41% of women with new‐borns receive maternity cash benefits that provide them with income security during this critical period. Effective pension coverage for older persons stands at 68% of all persons above retirement age worldwide (Ortiz et al., 2017).

However, in LMICs investment and interest in these measures is rising. The number of LMICs with social safety nets has doubled from 72 to 149 in the last two decades (World Bank Group, 2017). Examples of such social protection programmes include: food for education programmes (Tanzania), scholarships for low‐income families (Guatemala), food baskets for Internally Displaced Persons (Libya), electricity and fuel subsidies for low‐income households (Cambodia), and noncontributory old age pensions (Mexico).

While there is no single definition of social protection, it is hereby understood as “a set of policies and programmes aimed at preventing or protecting all people against poverty, vulnerability and social exclusion throughout their lifecycle, with an emphasis towards vulnerable groups” (Social Protection Inter‐Agency Cooperation Board [SPIAC‐B], 2012; UNICEF, 2019, p. 2). As such, social protection aims to both avert and provide relief from poverty and adversity (Devereux and Sabates‐Wheeler, 2004). Social protection programmes can be provided by public organisations or bodies with or without collaboration of nongovernmental organisations or private institutions. Programmes implemented solely by private organisations or nongovernmental organisations without government affiliation are not considered part of social protection (UNICEF, 2019). The recipient and duration of social protection programme varies according to the conditions and socioeconomic disadvantages each programme aims to address. The field of social protection can be conceptualised or divided into four areas or categories (i.e. social assistance, social insurance, labour market programmes and social care) drawing from various international categorisations such as the Inter‐Agency Social Protection Assessment and UNICEF Global Social Protection Framework (SPIAC‐B, 2012; UNICEF, 2019, p. 2), as defined in Table 1.

**Table 1:** Social protection categories, definitions and examples

Category	Definition	Examples
Social assistance	Cash and near cash benefits, in‐kind benefits, where receipt is not determined by individual contributions (i.e. noncontributory and publicly provided)	*For vulnerable/poor*: Conditional and unconditional cash or near cash transfers. Near cash transfers such as food vouchers. Conditional and unconditional in‐kind transfers (e.g., food parcels, layettes) *For parents/caregivers/family*: Childcare cash benefits/grants, birth grants, family allowances, maternity and paternity benefits (e.g., cash benefits for pregnant and lactating women and girls, parents, parents on parental leave), death benefit, child benefit after divorce. *For income guarantee*: Universal basic income, minimum income guarantee schemes *For unemployment*: Noncontributory unemployment benefits *For shelter*: Housing subsidies *For old age and disability*: Disability grants, social pensions, sick leave *Tax breaks for social purposes* (e.g., childcare, care for the elderly) *For encouraging access to social services* (e.g., fee waivers for health care, fee waivers for schooling, school vouchers, school feeding)
Social insurance	Cash or near cash benefits where eligibility is determined based on personal contributions or employer contributions (i.e. contributory schemes)	*For the parents/caregivers/family*: Birth payments/benefits, maternity, paternity and parental leave, childcare cash benefits and family allowances (e.g. for public servants) *For unemployment*: Unemployment benefits/insurance for former employees *For illness, injury, death*: Health insurance *For shelter*: Housing subsidies for employees, household contents insurance *For old age*: Retirement pensions
Labour market programmes	Programmes and services that support employment and livelihoods and enable families to have enough income while ensuring provision and time for quality childcare.	*For hiring/encouraging employment*: Job search programmes, hiring subsidies, wage subsidies. *Direct job creation*: public works programmes, temporary alternative employment schemes, *Skills development*: Job training or skills development
Social care services	Direct outreach, case management and referral services to children and families	*For pregnancy/birth*: Prenatal and post‐natal services [not primary or secondary health care] *For family*: Family supports (e.g., parenting education, IPV interventions, centred based childcare, after school clubs) *For children and older dependents*: Care for children or older people

### 1.2 How the intervention might work

**Figure 1:**
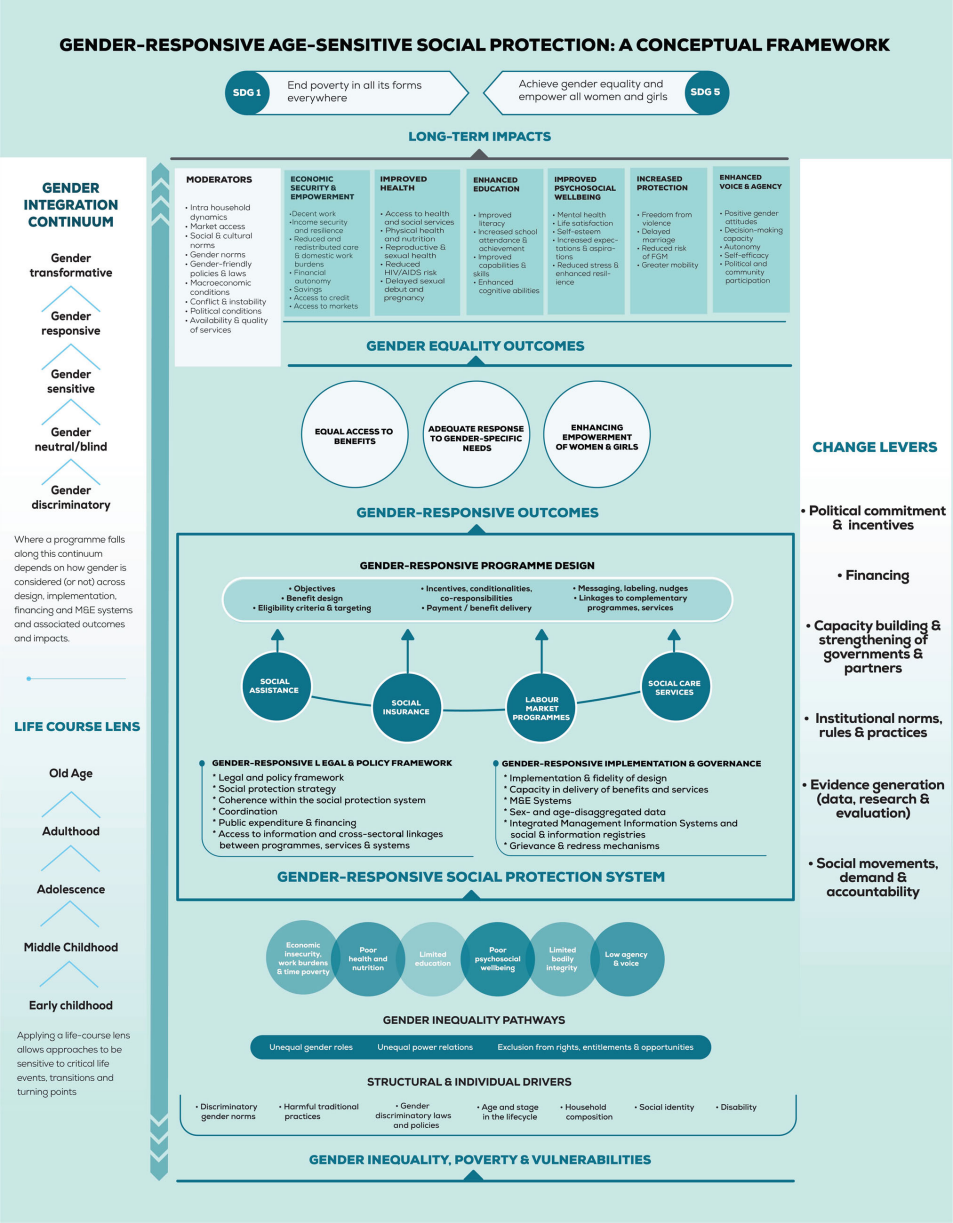
Gender‐Responsive Age‐Sensitive Social Protection: A Conceptual Framework

The Gender‐Responsive Age‐Sensitive Social Protection Conceptual Framework (Figure 1—Reprinted with authors’ permission) guides this review and delineates *how* social protection is hypothesised to lead to poverty reduction and promote long‐term and sustained gender equality (UNICEF Office of Research—Innocenti, 2020). Building on existing conceptual and theoretical efforts (Holmes & Jones, 2013), the framework starts by acknowledging that poverty, risks and vulnerabilities are gendered, can change at different transitions and turning points throughout the life course, as well as accumulate over time. It reflects structural and individual‐level drivers of gender inequality that result in unequal outcomes for girls and women relative to boys and men, with long‐term negative impacts for them, and for sustainably reducing poverty and enhancing gender equality. It outlines moderating factors, which are dependent on context and programme design components. Integrating analysis by age and gender allows for a life course lens on gendered inequalities in relation to poverty and vulnerability.

Second, the framework maps out the opportunities and mechanisms through which social protection systems may address gendered risks and vulnerabilities through specific programmes across the social protection delivery cycle, including the legal and policy framework, programme design, implementation, governance and financing. The conceptual framework deliberately takes a macro‐view, acknowledging the importance of a systemic and institutional perspective, beyond project or programme level pathways.

Third, the framework applies a Gender Integration Continuum (GIC), a tool to distinguish different degrees of integration of gender considerations across the social protection delivery cycle, ranging from gender‐discriminatory to gender‐transformative. The GIC helps assess the extent to which social protection systems and programmes are designed and delivered in a way that explicitly addresses gender inequality. It is based on a recognition that programmatic or policy attention to addressing gender inequality depends to a great extent on the prior understanding of prevailing gender inequalities and norms that need to be transformed through purposive actions. It thus shows how gender‐responsive social protection, by specifically addressing gendered poverty, risks and vulnerabilities, can strengthen social protection system‐level outcomes, such as improved coverage and adequacy of social protection systems, as well as individual programme results, and thereby contribute to a range of gender equality outcomes, including economic security and empowerment, improved health and enhanced education. In turn, the achievements of social protection are conceptualised to contribute to SDGs 1 and 5.

### 1.3 Why it is important to do this review

Systematic reviews summarise the best available evidence relevant to a specific research question. They are the most comprehensive way to collate all the relevant evidence on a specific topic or theme (Bakrania, 2020). The accelerated increase of systematic review publishing creates a growing interest in summarising and analysing systematic reviews. Systematic reviews of reviews help gather a wide range of evidence on interventions, enable large comparisons and can help clarify discrepant systematic review results (Polanin et al., 2017). By considering only the highest level of evidence (i.e. systematic reviews), they offer a means to review the evidence base and to obtain a clear understanding of a broad topic area (Aromataris et al., 2015). In addition, systematic reviews of reviews provide conclusions regarding research trends and gaps, making them also useful for researchers (Duvendack & Mader, 2019; Polanin et al., 2017).

There is a large body of empirical evidence, including systematic reviews, investigating the impact of social protection programmes. A myriad of robust systematic reviews have sought to clarify the impact of social protection programmes on women and men, regardless of their age (Baird et al., 2014; Bassani et al., 2013; Bastagli et al., 2016; Buller et al., 2018; Chinen et al., 2017; Dickson & Bangpan, 2012; Durao et al., 2020; Haberland et al., 2018; Kalamar et al., 2016; Kluve et al., 2017; Langer et al., 2018; Målqvist et al., 2013; Murray et al., 2014; Pega et al., 2015; Tripney et al., 2013; van Hees et al., 2019; Yoong et al., 2012). The results, however, are dispersed with reviews focusing on various specific subtypes of social protection (e.g., labour market programmes, cash transfers), women and/or men, in different regions, and with some offering conflicting or discordant results regarding the impact of social protection measures. Although various systematic reviews have gathered evidence on various areas of social protection in LMICs, evidence on the whole field is yet to be examined. Table 2 presents an overview of relevant systematic reviews on social protection identified through a scoping exercise.

**Table 2:** Overview of systematic reviews on social protection

Reference	Time range	Population	Type of programme
Baird et al. (2014)	1997–2013	Children and young women and men, ages 5–22	Social assistance
Bassani et al. (2013)	Until 2012	Mothers and children	Social assistance
Bastagli et al. (2016)	2000–2015	No restrictions	Social assistance
Buller et al. (2018)	1998–2015	No restrictions	Social assistance
Chinen et al. (2017)	1990–2016	Adult women	Labour market
Dickson and Bangpan (2012)	2003–2010	Girls and young women, ages 10–24	Social assistance and labour market
Durao et al. (2020)	1980–2020	No restrictions	Social assistance
Haberland et al. (2018)	1990–2014	Girls and young women, ages 10–24	Social assistance and labour market
Kalamar et al. (2016)	2000–2015	Young women and men, ages 10–24 years	Social assistance and labour market
Kluve et al. (2017)	1990–2014	Young women and men, ages 15–35	Labour market
Langer et al. (2018)	1990–2017	Women ≥ 15 years old	Labour market
Målqvist et al. (2013)	2001–2012	Mothers and children (girls and boys)	Social assistance
Murray et al. (2014)	1990–2012	Mothers and children (girls and boys)	Social assistance
Pega et al. (2015)	Until 2014	No restrictions	Social assistance
Tripney et al. (2013)	2000–2011	Young women and men, ages 15–24	Labour market
van Hees et al. (2019)	1995–2018	Vulnerable groups	Social insurance
Yoong et al. (2012)	1990–2010	No restrictions	Social assistance

We identified 17 relevant systematic reviews based on a wide range of social protection interventions, mostly including studies conducted between 1990 and 2018, and only three including studies published prior to 1990. Most of these reviews (*N* = 12) focused on the effectiveness of social assistance programmes (including cash transfers—conditional and unconditional, voucher schemes and in‐kind benefits) on a broad spectrum of socioeconomic and well‐being outcomes. Seven of these analysed labour market policies (including off and on‐the job training, job placement, livelihood programmes, labour subsidies and entrepreneurship promotion) with three reviews identifying other outcomes (e.g., health, well‐being, quality of care). Only one review focusing on social insurance (health) was identified and none were found for social care programmes.

Though this has not been a common element of systematic reviews, more efforts are being put to a more nuanced analysis of design and implementation features (see Bastagli et al., 2016; Chinen et al., 2017; Haberland et al., 2018). Other reviews have also included design and contextual considerations in their analysis, with several them assessing the feasibility, appropriateness, as well as effectiveness of programmes (see Dickson & Bangpan, 2012; Murray et al., 2014).

Some of the design features for which we expect to provide some evidence of differential gender effects and gender‐equality outcomes are: targeting, duration and exposure to intended benefit (Bastagli et al., 2016), included actors beyond target population (multilevel), size of targeted population (saturation) and complexity of intervention (e.g., single or multicomponent; booster activities) (Haberland et al., 2018). Other addressed features, as described by the authors include: delivery mechanisms (Pega et al., 2015), exposure to labour market participation enhancing social norms (e.g., conducive norms at work, affirmative action programmes), labour demand‐led intervention design (e.g., consultation of private sector needs), gender‐sensitive intervention design (e.g., women‐only programmes, childcare facilities), provision of soft/life skills and social empowerment training (e.g., confidence/aspiration building, noncognitive work skills), participation profiling (e.g., narrow eligibility criteria, gender‐sensitive marketing), clear governance structures for intervention providers (e.g., payment by results, provider monitoring), flexibility and responsiveness in intervention implementation and design (e.g., information management, piloting and iteration) (Langer et al., 2018). Pega et al. (2015) also acknowledge implementation issues in their use of the concept “fuzzy UCTs” or cash transfers that are unconditional in practice. Other authors considered the role of contextual factors (e.g., time constraints, distance, cost of transport, economic and labour market conditions) (Chinen et al., 2017).

A systematic review of reviews will allow us to identify patterns within and across programme types and outcomes to understand *whether* and *how* social protection programmes distinctively benefit women and men. This systematic review of reviews will generate a clearer picture of the differential impact of social protection on women and men and translate this knowledge into policy actions that improve gender equality outcomes across the life course. As such, this review aims to inform the decisions of donors, policymakers and programme managers seeking to establish gender‐responsive social protection programmes. More specifically, the findings of this review will provide valuable insights for different component of UNICEF's and strategic partners’ programmes. For example, by informing discussions on cost‐effectiveness of programmes and gender equality measures and highlighting which evidence is informative in certain contexts. In doing so, the review will also offer clarity as to the research gaps to be investigated in future studies.

To enable this knowledge transfer, the findings and conclusions of this review will be shared *via* a policy brief, a blog piece in Evidence for Action, webinars, meetings with the review's Advisory Group and a series of posts and videos available on UNICEF Innocenti's online platforms. Working with UNICEF country offices, this information will be actively shared with country‐level stakeholders such as government, civil society organisations and other UN agencies, to create interest, buy‐in and engagement on gender and social protection. This is part of our research uptake objectives of anchoring research translation and use at the country level through co‐creation and early stakeholder engagement.

This systematic review is part of a research programme investigating Gender‐Responsive Age‐Sensitive Social Protection (GRASSP) systems to enhance gender equality outcomes in low and middle‐income settings. Therefore, this review is guided by the feedback and input of the GRASSP External Advisory Group (EAG). The group is composed of academics and practitioners, with expertise on social protection and gender, from UNICEF and partner organisations as well as academic institutions, including ILO, ODI, LSE and the World Bank. The EAG provides expert advice on the subject areas of the review (i.e. gender and social protection) throughout all the steps of the systematic review process. The role of the EAG includes revising the search strategy, identifying systematic reviews not retrieved through the searches, providing feedback on the results of the review, as well as providing suggestions for increasing uptake and communication of findings.

## 2 OBJECTIVES

This review aims to systematically collect, appraise, map and synthesise the evidence from systematic reviews on the differential gender impacts of social protection programmes in LMICs. Therefore, it will answer the following questions:

1. What is known from systematic reviews on the gender‐differentiated impacts of social protection programmes in LMICs?
2. What is known from systematic reviews about the factors that determine these gender‐differentiated impacts?
3. What is known from existing systematic reviews about design and implementation features of social protection programmes and their association with gender outcomes?

Systematic reviews have sought to clarify the impacts of social protection programmes on gender outcomes as well as aspects of their design and implementation, using quantitative and qualitative findings from primary studies. Therefore, we adopt a broad scope to synthesise evidence from reviews investigating impact, contextual factors, design and implementation features of social protection programmes, regardless of their methodology or epistemological approach. Based on these findings, we will report and reflect on the implications for policy, programming and future research.

## 3 METHODS

### 3.1 Criteria for considering studies for this review

#### 3.1.1 Types of studies

We will include systematic reviews, irrespective of publication status and the language they were published in, that synthesise and analyse evidence from qualitative, quantitative or mixed‐methods studies. As defined by the Campbell Collaboration: “A systematic review summarises the best available evidence on a specific question using transparent procedures to locate, evaluate, and integrate the findings of relevant research” (The Campbell Collaboration, 2014, p. 6). In addition, we will adopt the following additional criteria, as outlined by Higgins and Green (2011):

- A set of clearly stated objectives and predefined eligibility criteria.
- A methodology that is clearly defined allowing reproducibility.
- A search strategy that allows the identification of studies meeting the predefined eligibility criteria.
- A quality appraisal of included studies.
- A systematic synthesis, including systematic reviews that adopt a meta‐analytical, narrative or thematic approach.

Of note, meta‐analyses (understood as statistical aggregations of primary study outcomes aimed at producing a pooled summary effect) might be published separately from its broader systematic review. Said studies will also be included in the review if the broader systematic review is identified. Some of the systematic reviews in our study sample might be published in multiple journals. If so, these will be treated as duplicate reviews with data extracted from the most comprehensive review. Should we identify multiple versions of the same systematic review, we will only include the latest updated review. We will preliminarily retrieve all protocols of systematic reviews and exclude them once the full review is identified. When the corresponding systematic review is not identified, authors will be contacted to ask whether the relevant reviews of interventions are close to completion and assess prepublication version for inclusion in our systematic review of reviews. Other systematic reviews of reviews identified through our search will be excluded.

#### 3.1.2 Types of participants

We will include systematic reviews that analyse the outcomes of social protection programmes on women, men, girls and boys in LMICs. As we are interested on the impacts of social protection during different stages of the life course, we will not set any restrictions on age. The reviews outlined in Table 2 include primary studies with diverse populations: mothers and children, girls and young women, young women and men, adult women, school‐age children and their families, adult women or vulnerable groups. Five of the reviews do not impose restrictions based on gender but offer sex‐disaggregated results. Studies that do not report gender‐disaggregated results of the impact of these programmes will be excluded.

#### 3.1.3 Types of interventions

To be included in this study, systematic reviews should investigate one or more types of social protection programmes. Based on the reviews presented in Table 2, we foresee that most systematic reviews will focus on either an intervention sub‐type (e.g., cash or resource transfer, active labour market interventions) or on a wide range of interventions aiming to mitigate or improve a specific social protection outcome (e.g., health equity, access to food, employment). Where systematic reviews include complex or integrated interventions combining social protection and other development programmes, we will only consider the findings of social protection programmes. If the results specific to social protections cannot be disaggregated, we will highlight this as a possible confounding factor. Based on the scoping exercise (Table 2), we expect most available systematic reviews to investigate social assistance and labour market interventions with only a few reviews focusing on social insurance and social care programmes. No restrictions will be imposed on intervention comparison.

#### 3.1.4 Types of outcome measures

As previously discussed, our review is informed by the Gender‐Responsive Age‐Sensitive Social Protection Conceptual Framework, which establishes the following outcome areas of gender equality:

- Economic security and empowerment: Right to access opportunities and decent work, including the ability to participate equally in existing markets; control over and ownership of resources and assets (including one's own time); reduced burden of unpaid care and domestic work, and meaningful participation in economic decision‐making at all levels.
- Improved health: Right to live healthily, including sexual and reproductive health rights, and right to access safe, nutritious and enough food. This is also concerned with information, knowledge and awareness of health issues, and access to and expenditure on health services.
- Enhanced education: Right to inclusive and equitable quality education, leading to relevant and effective learning outcomes, including cognitive skills and knowledge; right, access to and expenditure on lifelong learning opportunities.
- Improved mental health and psychosocial well‐being: A state of complete physical, mental and social well‐being and not merely the absence of disease or infirmity, in which an individual realises their own abilities, can cope with the normal stresses of life, can work productively and is able to make a contribution to his or her community.
- Increased protection: Freedom from all forms of violence (physical, sexual, and psychological violence, including controlling behaviour), exploitation, abuse, and neglect, including harmful practices (e.g., child, early and forced marriage, FGM) and child labour (including children's unpaid care and domestic work).
- Enhanced voice and agency: Ability to speak up and be heard, and to articulate one's views in a meaningful way (voice), and to make decisions about one's own life and act on them at all levels (agency).

In this systematic review of reviews, we will include all systematic reviews that investigate any outcomes within any of these core areas. The use of core outcome areas has been recommended as a strategy to prevent loss of information in systematic reviews (Saldanha et al., 2020). Narrowing down our study to a specific set of gender outcomes (e.g., increased school attendance, delayed marriage, income security) could result in missed opportunities to understand the impact of social protection on gender equality. In addition, choosing broad outcome areas will allow us to put forward specific suggestion for expanding the GRASSP Conceptual Framework. Examples of outcomes of the systematic reviews presented in Table 2 include: economic engagement, well‐being, child marriage indicators, school enrolment and attendance and access to nutritious food. We will not distinguish between primary or secondary outcomes. We will report on any adverse and unintended effects on these outcomes, factors and programme design and implementation features determining the outcomes of social protection programmes. Implementation is understood as the process of fulfilling or carrying out a social protection intervention into effect (Peters et al., 2014). Intervention design or development is the period or process of developing an intervention to “the point where it can reasonably be expected to have worthwhile effect” (Craig & Petticrew, 2013, p. 9) that usually consists of making decisions about the content, format and delivery and ends with the production of a document or manual describing the intervention and how it should be delivered (O'Cathain et al., 2019). While only six of the reviews presented in Table 2 explicitly investigate the links between programme design and implementation and its outcomes, six other reviews summarise key considerations on contextual factors as well as programme design and implementation features, with a number of them assessing feasibility and appropriateness, as well as programme effectiveness (Dickson & Bangpan, 2012; Murray et al., 2014).

##### 3.1.4.1 Primary outcomes

We will not distinguish between primary or secondary outcomes and we will not impose restrictions based on the duration of follow‐up.

###### 3.1.4.1.1 Types of settings

The reviews included in our systematic review of reviews will investigate social protection in LMICs, as defined by the World Bank in 2019 (Cochrane—Effective Practice and Organisation Care, 2020). Where systematic reviews and meta‐analyses include evidence from high‐income countries, we will only consider the findings that are presented for LMICs; we will also consider systematic reviews covering particular regions within LMICs (e.g., Sub‐Saharan Africa,). Reviews that do not disaggregate results by country, region or national income level will not be included.

###### 3.1.4.1.2 Timeframe

A seminal report published in 2010 titled *Rethinking social protection using a gender lens*, identified the need to systematically appraise the evidence on social protection and gender equality (Holmes & Jones, 2010). Since the report points to the absence of systematic reviews on the field, will limit our searches to 2010 onwards.

### 3.2 Search methods for identification of studies

Our search strategy aims to find both published and unpublished literature from a wide range of sources (i.e. bibliographic databases, institutional websites and libraries) (Kugley et al., 2017). The search techniques we will use are: subject searching, reference list checking, citation searching and contact with experts.

#### 3.2.1 Electronic searches

We will gather evidence from systematic reviews on the impact of these programmes on gender‐related outcomes as well any available evidence on the design and implementation of these interventions.

The following academic databases will be searched:

- Web of Science
- Academic Search Complete (EBSCO)
- International Bibliography of the Social Science Database via ProQuest
- Africa‐Wide via EBSCOHOST
- ERIC (Education Resources Information Centre)
- Medline Complete via EBSCOHOST
- PsycINFO via EBSCOHOST
- EconLit via EBSCOHOST

In addition, a search for more reviews, especially unpublished studies and grey literature will be conducted search terms adapted for the following institutional websites, libraries and sources of grey literature:

- Campbell Collaboration Library
- World Bank eLibrary (https://elibrary.worldbank.org/)
- EPPI‐Centre
- IDEAS/RePEC (https://ideas.repec.org/)
- 3ieimpact evidence portal
- ILO (International Labour Organisation)
- SSRN (Social Science Research Network)
- Research for Development Outputs (https://www.gov.uk/research-for-development-outputs)
- Asian Development Bank (https://www.adb.org/about/library)
- Africa Centre for Evidence—Systematic Review Repository
- Social Systems Evidence (socialsystemsevidence.org)

We ran searches in Web of Science (3,860hits), Academic Search Complete (370hits), Social Science database (39hits), Africa‐Wide via EBSCOHOST (297hits) World Bank elibrary (127hits), ERIC (50hits), Medline Complete (54hits), PsycINFO (391hits) and EconLit (188hits). The search strategies were developed using keywords and index terms (controlled vocabulary) relevant to the study concepts. Each search strategy consisted of the study concepts divided into four parts: intervention and related terms (adapted from the GRASSP Conceptual Framework—Figure 1), study design (search filter for systematic review database by 3ie), population and LMICs (adapted from the Cochrane EPOC LMICs filter updated in 2020). The search strings were adapted for each database to retrieve all systematic reviews published within the last 10 years with no language restrictions. The search strategies, as well as the databases and websites included in this review were revised by members of the Advisory Group. See Appendices A, B, C, D, E, F, G, H for the full search strategies of academic databases.

#### 3.2.2 Searching other resources

Reference lists of included reviews will be screened to identify additional, potentially relevant, records. Experts from the EAG will be consulted to identify more systematic reviews not retrieved through the database and websites searches.

### 3.3 Data collection and analysis

#### 3.3.1 Description of methods used in systematic reviews

Eight reviews presented in Table 2 employed some form of qualitative synthesis (e.g., narrative synthesis, descriptive summary, meta‐synthesis) to analyse primary studies. Three reviews used a combination of qualitative synthesis and meta‐analysis while six conducted meta‐analyses of results. Therefore, we expect the selected systematic reviews to include primary studies that employ quantitative, qualitative and mixed‐methods approaches.

#### 3.3.2 Criteria for determination of independent reviews

A prevalent challenge of systematic reviews of reviews is the inclusion of systematic reviews that address similar research questions or synthesise evidence on similar and/or related interventions, which, may include some of the same underlying primary studies. The potential for “overlap” in primary studies between included systematic reviews introduces a risk of bias, by including the same primary study's results multiple times. Although at present the Campbell Collaboration does not provide specific guidance on how to manage the issue of overlap, former systematic reviews of reviews have addressed this issue in various ways. These approaches include: (1) considering nonoverlapping reviews only; (2) extracting results from primary studies to avoid double‐counting or repetition in synthesis; (3) assessing primary study overlap (e.g., citation matrix) and discussing results in relation to the identified overlap (Duvendack & Mader, 2019; Polanin et al., 2017; Pollock et al., 2021). The most appropriate approach in this review considering its objectives, synthesis method as well as time and resource constraints is to assess the degree of overlap. As suggested by Pollock et al. (2021), the degree of overlap will determined by:

- Creating a citation matrix to visually demonstrate percentage of overlap across each of the four intervention areas
- Computing the Corrected Covered Area (CCA) (Pieper et al., 2014) as a measure of overlap by dividing the frequency of repeated occurrence of the index publication in other reviews by the product of index publications and reviews, reduced by the number of index publications
- Describing the percentage of overlapping primary studies and CCA, and discussing whether and how overlap affects the results reported in the systematic review of reviews

This systematic review of reviews will adopt the PRIO‐harms reporting checklist (Bougioukas et al., 2018) which considers the points above as standard items for reporting overlap in systematic reviews of reviews.

When discussing possible overlap, is also important to consider independence from other systematic reviews of reviews. Duvendack and Mader (2019) conducted the first systematic review of reviews of the Campbell Collaboration and set a precedent for the use of the systematic review of reviews methodology to better inform the decisions of development donors, policymakers and programme managers. This systematic review of reviews analysed the impact of financial inclusion in LMICs. Along with financial inclusion, social protection is a widely recognised and funded area of international development. Although both reviews are complementary, they will constitute independent reviews. We do not foresee an overlap on the included studies as microfinance and savings intervention—the focus of Duvendack and Mader (2019)'s review—are not considered elements of social protection. Overlaps may occur with studies simultaneously examining financial inclusion and social protection interventions. In the event of an overlap, it will be reported as part of the limitations section and findings will be contrasted with those reported by Duvendack and Mader (2019).

#### 3.3.3 Selection of studies

A review author (O. O.) developed the search terminology. The screening process and checklist (Appendix I) was pilot tested at title, abstract and full text with the 17 studies identified through the scoping exercise. Two review authors (C. P. and A. I.) will independently screen titles and abstracts and full texts (double‐blind screening). Disagreements will be solved by a third author (S. B.), who will independently revise all included studies to confirm the inclusion decision. A PRISMA diagram will be used to summarise the study selection process and a list of excluded studies will be included in the appendix.

#### 3.3.4 Data extraction and management

Data from each study will be extracted by two authors (C. P. and A. I.) in EPPI‐Reviewer 4. The data extraction framework was pilot tested with the 17 studies identified through the scoping exercise. Reviewers will randomly check each other's work to ensure data was correctly extracted. Discrepancies will be resolved by consensus or by a third author (S. B.) when consensus is not reached.

#### 3.3.5 Assessment of risk of bias in included studies

The methodological quality of the included systematic reviews will be assessed employing the Joanna Briggs Institute (JBI) Critical Appraisal Checklist for Systematic Reviews and Research Syntheses’ (Aromataris et al., 2015). We considered a range of critical appraisal tools (e.g., AMSTAR 2, 3ie Critical Appraisal Checklist, ROBIS) and concluded that the JBI tool provided an inclusive approach to appraising quantitative, qualitative and mixed‐method systematic reviews. This checklist has been developed and piloted by JBI's umbrella review methodology working group and has been used in other reviews of systematic reviews. The JBI checklist includes various considerations for the extent to which a systematic review addresses the possibility of bias in its design, conduct and analysis. These considerations include language and publication bias in the search strategy; approaches to minimising systematic errors in the conduct of the systematic review; and whether recommendations are supported by results.

The JBI Critical Appraisal Checklist has 11 criteria:

1. Is the review question clearly and explicitly stated?
2. Were the inclusion criteria appropriate for the review question?
3. Was the search strategy appropriate?
4. Were the sources and resources used to search for studies adequate?
5. Were the criteria for appraising studies appropriate?
6. Was critical appraisal conducted by two or more reviewers independently?
7. Were there methods to minimise errors in data extraction?
8. Were the methods used to combine studies appropriate?
9. Was the likelihood of publication bias assessed?
10. Were recommendations for policy and/or practice supported by the reported data?
11. Were the specific directives for new research appropriate?

Each of the questions posed in the checklist can be scored as being “met”, “not met”, “unclear” or “not applicable”, which allows raters to make a broad assessment of the quality of included reviews (Aromataris et al., 2015). Reviews will be awarded a score of 1 for each checklist criteria clearly met and 0 for those not met or unclear, with a maximum possible score of 11. Reviews scoring 8–11 will be categorised as high quality, those scoring 4–7 as moderate, and 0–3 as low‐quality systematic reviews. Reviews consider to be of low‐quality will be excluded. The quality of the systematic reviews included in the review will be appraised independently by two authors (C. P. and S. B.). Interclass correlation coefficient will be used to estimate the degree of agreement or consensus among raters, using Fleiss interpretation (Fleiss, 1971).

#### 3.3.6 Measures of treatment effect

As suggested by Pollock et al. (2021), we will extract the pooled effect sizes of gender outcomes of meta‐analyses as reported by the review authors in our data extraction table (Table 3). These effect sizes will be presented as Supporting Information and will not be presented side by side to avoid comparisons across systematic reviews.

**Table 3:** Data extraction framework

Category	Data items
Context	Source Author Publication year Income level: low; middle income; global Countries
Population	Age group: children (0–4); children (5–9); early adolescence (10–14); older adolescence (15–19); adult 20–64; old age (65+); age range [state] Recipient of programme: female; male; other [state]; subgroup [state] Targeted indirect beneficiaries: female; male; other [state]; subgroup [state]
Intervention	Type of social protection programme(s): social assistance [state]; social insurance [state]; labour market [state]; social care [state]; other [state] Intervention name [state] Duration of intervention [state] Institution(s) providing the programme(s): government agency; government agency and nongovernmental organisation; government agency and private organisation; other [state]
Methodology	Type of review: systematic review; systematic review with meta‐analysis; rapid evidence assessment; rapid review; review protocol Type of analysis: quantitative [state]; qualitative [state] Number of included studies [state] Review time range [state] Types of included studies: experimental; quasi‐experimental; observational cross‐sectional; observational longitudinal; qualitative; mixed methods; other [state] Measurements [state] Outcome(s) and outcome area: economic security and empowerment; improved health; enhanced education; improved mental health and psychosocial well‐being; increased protection; enhanced voice and agency; other [state] Comparison [state] Risk of bias used in review
Quality appraisal	Is the review question clearly and explicitly stated? (met; not met; unclear; not applicable) Were the inclusion criteria appropriate for the review question? (met; not met; unclear; not applicable) Was the search strategy appropriate? (met; not met; unclear; not applicable) Were the sources and resources used to search for studies adequate? (met; not met; unclear; not applicable) Were the criteria for appraising studies appropriate? (met; not met; unclear; not applicable) Was critical appraisal conducted by two or more reviewers independently? (met; not met; unclear; not applicable) Were there methods to minimise errors in data extraction? (met; not met; unclear; not applicable) Were the methods used to combine studies appropriate? (met; not met; unclear; not applicable) Was the likelihood of publication bias assessed? (met; not met; unclear; not applicable) Were recommendations for policy and/or practice supported by the reported data? (met; not met; unclear; not applicable) Were the specific directives for new research appropriate? (met; not met; unclear; not applicable)
Review findings—Research question 1	Results by outcome area:
	Economic security and empowerment [state] Improved health [state] Enhanced education [state] Improved mental health and psychosocial well‐being [state] Increased protection [state] Enhanced voice and agency [state] Other [state]
	If quantitative findings (gender):
	Type, magnitude and direction of effect size [state] Summary of cost‐effectiveness findings [state]
Review findings—Research question 2	Moderators:
	Intra‐household dynamics [state] Access to market [state] Social and cultural norms [state] Gender norms [state] Gender‐responsive policies and laws [state] Macroeconomic conditions [state] Conflict and instability [state] Political conditions [state] Availability, accessibility, affordability, acceptability and quality of services [state] Other [state]
	Structural and individual drivers:
	Discriminatory gender norms [state] Harmful traditional practices [state] Gender discriminatory laws and policies [state] Age and stage in the lifecycle [state] Household composition [state] Social identify [state] Disability [state] Other [state]
Review findings—Research question 3	Design factors:
	Objectives [state] Benefit design [state] Eligibility criteria and targeting [state] Incentives, conditionalities, coresponsibilities [state] Payment/benefit delivery [state] Messaging, labelling and nudges [state] Linkages to complementary programme services [state] Other [state]
	Implementation factors:
	Implementation and fidelity of design [state] Capacity in delivery of benefits and services [state] M&E [state] Sex‐ and age‐disaggregated data [state] Integrated management information systems and social and information registries [state] Grievance and redress mechanisms [state] Other [state]
Other	Recommendations [state] Research gaps [state]

#### 3.3.7 Unit of analysis issues

We will primarily be extracting information at the systematic review level, as outlined in Table 3. However, when only a subset of the studies included in a review meet our inclusion criteria, data will be extracted from the results that relate to said studies. To ensure that this data refers to the specific studies, extracted data will be cross‐checked with the primary study. Lastly, we will extract results from systematic reviews as reported by the review authors.

#### 3.3.8 Dealing with missing data

The original authors of included systematic reviews will be contacted where data is missing or insufficiently reported. When authors cannot be contacted, we will note the gap in coverage and state that certain data is not available through one or more systematic reviews.

#### 3.3.9 Assessment of reporting biases

One of the items on the JBI checklist (criteria 9) assesses whether the review authors carry out an investigation of publication bias and discuss the impact this had on their review findings. Any other observations relating to other types of reporting biases (e.g., language, location, citation, outcome reporting biases) will be noted and addressed in the discussion section of the review.

#### 3.3.10 Data synthesis

This systematic review of reviews will adopt framework synthesis as the synthesis method. There are several reasons why this approach is suitable for this review. First, it can be applied to reviews of complex interventions and where there is a broad thematic scope (Brunton et al., 2020; Snilstveit et al., 2012). This review encompasses an entire domain of interventions (social protection), which itself comprises of multiple intervention types and subtypes. The GRASSP Conceptual Framework (Figure 1) illustrates how complex the linkages and pathways are between interventions and gender outcomes. Secondly, as Flemming et al. (2020) argue, framework synthesis allows for the juxtaposition of quantitative and qualitative evidence. Our review seeks to address the differential impacts of social protection programmes according to gender and age, the factors that determine those impacts and the circumstances under which the intervention might work. The GRASSP Conceptual Framework will serve as a “scaffold” for collating quantitative and qualitative evidence on complex social protection interventions from different types of reviews. The approach to framework synthesis described by Brunton et al. (2020) consists of three steps, as summarised below.

##### 3.3.10.1 Framework selection and familiarisation

Prior work undertaken within the broader GRASSP programme, in scoping the literature and developing the GRASSP Conceptual Framework constitutes part of this stage. The framework proposes a systematic, holistic and integrated approach for conceptualising the intersections between gender and social protection. It provides this review with a typology of social protection interventions and gender equality outcome areas, as well as delineating the structural and individual drivers, moderators and design and implementation elements that may determine gender outcomes. It was developed through a review of the literature and refined through consultations with gender and social policy experts. The current framework builds on and expands existing conceptual and theoretical efforts focused on integrating a gender lens into public policy (UNICEF Office of Research—Innocenti, 2020). The scope of this review is determined by the GRASSP Conceptual Framework. This process, along with the previously described scoping exercise, contributed to the review team's familiarisation with the selected framework.

##### 3.3.10.2 Indexing and charting

The GRASSP Conceptual Framework provides a basis for searching for, screening and extracting data from included reviews. The search strategy translates the key concepts from the typologies of interventions and outcomes. Our approach to the data, themes and categories to be coded are driven by the way in which the interventions, outcomes, structural and individual drivers, moderators and design and implementation factors are represented in the framework. Data extraction (Table 3) draws directly from the typologies contained within the framework. This provides us with an initial scaffolding for grouping characteristics from each review into categories and deriving themes from this data.

Framework synthesis is iterative in nature (Petticrew et al., 2013) and therefore allows for both a deductive and inductive approach to synthesis. This allows to extract and synthesise data from qualitative and quantitative reviews that may have different epistemological underpinnings, which is necessary to answer our research questions. We will take a partly deductive approach to answering our research questions. We expect to draw on reviews, including but not necessarily limited to systematic reviews of effectiveness. From these studies, we will extract data on programme impacts, and on differential impacts on gender and age sub‐groups. In this way, our synthesis of data from reviews of quantitative studies will have much in common with current deductive approaches to the narrative synthesis of quantitative findings. Answering research question 2 entails in extracting data iteratively on factors (i.e. structural and individual‐level drivers and moderators) that may influence the impacts of social protection programmes on gender equality outcomes, as represented in the GRASSP Conceptual Framework. Similarly, for research question 3, we will build upon the typology of implementation and design issues considered in the framework. In this iterative synthesis, the results need to be organised so that patterns in findings from design and implementation of interventions can be identified across reviews (Popay et al., 2006). Based upon previous systematic reviews (e.g. Snilstveit et al., 2015) and on our own scoping exercise, evidence to answer these questions is likely to come from both qualitative and quantitative data. It may come from qualitative evidence on perceptions of intervention success or from quantitative data on implementation research.

##### 3.3.10.3 Mapping and interpretation

The main concepts for interventions, outcomes, structural and individual‐level drivers, moderators and implementation and design issues have been identified in the GRASSP Conceptual Framework, and these will be supplemented with additional themes emerging from the included reviews (Snilstveit et al., 2012). Findings will be presented following the components of the framework as a narrative interpretation as reported in the included systematic reviews, structured across the intervention areas or categories (i.e. social assistance, social insurance, labour market programmes and social care). Any frameworks or theories presented by the authors will also be considered. Any additional considerations regarding the setting where the intervention is implemented (e.g., humanitarian rural) will be noted as possible determinants of results. When reviews offer discordant results, findings will be presented along with a discussion on potential reasons for differing results. As previously described, we will report the pooled effect size of outcomes of meta‐analysis as well as subgroup analysis reporting differential impacts for gender or age groups in tables to be presented as Supplementary Information. No statistical analysis is proposed.

#### 3.3.11 Subgroup analysis and investigation of heterogeneity

The relationships or subgroup analysis to be explored as part of Step 2 of framework synthesis will include exploring different outcomes across gender and age groups (e.g., women and men, adolescent girls and boys, older adults) in order to explore differences in outcomes as well as what factors explain any identified patterns. Such findings will be reported as hypothesis generating rather than hypothesis testing.

## AUTHOR CONTRIBUTIONS

Camila Perera (C. P.), Shivit Bakrania (S. B.), Alessandra Ipince (A. I.), and Oluwaseun Ireti Obasola (O. I. O.) are evidence synthesis consultants at UNICEF's Office of Research and have led and/or co‐authored systematic reviews and Evidence Gap Maps in a wide range of interventions (e.g., child protection and well‐being, maternal and child health and psychological interventions). In addition, Camila Perera has experience in systematic review methodology, including statistical interpretation and analyses. Shivit Bakrania is the Knowledge Management Specialist at UNICEF's Office of Research and has experience as the Principal Investigator of high‐profile evidence gap map and evidence synthesis projects for DFID and UNICEF. Oluwaseun Ireti Obasola is an experienced information specialist and digital librarian. Zahrah Nesbitt‐Ahmed (Z. N. A.) is the Gender and Development Manager at UNICEF's Office of Research, manages the GRASSP programme and provides thematic and subject‐matter expertise. She has extensive experience in managing, researching and advising organisations in the areas of women's rights, gender equality and social protection. Dominic Richardson leads Social Policy and Economic Analysis at UNICEF, Office of Research—Innocenti, provides social protection expertise to this systematic review and has been involved in the conception, design and co‐ordination of the review.

- Content: C. P., S. B., A. I., O. O., Z. N. A., D. M.
- Systematic review methods: C. P., S. B., A. I., O. O.
- Statistical analysis: C. P., S. B.
- Information retrieval: C. P., A. I., O. O., S. B.

## PLANS FOR UPDATING THIS REVIEW

Systematic reviews of reviews are generally updated between 3 and 5 years depending on the need of an update (availability of new reviews). Regular updates are also subject to availability of funding. If funding is available, UNICEF Office of Research—Innocenti takes responsibility for updating the review.

## SOURCES OF SUPPORT

### External sources

- Foreign, Commonwealth & Development Office (FCDO), UK

This systematic review is part of a research programme investigating gender‐responsive and age‐sensitive social protection systems to enhance gender equality outcomes in low and middle‐income settings. The 5‐year programme (2018–2023) is led by UNICEF Office of Research—Innocenti and funded by the UK's Foreign, Commonwealth & Development Office (FCDO) and other partners. The systematic review is part of the first stream of the project and will inform future implementation within the programme.

## APPENDIX A ACADEMIC SEARCH COMPLETE

Name of database: Academic Search Complete

Date of search: 23/02/2021

Number of results: 370

**Table:**

#	Query	Results
S23	(S12 and S15 and (S16 or S17) and S22)	**370**
S22	S18 or S19 or S20 or S21	465,967
S21	**TI** (((systematic* OR synthes*) N3 (research OR evaluation* OR finding* OR thematic* OR report OR descriptive OR explanatory OR narrative OR meta* OR review* OR data OR literature OR studies OR evidence OR map OR quantitative OR study OR studies OR paper OR impact OR impacts OR effect* OR compar*))) OR **AB** (((systematic* OR synthes*) N3 (research OR evaluation* OR finding* OR thematic* OR report OR descriptive OR explanatory OR narrative OR meta* OR review* OR data OR literature OR studies OR evidence OR map OR quantitative OR study OR studies OR paper OR impact OR impacts OR effect* OR compar*))) OR **SU** (((systematic* OR synthes*) N3 (research OR evaluation* OR finding* OR thematic* OR report OR descriptive OR explanatory OR narrative OR meta* OR review* OR data OR literature OR studies OR evidence OR map OR quantitative OR study OR studies OR paper OR impact OR impacts OR effect* OR compar*)))	285,362
S20	**TI** ((("meta regression" OR "meta synth*" OR "meta‐synth*" OR "meta analy*" OR "metaanaly*" OR "meta‐analy*" OR "metanaly*" OR "metaregression" OR "meta‐regression" OR "methodologic* overview" OR "pool* analys*" OR "pool* data" OR "quantitative* overview" OR "research integration"))) OR **AB** ((("meta regression" OR "meta synth*" OR "meta‐synth*" OR "meta analy*" OR "metaanaly*" OR "meta‐analy*" OR "metanaly*" OR "metaregression" OR "meta‐regression" OR "methodologic* overview" OR "pool* analys*" OR "pool* data" OR "quantitative* overview" OR "research integration"))) OR **SU** ((("meta regression" OR "meta synth*" OR "meta‐synth*" OR "meta analy*" OR "metaanaly*" OR "meta‐analy*" OR "metanaly*" OR "metaregression" OR "meta‐regression" OR "methodologic* overview" OR "pool* analys*" OR "pool* data" OR "quantitative* overview" OR "research integration")))	115,753
S19	**TI** ((review N3 (effectiveness OR effects OR systemat* OR synth* OR integrat* OR map* OR methodologic* OR quantitative OR evidence OR literature)) OR **AB** ((review N3 (effectiveness OR effects OR systemat* OR synth* OR integrat* OR map* OR methodologic* OR quantitative OR evidence OR literature)) OR **SU** ((review N3 (effectiveness OR effects OR systemat* OR synth* OR integrat* OR map* OR methodologic* OR quantitative OR evidence OR literature))	252,205
S18	**TI** (("literature search" OR "database search" OR "bibliographic* search" OR comprehensive search" OR “extensive search" OR "exhaustive search" OR "purposive search" OR "representative search" OR "systemat* search") OR **AB** (("literature search" OR "database search" OR "bibliographic* search" OR comprehensive search" OR “extensive search" OR "exhaustive search" OR "purposive search" OR "representative search" OR "systemat* search") OR **SU** (("literature search" OR "database search" OR "bibliographic* search" OR comprehensive search" OR “extensive search" OR "exhaustive search" OR "purposive search" OR "representative search" OR "systemat* search")	22,531
S17	**TI** ((femini* OR masculi* OR female*OR male* OR gender* OR "social equality" OR "social equity" OR empower* OR "equal opportunit*" OR egalitarian*) OR **AB** ((femini* OR masculi* OR female*OR male* OR gender* OR "social equality" OR "social equity" OR empower* OR "equal opportunit*" OR egalitarian*) OR **SU** ((femini* OR masculi* OR female*OR male* OR gender* OR "social equality" OR "social equity" OR empower* OR "equal opportunit*" OR egalitarian*)	320,074
S16	**TI** (women OR "women's right" OR "young women" OR woman* OR adolescent* OR girl* OR schoolgirl* OR "school girl*" OR female* OR man OR men OR "young men" OR boy* OR schoolboy* OR "School boy*" OR teen* OR youth* OR young* OR mother* OR father* OR parent* OR caregiver) OR **AB** (women OR "women's right" OR "young women" OR woman* OR adolescent* OR girl* OR schoolgirl* OR "school girl*" OR female* OR man OR men OR "young men" OR boy* OR schoolboy* OR "School boy*" OR teen* OR youth* OR young* OR mother* OR father* OR parent* OR caregiver) OR **SU** (women OR "women's right" OR "young women" OR woman* OR adolescent* OR girl* OR schoolgirl* OR "school girl*" OR female* OR man OR men OR "young men" OR boy* OR schoolboy* OR "School boy*" OR teen* OR youth* OR young* OR mother* OR father* OR parent* OR caregiver)	2,087,976
S15	S13 OR S14	170,572
S14	**TI** ("Social assistance" OR "Social Pension*" OR "Public Pension" OR "Pension*" OR "In‐kind Transfer*" OR "Conditional Cash Transfer" OR "Unconditional Cash Transfer" "Cash Plus Program*" OR "Social Transfer" OR "Financial Transfer" OR "Monetary Transfer" OR "Financial Incentive" OR "Economic Asset" OR "Financial Protection" OR "Resource Transfer" OR "Childcare Cash Benefits" OR "Birth Grant" OR "Paternity Benefit" OR "Maternity Benefit" OR "Parental Leave" OR "Paternity Leave" OR "Maternity Leave" OR " Birth Payment" OR "Family Support" OR "Income Maintenance" OR "Income Support" OR "Universal Basic Income" OR "minimum income guarantee" OR "Fee waiver*" OR "Food Voucher" OR "School Voucher*" OR "School Feeding" OR "Unemployment Benefit*" OR Housing Subsidy " or " Housing Subsidies " or " Public Housing " or " Housing for the Elderly " or " Disability Grant* " or " Disability Support " or " Tax Exemption " or " Old Age Assistance " or " Old Age Pension " or " Lunch Program* " or " Breakfast Program* " or " Food Security Program* " or " Food Stamp " or " Sick Leave " or " Disability Leave " or " Compensatory Support " or " Child Program* " or Nutritional Assistance" OR "Nutritional Support" OR "Social Protection*" OR "Social Policy" OR "Social Policies" OR "Policy, Social" OR "Policies, Social" OR "Protection, Social" OR Welfare, Social " or " Infant Welfare " or " Child Welfare " " Maternal Welfare " or " Welfare, Maternal " or " Unemployment Insurance " or " Insurance, Social " or " Insurance, Health " or " Medical Assistance " or " Medicaid " or " School Health Service* " or " School Health Program* " or " Ancillary School Service* " or " Security, Social " or Medicare or or " Household Insurance " or " Homemaker Services " or " Benefit, Retirement " or " Benefits, Retirement " or " Retirement Pension " or Social Insurance" OR "Death Benefit" OR "Annuities" OR "Social Service*" OR "Humanitarian Assistance" OR "Health Insurance" OR "National Insurance" OR "Micro Health Insurance" OR "Community‐based Health Insurance*" OR "Health Benefit" OR "Health Subsidies" OR " Insurance Benefit" OR "Benefit System" OR "Social Security" OR "Social Welfare" OR "Social grant" OR "Non‐contributory" OR "Contributory" OR "Cash Transfer*" OR "Social Safety Net") OR **AB** ("Social assistance" OR "Social Pension*" OR "Public Pension" OR "Pension*" OR "In‐kind Transfer*" OR "Conditional Cash Transfer" OR "Unconditional Cash Transfer" "Cash Plus Program*" OR "Social Transfer" OR "Financial Transfer" OR "Monetary Transfer" OR "Financial Incentive" OR "Economic Asset" OR "Financial Protection" OR "Resource Transfer" OR "Childcare Cash Benefits" OR "Birth Grant" OR "Paternity Benefit" OR "Maternity Benefit" OR "Parental Leave" OR "Paternity Leave" OR "Maternity Leave" OR " Birth Payment" OR "Family Support" OR "Income Maintenance" OR "Income Support" OR "Universal Basic Income" OR "minimum income guarantee" OR "Fee waiver*" OR "Food Voucher" OR "School Voucher*" OR "School Feeding" OR "Unemployment Benefit*" OR Housing Subsidy " or " Housing Subsidies " or " Public Housing " or " Housing for the Elderly " or " Disability Grant* " or " Disability Support " or " Tax Exemption " or " Old Age Assistance " or " Old Age Pension " or " Lunch Program* " or " Breakfast Program* " or " Food Security Program* " or " Food Stamp " or " Sick Leave " or " Disability Leave " or " Compensatory Support " or " Child Program* " or Nutritional Assistance" OR "Nutritional Support" OR "Social Protection*" OR "Social Policy" OR "Social Policies" OR "Policy, Social" OR "Policies, Social" OR "Protection, Social" OR Welfare, Social " or " Infant Welfare " or " Child Welfare " " Maternal Welfare " or " Welfare, Maternal " or " Unemployment Insurance " or " Insurance, Social " or " Insurance, Health " or " Medical Assistance " or " Medicaid " or " School Health Service* " or " School Health Program* " or " Ancillary School Service* " or " Security, Social " or Medicare or or " Household Insurance " or " Homemaker Services " or " Benefit, Retirement " or " Benefits, Retirement " or " Retirement Pension " or Social Insurance" OR "Death Benefit" OR "Annuities" OR "Social Service*" OR "Humanitarian Assistance" OR "Health Insurance" OR "National Insurance" OR "Micro Health Insurance" OR "Community‐based Health Insurance*" OR "Health Benefit" OR "Health Subsidies" OR " Insurance Benefit" OR "Benefit System" OR "Social Security" OR "Social Welfare" OR "Social grant" OR "Non‐contributory" OR "Contributory" OR "Cash Transfer*" OR "Social Safety Net") OR **SU** ("Social assistance" OR "Social Pension*" OR "Public Pension" OR "Pension*" OR "In‐kind Transfer*" OR "Conditional Cash Transfer" OR "Unconditional Cash Transfer" "Cash Plus Program*" OR "Social Transfer" OR "Financial Transfer" OR "Monetary Transfer" OR "Financial Incentive" OR "Economic Asset" OR "Financial Protection" OR "Resource Transfer" OR "Childcare Cash Benefits" OR "Birth Grant" OR "Paternity Benefit" OR "Maternity Benefit" OR "Parental Leave" OR "Paternity Leave" OR "Maternity Leave" OR " Birth Payment" OR "Family Support" OR "Income Maintenance" OR "Income Support" OR "Universal Basic Income" OR "minimum income guarantee" OR "Fee waiver*" OR "Food Voucher" OR "School Voucher*" OR "School Feeding" OR "Unemployment Benefit*" OR Housing Subsidy " or " Housing Subsidies " or " Public Housing " or " Housing for the Elderly " or " Disability Grant* " or " Disability Support " or " Tax Exemption " or " Old Age Assistance " or " Old Age Pension " or " Lunch Program* " or " Breakfast Program* " or " Food Security Program* " or " Food Stamp " or " Sick Leave " or " Disability Leave " or " Compensatory Support " or " Child Program* " or Nutritional Assistance" OR "Nutritional Support" OR "Social Protection*" OR "Social Policy" OR "Social Policies" OR "Policy, Social" OR "Policies, Social" OR "Protection, Social" OR Welfare, Social " or " Infant Welfare " or " Child Welfare " " Maternal Welfare " or " Welfare, Maternal " or " Unemployment Insurance " or " Insurance, Social " or " Insurance, Health " or " Medical Assistance " or " Medicaid " or " School Health Service* " or " School Health Program* " or " Ancillary School Service* " or " Security, Social " or Medicare or or " Household Insurance " or " Homemaker Services " or " Benefit, Retirement " or " Benefits, Retirement " or " Retirement Pension " or Social Insurance" OR "Death Benefit" OR "Annuities" OR "Social Service*" OR "Humanitarian Assistance" OR "Health Insurance" OR "National Insurance" OR "Micro Health Insurance" OR "Community‐based Health Insurance*" OR "Health Benefit" OR "Health Subsidies" OR " Insurance Benefit" OR "Benefit System" OR "Social Security" OR "Social Welfare" OR "Social grant" OR "Non‐contributory" OR "Contributory" OR "Cash Transfer*" OR "Social Safety Net")	121,839
S13	**TI** ("Labour Market Policy" OR "Labour Market Policies" OR "Activation Policies" OR "Active Labour Market Policies" OR "Labour Market Program*" OR "Labour Market Reform*" OR "Labour Standard*" OR "Labour Supply Program*" OR "Labor Market Policy" OR "Labor market Policies" OR "Labor Market Program*" OR "Labor Market Reform*" OR "Labor Supply Program*" OR "Labor Standard*" OR "Livelihood Support" OR "Livelihood Program*" OR "Livelihood Project" OR "Livelihood Empowerment" OR "Poverty Reduction Program*" OR "Poverty Alleviation Program*" OR "Skills Development" OR "Skill Development" OR "Vocational Training" OR "Job Training" OR "Training Support" OR "Entrepreneurship Program*" OR "Entrepreneurship Promotion" OR "Employment Program*" OR "Employment Scheme*" OR "Employment Service*" OR "Employee Assistance Programs" OR " Youth Employment Program*" OR "Wage Subsidy" OR "Wage Subsidies" OR "Hiring Subsidy" OR "Hiring Subsidies " OR "Public Works" OR "Employment, Supported" OR "Assistance Program*, Employee" OR " Integrated Program*" OR "Community Integration" OR "Social Care*" OR "Child Support" OR "Child Grant" OR Daycare OR "Centre‐based childcare" OR "Center‐based childcare" OR "After School Clubs" OR "Child Care Service*" OR "Care for Children" OR "Parenting Education" OR "Education, Parenting" OR "Care for Children" OR Care for the Elderly OR "Home Care Services" OR "Home Health Nursing" OR "Foster Home Care" OR "Nurse Family Partnership" OR "Aid to families with dependent children" OR "Adult Protective Services" OR "Aid to the Totally Disabled" OR "Aid to Visually Impaired" OR "IPV Interventions" OR "Community‐based Program*" OR "Community Care" "Community‐based Intervention*" OR "Community‐based Health Insurance*" OR "Community‐based Project*" OR "Community Services" OR "Services, Community" OR "Community Service" OR "Service, Community") OR **AB** ("Labour Market Policy" OR "Labour Market Policies" OR "Activation Policies" OR "Active Labour Market Policies" OR "Labour Market Program*" OR "Labour Market Reform*" OR "Labour Standard*" OR "Labour Supply Program*" OR "Labor Market Policy" OR "Labor market Policies" OR "Labor Market Program*" OR "Labor Market Reform*" OR "Labor Supply Program*" OR "Labor Standard*" OR "Livelihood Support" OR "Livelihood Program*" OR "Livelihood Project" OR "Livelihood Empowerment" OR "Poverty Reduction Program*" OR "Poverty Alleviation Program*" OR "Skills Development" OR "Skill Development" OR "Vocational Training" OR "Job Training" OR "Training Support" OR "Entrepreneurship Program*" OR "Entrepreneurship Promotion" OR "Employment Program*" OR "Employment Scheme*" OR "Employment Service*" OR "Employee Assistance Programs" OR " Youth Employment Program*" OR "Wage Subsidy" OR "Wage Subsidies" OR "Hiring Subsidy" OR "Hiring Subsidies " OR "Public Works" OR "Employment, Supported" OR "Assistance Program*, Employee" OR " Integrated Program*" OR "Community Integration" OR "Social Care*" OR "Child Support" OR "Child Grant" OR Daycare OR "Centre‐based childcare" OR "Center‐based childcare" OR "After School Clubs" OR "Child Care Service*" OR "Care for Children" OR "Parenting Education" OR "Education, Parenting" OR "Care for Children" OR Care for the Elderly OR "Home Care Services" OR "Home Health Nursing" OR "Foster Home Care" OR "Nurse Family Partnership" OR "Aid to families with dependent children" OR "Adult Protective Services" OR "Aid to the Totally Disabled" OR "Aid to Visually Impaired" OR "IPV Interventions" OR "Community‐based Program*" OR "Community Care" "Community‐based Intervention*" OR "Community‐based Health Insurance*" OR "Community‐based Project*" OR "Community Services" OR "Services, Community" OR "Community Service" OR "Service, Community") OR **SU** ("Labour Market Policy" OR "Labour Market Policies" OR "Activation Policies" OR "Active Labour Market Policies" OR "Labour Market Program*" OR "Labour Market Reform*" OR "Labour Standard*" OR "Labour Supply Program*" OR "Labor Market Policy" OR "Labor market Policies" OR "Labor Market Program*" OR "Labor Market Reform*" OR "Labor Supply Program*" OR "Labor Standard*" OR "Livelihood Support" OR "Livelihood Program*" OR "Livelihood Project" OR "Livelihood Empowerment" OR "Poverty Reduction Program*" OR "Poverty Alleviation Program*" OR "Skills Development" OR "Skill Development" OR "Vocational Training" OR "Job Training" OR "Training Support" OR "Entrepreneurship Program*" OR "Entrepreneurship Promotion" OR "Employment Program*" OR "Employment Scheme*" OR "Employment Service*" OR "Employee Assistance Programs" OR " Youth Employment Program*" OR "Wage Subsidy" OR "Wage Subsidies" OR "Hiring Subsidy" OR "Hiring Subsidies " OR "Public Works" OR "Employment, Supported" OR "Assistance Program*, Employee" OR " Integrated Program*" OR "Community Integration" OR "Social Care*" OR "Child Support" OR "Child Grant" OR Daycare OR "Centre‐based childcare" OR "Center‐based childcare" OR "After School Clubs" OR "Child Care Service*" OR "Care for Children" OR "Parenting Education" OR "Education, Parenting" OR "Care for Children" OR Care for the Elderly OR "Home Care Services" OR "Home Health Nursing" OR "Foster Home Care" OR "Nurse Family Partnership" OR "Aid to families with dependent children" OR "Adult Protective Services" OR "Aid to the Totally Disabled" OR "Aid to Visually Impaired" OR "IPV Interventions" OR "Community‐based Program*" OR "Community Care" "Community‐based Intervention*" OR "Community‐based Health Insurance*" OR "Community‐based Project*" OR "Community Services" OR "Services, Community" OR "Community Service" OR "Service, Community")	53,546
S12	S1 OR S2 OR S3 OR S4 OR S5 OR S6 OR S7 OR S8 OR S9 OR S10 OR S11	1,919,352
S11	**TI** (("transitional country" OR "transitional countries")) OR **AB** (("transitional country" OR "transitional countries")) OR **SU** (("transitional country" OR "transitional countries"))	147
S10	TI ((emerging N0 (economy or economies or nation*))) OR **AB** ((emerging N0 (economy or economies or nation*))) OR **SU** ((emerging N0 (economy or economies or nation*)))	2,314
S9	**TI** (((low* N1 (gdp OR gnp OR "gross domestic" OR "gross national"))) OR **AB** (((low* N1 (gdp OR gnp OR "gross domestic" OR "gross national"))) OR **SU** (((low* N1 (gdp OR gnp OR "gross domestic" OR "gross national")))	305
S8	**TI** (low N3 middle N3 countr*) OR **AB** (low N3 middle N3 countr*) OR **SU** (low N3 middle N3 countr*)	11,446
S7	**TI** (((developing OR (less* N1 developed) OR "under developed" OR "middle income" or (low* N1 income)) N1 (economy or economies))) OR **AB** (((developing OR (less* N1 developed) OR "under developed" OR "middle income" or (low* N1 income)) N1 (economy or economies))) OR **SU** (((developing OR (less* N1 developed) OR "under developed" OR "middle income" or (low* N1 income)) N1 (economy or economies)))	1826
S6	**TI** (((developing OR (less* N1 developed) OR "under developed" OR underdeveloped OR "under served" OR underserved OR deprived OR poor* OR "middle income" or (low* N1 income)) N1 (countr* or nation* or population* or world))) OR **AB** (((developing OR (less* N1 developed) OR "under developed" OR underdeveloped OR "under served" OR underserved OR deprived OR poor* OR "middle income" or (low* N1 income)) N1 (countr* or nation* or population* or world))) OR **SU** (((developing OR (less* N1 developed) OR "under developed" OR underdeveloped OR "under served" OR underserved OR deprived OR poor* OR "middle income" or (low* N1 income)) N1 (countr* or nation* or population* or world)))	76,957
S5	**TI** (Africa OR Asia OR Caribbean OR "West Indies" OR "South America" OR "Latin America" OR "Central America" OR "Middle East" OR "global south") OR **AB** (Africa OR Asia OR Caribbean OR "West Indies" OR "South America" OR "Latin America" OR "Central America" OR "Middle East" OR "global south") OR **SU** (Africa OR Asia OR Caribbean OR "West Indies" OR "South America" OR "Latin America" OR "Central America" OR "Middle East" OR "global south")	296,242
S4	**TI** ("west indies" OR "indian ocean islands" OR caribbean OR "central america" OR "latin america" OR "south america" OR magreb OR maghrib OR "central asia" OR "north asia" OR "northern asia" OR "southeastern asia" OR "south eastern asia" OR "southeast asia" OR "south east asia" OR "western asia" OR "east europe" OR "eastern europe") OR **AB** ("west indies" OR "indian ocean islands" OR caribbean OR "central america" OR "latin america" OR "south america" OR magreb OR maghrib OR "central asia" OR "north asia" OR "northern asia" OR "southeastern asia" OR "south eastern asia" OR "southeast asia" OR "south east asia" OR "western asia" OR "east europe" OR "eastern europe") OR **SU** ("west indies" OR "indian ocean islands" OR caribbean OR "central america" OR "latin america" OR "south america" OR magreb OR maghrib OR "central asia" OR "north asia" OR "northern asia" OR "southeastern asia" OR "south eastern asia" OR "southeast asia" OR "south east asia" OR "western asia" OR "east europe" OR "eastern europe")	90,454
S3	**SU** afghanistan OR albania OR algeria OR "american samoa" OR angola OR antigua OR barbuda OR argentina OR armenia OR armenian OR aruba OR azerbaijan OR bahrain OR bangladesh OR barbados OR belarus OR byelarus OR belorussia OR byelorussian OR belize OR "british honduras" OR benin OR dahomey OR bhutan OR bolivia OR bosnia OR herzegovina OR Botswana OR bechuanaland OR brazil OR brasil OR bulgaria OR "burkina faso" OR "burkina fasso" OR "upper volta" OR burundi OR urundi OR "cabo verde" OR "cape verde" OR cambodia OR kampuchea OR "khmer republic" OR cameroon OR cameron OR cameroun OR "central african republic" OR "ubangi shari" OR chad OR chile OR china OR colombia OR comoros OR "comoro islands" OR mayotte OR congo OR zaire OR "costa rica" OR "cote d'ivoire" OR "cote d'ivoire" OR "cote d'ivoire" OR "ivory coast" OR croatia OR cuba OR cyprus OR "czech republic" OR czechoslovakia OR djibouti OR "french somaliland" OR dominica OR "dominican republic" OR ecuador OR egypt OR "united arab republic" OR "el salvador" OR "equatorial guinea" OR "spanish guinea" OR eritrea OR estonia OR eswatini OR swaziland OR ethiopia OR fiji OR gabon OR "gabonese republic" OR gambia OR georgia OR Georgian OR ghana OR "gold coast" OR gibraltar OR greece OR grenada OR guam OR guatemala OR guinea OR guyana OR guiana OR haiti OR hispaniola OR honduras OR hungary OR india OR indonesia OR timor OR iran OR iraq OR "isle of man" OR jamaica OR jordan OR kazakhstan OR kazakh OR kenya OR korea OR kosovo OR kyrgyzstan OR kirghizia OR kirgizstan OR "kyrgyz republic" OR kirghiz OR laos OR "lao pdr" OR "lao people's democratic republic" OR latvia OR lebanon OR lesotho OR basutoland OR liberia OR libya OR "libyan arab jamahiriya" OR lithuania OR macau OR macao OR macedonia OR madagascar OR "malagasy republic" OR malawi OR nyasaland OR malaysia OR maldives OR "indian ocean" OR mali OR malta OR micronesia OR kiribati OR "marshall islands" OR nauru OR "northern mariana islands" OR palau OR tuvalu OR mauritania OR mauritius OR mexico OR moldova OR moldovian OR mongolia OR montenegro OR morocco OR ifni OR mozambique OR "portuguese east africa" OR myanmar OR burma OR namibia OR nepal OR "netherlands antilles" OR nicaragua OR niger OR nigeria OR oman OR muscat OR pakistan OR panama OR "papua new guinea" OR paraguay OR peru OR philippines OR philipines OR phillipines OR phillippines OR poland OR "polish people's republic" OR portugal OR "portuguese republic" OR "puerto rico" OR romania OR russia OR "russian federation" OR ussr OR "soviet union" OR "union of soviet socialist republics" OR rwanda OR ruanda OR samoa OR "pacific islands" OR polynesia OR "samoan islands" OR "sao tome" and principe OR "saudi arabia" OR senegal OR serbia OR seychelles OR "sierra leone" OR slovakia OR "slovak republic" OR slovenia OR melanesia OR "solomon island" OR "solomon islands" OR "norfolk island" OR somalia OR "south africa" OR "south sudan" OR "sri lanka" OR ceylon OR "saint kitts" OR nevis OR "st kitts" OR nevis OR "saint lucia" OR "st lucia" OR "saint vincent" OR "st vincent" OR grenadines OR sudan OR suriname OR surinam OR syria OR "syrian OR muscat OR pakistan OR panama OR "papua new guinea" OR paraguay OR peru OR philippines OR philipines OR phillipines OR phillippines OR poland OR "polish people's republic" OR portugal OR "portuguese republic" OR "puerto rico" OR romania OR russia OR "russian federation" OR ussr OR "soviet union" OR "union of soviet socialist republics" OR rwanda OR ruanda OR samoa OR "pacific islands" OR polynesia OR "samoan islands" OR "sao tome" and principe OR "saudi arabia" OR senegal OR serbia OR seychelles OR "sierra leone" OR slovakia OR "slovak republic" OR slovenia OR melanesia OR "solomon island" OR "solomon islands" OR "norfolk island" OR somalia OR "south africa" OR "south sudan" OR "sri lanka" OR ceylon OR "saint kitts" OR nevis OR "st kitts" OR nevis OR "saint lucia" OR "st lucia" OR "saint vincent" OR "st vincent" OR grenadines OR sudan OR suriname OR surinam OR syria OR "syrian arab republic" OR tajikistan OR tadjikistan OR tadzhikistan OR tadzhik OR tanzania OR Tanganyika OR thailand OR siam OR "timor leste" OR "east timor" OR togo OR "togolese republic" OR tonga OR trinidad OR tobago OR tunisia OR turkey OR turkmenistan OR turkmen OR uganda OR ukraine OR uruguay OR uzbekistan OR uzbek OR vanuatu OR "new hebrides" OR venezuela OR vietnam OR "viet name" OR "west bank" OR gaza OR palestine OR yemen OR yugoslavia OR zambia OR zimbabwe OR "northern rhodesia"	1,054,299
S2	**AB** afghanistan OR albania OR algeria OR "american samoa" OR angola OR antigua OR barbuda OR argentina OR armenia OR armenian OR aruba OR azerbaijan OR bahrain OR bangladesh OR barbados OR belarus OR byelarus OR belorussia OR byelorussian OR belize OR "british honduras" OR benin OR dahomey OR bhutan OR bolivia OR bosnia OR herzegovina OR Botswana OR bechuanaland OR brazil OR brasil OR bulgaria OR "burkina faso" OR "burkina fasso" OR "upper volta" OR burundi OR urundi OR "cabo verde" OR "cape verde" OR cambodia OR kampuchea OR "khmer republic" OR cameroon OR cameron OR cameroun OR "central african republic" OR "ubangi shari" OR chad OR chile OR china OR colombia OR comoros OR "comoro islands" OR mayotte OR congo OR zaire OR "costa rica" OR "cote d'ivoire" OR "cote d'ivoire" OR "cote d'ivoire" OR "ivory coast" OR croatia OR cuba OR cyprus OR "czech republic" OR czechoslovakia OR djibouti OR "french somaliland" OR dominica OR "dominican republic" OR ecuador OR egypt OR "united arab republic" OR "el salvador" OR "equatorial guinea" OR "spanish guinea" OR eritrea OR estonia OR eswatini OR swaziland OR ethiopia OR fiji OR gabon OR "gabonese republic" OR gambia OR georgia OR Georgian OR ghana OR "gold coast" OR gibraltar OR greece OR grenada OR guam OR guatemala OR guinea OR guyana OR guiana OR haiti OR hispaniola OR honduras OR hungary OR india OR indonesia OR timor OR iran OR iraq OR "isle of man" OR jamaica OR jordan OR kazakhstan OR kazakh OR kenya OR korea OR kosovo OR kyrgyzstan OR kirghizia OR kirgizstan OR "kyrgyz republic" OR kirghiz OR laos OR "lao pdr" OR "lao people's democratic republic" OR latvia OR lebanon OR lesotho OR basutoland OR liberia OR libya OR "libyan arab jamahiriya" OR lithuania OR macau OR macao OR macedonia OR madagascar OR "malagasy republic" OR malawi OR nyasaland OR malaysia OR maldives OR "indian ocean" OR mali OR malta OR micronesia OR kiribati OR "marshall islands" OR nauru OR "northern mariana islands" OR palau OR tuvalu OR mauritania OR mauritius OR mexico OR moldova OR moldovian OR mongolia OR montenegro OR morocco OR ifni OR mozambique OR "portuguese east africa" OR myanmar OR burma OR namibia OR nepal OR "netherlands antilles" OR nicaragua OR niger OR nigeria OR oman OR muscat OR pakistan OR panama OR "papua new guinea" OR paraguay OR peru OR philippines OR philipines OR phillipines OR phillippines OR poland OR "polish people's republic" OR portugal OR "portuguese republic" OR "puerto rico" OR romania OR russia OR "russian federation" OR ussr OR "soviet union" OR "union of soviet socialist republics" OR rwanda OR ruanda OR samoa OR "pacific islands" OR polynesia OR "samoan islands" OR "sao tome" and principe OR "saudi arabia" OR senegal OR serbia OR seychelles OR "sierra leone" OR slovakia OR "slovak republic" OR slovenia OR melanesia OR "solomon island" OR "solomon islands" OR "norfolk island" OR somalia OR "south africa" OR "south sudan" OR "sri lanka" OR ceylon OR "saint kitts" OR nevis OR "st kitts" OR nevis OR "saint lucia" OR "st lucia" OR "saint vincent" OR "st vincent" OR grenadines OR sudan OR suriname OR surinam OR syria OR "syrian OR muscat OR pakistan OR panama OR "papua new guinea" OR paraguay OR peru OR philippines OR philipines OR phillipines OR phillippines OR poland OR "polish people's republic" OR portugal OR "portuguese republic" OR "puerto rico" OR romania OR russia OR "russian federation" OR ussr OR "soviet union" OR "union of soviet socialist republics" OR rwanda OR ruanda OR samoa OR "pacific islands" OR polynesia OR "samoan islands" OR "sao tome" and principe OR "saudi arabia" OR senegal OR serbia OR seychelles OR "sierra leone" OR slovakia OR "slovak republic" OR slovenia OR melanesia OR "solomon island" OR "solomon islands" OR "norfolk island" OR somalia OR "south africa" OR "south sudan" OR "sri lanka" OR ceylon OR "saint kitts" OR nevis OR "st kitts" OR nevis OR "saint lucia" OR "st lucia" OR "saint vincent" OR "st vincent" OR grenadines OR sudan OR suriname OR surinam OR syria OR "syrian arab republic" OR tajikistan OR tadjikistan OR tadzhikistan OR tadzhik OR tanzania OR Tanganyika OR thailand OR siam OR "timor leste" OR "east timor" OR togo OR "togolese republic" OR tonga OR trinidad OR tobago OR tunisia OR turkey OR turkmenistan OR turkmen OR uganda OR ukraine OR uruguay OR uzbekistan OR uzbek OR vanuatu OR "new hebrides" OR venezuela OR vietnam OR "viet name" OR "west bank" OR gaza OR palestine OR yemen OR yugoslavia OR zambia OR zimbabwe OR "northern rhodesia"	1,313,982
S1	**TI** afghanistan OR albania OR algeria OR "american samoa" OR angola OR antigua OR barbuda OR argentina OR armenia OR armenian OR aruba OR azerbaijan OR bahrain OR bangladesh OR barbados OR belarus OR byelarus OR belorussia OR byelorussian OR belize OR "british honduras" OR benin OR dahomey OR bhutan OR bolivia OR bosnia OR herzegovina OR Botswana OR bechuanaland OR brazil OR brasil OR bulgaria OR "burkina faso" OR "burkina fasso" OR "upper volta" OR burundi OR urundi OR "cabo verde" OR "cape verde" OR cambodia OR kampuchea OR "khmer republic" OR cameroon OR cameron OR cameroun OR "central african republic" OR "ubangi shari" OR chad OR chile OR china OR colombia OR comoros OR "comoro islands" OR mayotte OR congo OR zaire OR "costa rica" OR "cote d'ivoire" OR "cote d'ivoire" OR "cote d'ivoire" OR "ivory coast" OR croatia OR cuba OR cyprus OR "czech republic" OR czechoslovakia OR djibouti OR "french somaliland" OR dominica OR "dominican republic" OR ecuador OR egypt OR "united arab republic" OR "el salvador" OR "equatorial guinea" OR "spanish guinea" OR eritrea OR estonia OR eswatini OR swaziland OR ethiopia OR fiji OR gabon OR "gabonese republic" OR gambia OR georgia OR Georgian OR ghana OR "gold coast" OR gibraltar OR greece OR grenada OR guam OR guatemala OR guinea OR guyana OR guiana OR haiti OR hispaniola OR honduras OR hungary OR india OR indonesia OR timor OR iran OR iraq OR "isle of man" OR jamaica OR jordan OR kazakhstan OR kazakh OR kenya OR korea OR kosovo OR kyrgyzstan OR kirghizia OR kirgizstan OR "kyrgyz republic" OR kirghiz OR laos OR "lao pdr" OR "lao people's democratic republic" OR latvia OR lebanon OR lesotho OR basutoland OR liberia OR libya OR "libyan arab jamahiriya" OR lithuania OR macau OR macao OR macedonia OR madagascar OR "malagasy republic" OR malawi OR nyasaland OR malaysia OR maldives OR "indian ocean" OR mali OR malta OR micronesia OR kiribati OR "marshall islands" OR nauru OR "northern mariana islands" OR palau OR tuvalu OR mauritania OR mauritius OR mexico OR moldova OR moldovian OR mongolia OR montenegro OR morocco OR ifni OR mozambique OR "portuguese east africa" OR myanmar OR burma OR namibia OR nepal OR "netherlands antilles" OR nicaragua OR niger OR nigeria OR oman OR muscat OR pakistan OR panama OR "papua new guinea" OR paraguay OR peru OR philippines OR philipines OR phillipines OR phillippines OR poland OR "polish people's republic" OR portugal OR "portuguese republic" OR "puerto rico" OR romania OR russia OR "russian federation" OR ussr OR "soviet union" OR "union of soviet socialist republics" OR rwanda OR ruanda OR samoa OR "pacific islands" OR polynesia OR "samoan islands" OR "sao tome" and principe OR "saudi arabia" OR senegal OR serbia OR seychelles OR "sierra leone" OR slovakia OR "slovak republic" OR slovenia OR melanesia OR "solomon island" OR "solomon islands" OR "norfolk island" OR somalia OR "south africa" OR "south sudan" OR "sri lanka" OR ceylon OR "saint kitts" OR nevis OR "st kitts" OR nevis OR "saint lucia" OR "st lucia" OR "saint vincent" OR "st vincent" OR grenadines OR sudan OR suriname OR surinam OR syria OR "syrian OR muscat OR pakistan OR panama OR "papua new guinea" OR paraguay OR peru OR philippines OR philipines OR phillipines OR phillippines OR poland OR "polish people's republic" OR portugal OR "portuguese republic" OR "puerto rico" OR romania OR russia OR "russian federation" OR ussr OR "soviet union" OR "union of soviet socialist republics" OR rwanda OR ruanda OR samoa OR "pacific islands" OR polynesia OR "samoan islands" OR "sao tome" and principe OR "saudi arabia" OR senegal OR serbia OR seychelles OR "sierra leone" OR slovakia OR "slovak republic" OR slovenia OR melanesia OR "solomon island" OR "solomon islands" OR "norfolk island" OR somalia OR "south africa" OR "south sudan" OR "sri lanka" OR ceylon OR "saint kitts" OR nevis OR "st kitts" OR nevis OR "saint lucia" OR "st lucia" OR "saint vincent" OR "st vincent" OR grenadines OR sudan OR suriname OR surinam OR syria OR "syrian arab republic" OR tajikistan OR tadjikistan OR tadzhikistan OR tadzhik OR tanzania OR Tanganyika OR thailand OR siam OR "timor leste" OR "east timor" OR togo OR "togolese republic" OR tonga OR trinidad OR tobago OR tunisia OR turkey OR turkmenistan OR turkmen OR uganda OR ukraine OR uruguay OR uzbekistan OR uzbek OR vanuatu OR "new hebrides" OR venezuela OR vietnam OR "viet name" OR "west bank" OR gaza OR palestine OR yemen OR yugoslavia OR zambia OR zimbabwe OR "northern rhodesia"	728,972

## APPENDIX B AFRICA‐WIDE

Name of database: Africa‐Wide

Date of search: 25/02/2020

Number of results: 297

**Table:**

#	Query	Results
S26	S10 AND S15 AND S16 AND S25	297
S25	S17 OR S18 OR S19 OR S20 OR S21 OR S22 OR S23 OR S24	2,384,019
S24	(TI(afghanistan OR albania OR algeria OR "american samoa" OR angola OR antigua OR barbuda OR argentina OR armenia OR armenian OR aruba OR azerbaijan OR bahrain OR bangladesh OR barbados OR belarus OR byelarus OR belorussia OR byelorussian OR belize OR "british honduras" OR benin OR dahomey OR bhutan OR bolivia OR bosnia OR herzegovina OR Botswana OR bechuanaland OR brazil OR brasil OR bulgaria OR "burkina faso" OR "burkina fasso" OR "upper volta" OR burundi OR urundi OR "cabo verde" OR "cape verde" OR cambodia OR kampuchea OR "khmer republic" OR cameroon OR cameron OR cameroun OR "central african republic" OR "ubangi shari" OR chad OR chile OR china OR colombia OR comoros OR "comoro islands" OR mayotte OR congo OR zaire OR "costa rica" OR "cote d'ivoire" OR "cote d'ivoire" OR "cote d'ivoire" OR "ivory coast" OR croatia OR cuba OR cyprus OR "czech republic" OR czechoslovakia OR djibouti OR "french somaliland" OR dominica OR "dominican republic" OR ecuador OR egypt OR "united arab republic" OR "el salvador" OR "equatorial guinea" OR "spanish guinea" OR eritrea OR estonia OR eswatini OR swaziland OR ethiopia OR fiji OR gabon OR "gabonese republic" OR gambia OR georgia OR Georgian OR ghana OR "gold coast" OR gibraltar OR greece OR grenada OR guam OR guatemala OR guinea OR guyana OR guiana OR haiti OR hispaniola OR honduras OR hungary OR india OR indonesia OR timor OR iran OR iraq OR "isle of man" OR jamaica OR jordan OR kazakhstan OR kazakh OR kenya OR korea OR kosovo OR kyrgyzstan OR kirghizia OR kirgizstan OR "kyrgyz republic" OR kirghiz OR laos OR "lao pdr" OR "lao people's democratic republic" OR latvia OR lebanon OR lesotho OR basutoland OR liberia OR libya OR "libyan arab jamahiriya" OR lithuania OR macau OR macao OR macedonia OR madagascar OR "malagasy republic" OR malawi OR nyasaland OR malaysia OR maldives OR "indian ocean" OR mali OR malta OR micronesia OR kiribati OR "marshall islands" OR nauru OR "northern mariana islands" OR palau OR tuvalu OR mauritania OR mauritius OR mexico OR moldova OR moldovian OR mongolia OR montenegro OR morocco OR ifni OR mozambique OR "portuguese east africa" OR myanmar OR burma OR namibia OR nepal OR "netherlands antilles" OR nicaragua OR niger OR nigeria OR oman OR muscat OR pakistan OR panama OR "papua new guinea" OR paraguay OR peru OR philippines OR philipines OR phillipines OR phillippines OR poland OR "polish people's republic" OR portugal OR "portuguese republic" OR "puerto rico" OR romania OR russia OR "russian federation" OR ussr OR "soviet union" OR "union of soviet socialist republics" OR rwanda OR ruanda OR samoa OR "pacific islands" OR polynesia OR "samoan islands" OR "sao tome" and principe OR "saudi arabia" OR senegal OR serbia OR seychelles OR "sierra leone" OR slovakia OR "slovak republic" OR slovenia OR melanesia OR "solomon island" OR "solomon islands" OR "norfolk island" OR somalia OR "south africa" OR "south sudan" OR "sri lanka" OR ceylon OR "saint kitts" OR nevis OR "st kitts" OR nevis OR "saint lucia" OR "st lucia" OR "saint vincent" OR "st vincent" OR grenadines OR sudan OR suriname OR surinam OR syria OR "syrian OR muscat OR pakistan OR panama OR "papua new guinea" OR paraguay OR peru OR philippines OR philipines OR phillipines OR phillippines OR poland OR "polish people's republic" OR portugal OR "portuguese republic" OR "puerto rico" OR romania OR russia OR "russian federation" OR ussr OR "soviet union" OR "union of soviet socialist republics" OR rwanda OR ruanda OR samoa OR "pacific islands" OR polynesia OR "samoan islands" OR "sao tome" and principe OR "saudi arabia" OR senegal OR serbia OR seychelles OR "sierra leone" OR slovakia OR "slovak republic" OR slovenia OR melanesia OR "solomon island" OR "solomon islands" OR "norfolk island" OR somalia OR "south africa" OR "south sudan" OR "sri lanka" OR ceylon OR "saint kitts" OR nevis OR "st kitts" OR nevis OR "saint lucia" OR "st lucia" OR "saint vincent" OR "st vincent" OR grenadines OR sudan OR suriname OR surinam OR syria OR "syrian arab republic" OR tajikistan OR tadjikistan OR tadzhikistan OR tadzhik OR tanzania OR Tanganyika OR thailand OR siam OR "timor leste" OR "east timor" OR togo OR "togolese republic" OR tonga OR trinidad OR tobago OR tunisia OR turkey OR turkmenistan OR turkmen OR uganda OR ukraine OR uruguay OR uzbekistan OR uzbek OR vanuatu OR "new hebrides" OR venezuela OR vietnam OR "viet name" OR "west bank" OR gaza OR palestine OR yemen OR yugoslavia OR zambia OR zimbabwe OR "northern rhodesia") OR SU (afghanistan OR albania OR algeria OR "american samoa" OR angola OR antigua OR barbuda OR argentina OR armenia OR armenian OR aruba OR azerbaijan OR bahrain OR bangladesh OR barbados OR belarus OR byelarus OR belorussia OR byelorussian OR belize OR "british honduras" OR benin OR dahomey OR bhutan OR bolivia OR bosnia OR herzegovina OR Botswana OR bechuanaland OR brazil OR brasil OR bulgaria OR "burkina faso" OR "burkina fasso" OR "upper volta" OR burundi OR urundi OR "cabo verde" OR "cape verde" OR cambodia OR kampuchea OR "khmer republic" OR cameroon OR cameron OR cameroun OR "central african republic" OR "ubangi shari" OR chad OR chile OR china OR colombia OR comoros OR "comoro islands" OR mayotte OR congo OR zaire OR "costa rica" OR "cote d'ivoire" OR "cote d'ivoire" OR "cote d'ivoire" OR "ivory coast" OR croatia OR cuba OR cyprus OR "czech republic" OR czechoslovakia OR djibouti OR "french somaliland" OR dominica OR "dominican republic" OR ecuador OR egypt OR "united arab republic" OR "el salvador" OR "equatorial guinea" OR "spanish guinea" OR eritrea OR estonia OR eswatini OR swaziland OR ethiopia OR fiji OR gabon OR "gabonese republic" OR gambia OR georgia OR Georgian OR ghana OR "gold coast" OR gibraltar OR greece OR grenada OR guam OR guatemala OR guinea OR guyana OR guiana OR haiti OR hispaniola OR honduras OR hungary OR india OR indonesia OR timor OR iran OR iraq OR "isle of man" OR jamaica OR jordan OR kazakhstan OR kazakh OR kenya OR korea OR kosovo OR kyrgyzstan OR kirghizia OR kirgizstan OR "kyrgyz republic" OR kirghiz OR laos OR "lao pdr" OR "lao people's democratic republic" OR latvia OR lebanon OR lesotho OR basutoland OR liberia OR libya OR "libyan arab jamahiriya" OR lithuania OR macau OR macao OR macedonia OR madagascar OR "malagasy republic" OR malawi OR nyasaland OR malaysia OR maldives OR "indian ocean" OR mali OR malta OR micronesia OR kiribati OR "marshall islands" OR nauru OR "northern mariana islands" OR palau OR tuvalu OR mauritania OR mauritius OR mexico OR moldova OR moldovian OR mongolia OR montenegro OR morocco OR ifni OR mozambique OR "portuguese east africa" OR myanmar OR burma OR namibia OR nepal OR "netherlands antilles" OR nicaragua OR niger OR nigeria OR oman OR muscat OR pakistan OR panama OR "papua new guinea" OR paraguay OR peru OR philippines OR philipines OR phillipines OR phillippines OR poland OR "polish people's republic" OR portugal OR "portuguese republic" OR "puerto rico" OR romania OR russia OR "russian federation" OR ussr OR "soviet union" OR "union of soviet socialist republics" OR rwanda OR ruanda OR samoa OR "pacific islands" OR polynesia OR "samoan islands" OR "sao tome" and principe OR "saudi arabia" OR senegal OR serbia OR seychelles OR "sierra leone" OR slovakia OR "slovak republic" OR slovenia OR melanesia OR "solomon island" OR "solomon islands" OR "norfolk island" OR somalia OR "south africa" OR "south sudan" OR "sri lanka" OR ceylon OR "saint kitts" OR nevis OR "st kitts" OR nevis OR "saint lucia" OR "st lucia" OR "saint vincent" OR "st vincent" OR grenadines OR sudan OR suriname OR surinam OR syria OR "syrian OR muscat OR pakistan OR panama OR "papua new guinea" OR paraguay OR peru OR philippines OR philipines OR phillipines OR phillippines OR poland OR "polish people's republic" OR portugal OR "portuguese republic" OR "puerto rico" OR romania OR russia OR "russian federation" OR ussr OR "soviet union" OR "union of soviet socialist republics" OR rwanda OR ruanda OR samoa OR "pacific islands" OR polynesia OR "samoan islands" OR "sao tome" and principe OR "saudi arabia" OR senegal OR serbia OR seychelles OR "sierra leone" OR slovakia OR "slovak republic" OR slovenia OR melanesia OR "solomon island" OR "solomon islands" OR "norfolk island" OR somalia OR "south africa" OR "south sudan" OR "sri lanka" OR ceylon OR "saint kitts" OR nevis OR "st kitts" OR nevis OR "saint lucia" OR "st lucia" OR "saint vincent" OR "st vincent" OR grenadines OR sudan OR suriname OR surinam OR syria OR "syrian arab republic" OR tajikistan OR tadjikistan OR tadzhikistan OR tadzhik OR tanzania OR Tanganyika OR thailand OR siam OR "timor leste" OR "east timor" OR togo OR "togolese republic" OR tonga OR trinidad OR tobago OR tunisia OR turkey OR turkmenistan OR turkmen OR uganda OR ukraine OR uruguay OR uzbekistan OR uzbek OR vanuatu OR "new hebrides" OR venezuela OR vietnam OR "viet name" OR "west bank" OR gaza OR palestine OR yemen OR yugoslavia OR zambia OR zimbabwe OR "northern rhodesia")) OR (AB(afghanistan OR albania OR algeria OR "american samoa" OR angola OR antigua OR barbuda OR argentina OR armenia OR armenian OR aruba OR azerbaijan OR bahrain OR bangladesh OR barbados OR belarus OR byelarus OR belorussia OR byelorussian OR belize OR "british honduras" OR benin OR dahomey OR bhutan OR bolivia OR bosnia OR herzegovina OR Botswana OR bechuanaland OR brazil OR brasil OR bulgaria OR "burkina faso" OR "burkina fasso" OR "upper volta" OR burundi OR urundi OR "cabo verde" OR "cape verde" OR cambodia OR kampuchea OR "khmer republic" OR cameroon OR cameron OR cameroun OR "central african republic" OR "ubangi shari" OR chad OR chile OR china OR colombia OR comoros OR "comoro islands" OR mayotte OR congo OR zaire OR "costa rica" OR "cote d'ivoire" OR "cote d'ivoire" OR "cote d'ivoire" OR "ivory coast" OR croatia OR cuba OR cyprus OR "czech republic" OR czechoslovakia OR djibouti OR "french somaliland" OR dominica OR "dominican republic" OR ecuador OR egypt OR "united arab republic" OR "el salvador" OR "equatorial guinea" OR "spanish guinea" OR eritrea OR estonia OR eswatini OR swaziland OR ethiopia OR fiji OR gabon OR "gabonese republic" OR gambia OR georgia OR Georgian OR ghana OR "gold coast" OR gibraltar OR greece OR grenada OR guam OR guatemala OR guinea OR guyana OR guiana OR haiti OR hispaniola OR honduras OR hungary OR india OR indonesia OR timor OR iran OR iraq OR "isle of man" OR jamaica OR jordan OR kazakhstan OR kazakh OR kenya OR korea OR kosovo OR kyrgyzstan OR kirghizia OR kirgizstan OR "kyrgyz republic" OR kirghiz OR laos OR "lao pdr" OR "lao people's democratic republic" OR latvia OR lebanon OR lesotho OR basutoland OR liberia OR libya OR "libyan arab jamahiriya" OR lithuania OR macau OR macao OR macedonia OR madagascar OR "malagasy republic" OR malawi OR nyasaland OR malaysia OR maldives OR "indian ocean" OR mali OR malta OR micronesia OR kiribati OR "marshall islands" OR nauru OR "northern mariana islands" OR palau OR tuvalu OR mauritania OR mauritius OR mexico OR moldova OR moldovian OR mongolia OR montenegro OR morocco OR ifni OR mozambique OR "portuguese east africa" OR myanmar OR burma OR namibia OR nepal OR "netherlands antilles" OR nicaragua OR niger OR nigeria OR oman OR muscat OR pakistan OR panama OR "papua new guinea" OR paraguay OR peru OR philippines OR philipines OR phillipines OR phillippines OR poland OR "polish people's republic" OR portugal OR "portuguese republic" OR "puerto rico" OR romania OR russia OR "russian federation" OR ussr OR "soviet union" OR "union of soviet socialist republics" OR rwanda OR ruanda OR samoa OR "pacific islands" OR polynesia OR "samoan islands" OR "sao tome" and principe OR "saudi arabia" OR senegal OR serbia OR seychelles OR "sierra leone" OR slovakia OR "slovak republic" OR slovenia OR melanesia OR "solomon island" OR "solomon islands" OR "norfolk island" OR somalia OR "south africa" OR "south sudan" OR "sri lanka" OR ceylon OR "saint kitts" OR nevis OR "st kitts" OR nevis OR "saint lucia" OR "st lucia" OR "saint vincent" OR "st vincent" OR grenadines OR sudan OR suriname OR surinam OR syria OR "syrian OR muscat OR pakistan OR panama OR "papua new guinea" OR paraguay OR peru OR philippines OR philipines OR phillipines OR phillippines OR poland OR "polish people's republic" OR portugal OR "portuguese republic" OR "puerto rico" OR romania OR russia OR "russian federation" OR ussr OR "soviet union" OR "union of soviet socialist republics" OR rwanda OR ruanda OR samoa OR "pacific islands" OR polynesia OR "samoan islands" OR "sao tome" and principe OR "saudi arabia" OR senegal OR serbia OR seychelles OR "sierra leone" OR slovakia OR "slovak republic" OR slovenia OR melanesia OR "solomon island" OR "solomon islands" OR "norfolk island" OR somalia OR "south africa" OR "south sudan" OR "sri lanka" OR ceylon OR "saint kitts" OR nevis OR "st kitts" OR nevis OR "saint lucia" OR "st lucia" OR "saint vincent" OR "st vincent" OR grenadines OR sudan OR suriname OR surinam OR syria OR "syrian arab republic" OR tajikistan OR tadjikistan OR tadzhikistan OR tadzhik OR tanzania OR Tanganyika OR thailand OR siam OR "timor leste" OR "east timor" OR togo OR "togolese republic" OR tonga OR trinidad OR tobago OR tunisia OR turkey OR turkmenistan OR turkmen OR uganda OR ukraine OR uruguay OR uzbekistan OR uzbek OR vanuatu OR "new hebrides" OR venezuela OR vietnam OR "viet name" OR "west bank" OR gaza OR palestine OR yemen OR yugoslavia OR zambia OR zimbabwe OR "northern rhodesia")	1,932,289
S23	TI (("transitional country" OR "transitional countries")) OR AB (("transitional country" OR "transitional countries")) OR SU (("transitional country" OR "transitional countries"))	74
S22	TI ((emerging N0 (economy or economies or nation*))) OR AB ((emerging N0 (economy or economies or nation*))) OR SU ((emerging N0 (economy or economies or nation*)))	747
S21	TI (((low* N1 (gdp OR gnp OR "gross domestic" OR "gross national"))) OR AB (((low* N1 (gdp OR gnp OR "gross domestic" OR "gross national"))) OR SU (((low* N1 (gdp OR gnp OR "gross domestic" OR "gross national")))	66
S20	TI (low N3 middle N3 countr*) OR AB (low N3 middle N3 countr*) OR SU (low N3 middle N3 countr*)	6435
S19	(TI (((developing OR (less* N1 developed) OR "under developed" OR "middle income" or (low* N1 income)) N1 (economy or economies)))) OR (AB(((developing OR (less* N1 developed) OR "under developed" OR "middle income" or (low* N1 income)) N1 (economy or economies)))) OR (SU(((developing OR (less* N1 developed) OR "under developed" OR "middle income" or (low* N1 income)) N1 (economy or economies))))	1028
S18	(TI (((developing OR (less* N1 developed) OR "under developed" OR underdeveloped OR "under served" OR underserved OR deprived OR poor* OR "middle income" or (low* N1 income)) N1 (countr* or nation* or population* or world)))) OR (AB(((developing OR (less* N1 developed) OR "under developed" OR underdeveloped OR "under served" OR underserved OR deprived OR poor* OR "middle income" or (low* N1 income)) N1 (countr* or nation* or population* or world)))) OR (SU (((developing OR (less* N1 developed) OR "under developed" OR underdeveloped OR "under served" OR underserved OR deprived OR poor* OR "middle income" or (low* N1 income)) N1 (countr* or nation* or population* or world))))	105,816
S17	(TI (Africa OR Asia OR Caribbean OR "West Indies" OR "South America" OR "Latin America" OR "Central America" OR "Middle East" OR "global south")) OR (AB(Africa OR Asia OR Caribbean OR "West Indies" OR "South America" OR "Latin America" OR "Central America" OR "Middle East" OR "global south")) OR (SU (Africa OR Asia OR Caribbean OR "West Indies" OR "South America" OR "Latin America" OR "Central America" OR "Middle East" OR "global south"))	1,581,394
S16	AB ((women OR "women's right" OR "young women" OR woman* OR adolescent* OR girl* OR schoolgirl* OR "school girl*" OR female* OR man OR men OR "young men" OR boy* OR schoolboy* OR "school boy*" OR teen* OR youth* OR young* OR mother* OR father* OR parent* OR caregiver) OR (femini* OR masculi* OR female* OR male* OR gender* OR "social equality" OR "social equity" OR empower* OR "equal opportunit*" OR egalitarian*)) OR SU ((women OR "women's right" OR "young women" OR woman* OR adolescent* OR girl* OR schoolgirl* OR "school girl*" OR female* OR man OR men OR "young men" OR boy* OR schoolboy* OR "school boy*" OR teen* OR youth* OR young* OR mother* OR father* OR parent* OR caregiver) OR (femini* OR masculi* OR female* OR male* OR gender* OR "social equality" OR "social equity" OR empower* OR "equal opportunit*" OR egalitarian*)) OR TI ((women OR "women's right" OR "young women" OR woman* OR adolescent* OR girl* OR schoolgirl* OR "school girl*" OR female* OR man OR men OR "young men" OR boy* OR schoolboy* OR "school boy*" OR teen* OR youth* OR young* OR mother* OR father* OR parent* OR caregiver) OR (femini* OR masculi* OR female* OR male* OR gender* OR "social equality" OR "social equity" OR empower* OR "equal opportunit*" OR egalitarian*))	635,555
S15	S11 OR S12 OR S13 OR S14	43,252
S14	(TI (((systematic* OR synthes*) N3 (research OR evaluation* OR finding* OR thematic* OR report OR descriptive OR explanatory OR narrative OR meta* OR review* OR data OR literature OR studies OR evidence OR map OR quantitative OR study OR studies OR paper OR impact OR impacts OR effect* OR compar*))) OR (AB (((systematic* OR synthes*) N3 (research OR evaluation* OR finding* OR thematic* OR report OR descriptive OR explanatory OR narrative OR meta* OR review* OR data OR literature OR studies OR evidence OR map OR quantitative OR study OR studies OR paper OR impact OR impacts OR effect* OR compar*))) OR (SU(((systematic* OR synthes*) N3 (research OR evaluation* OR finding* OR thematic* OR report OR descriptive OR explanatory OR narrative OR meta* OR review* OR data OR literature OR studies OR evidence OR map OR quantitative OR study OR studies OR paper OR impact OR impacts OR effect* OR compar*)))	20,533
S13	(TI ((("meta regression" OR "meta synth*" OR "meta‐synth*" OR "meta analy*" OR "metaanaly*" OR "meta‐analy*" OR "metanaly*" OR "metaregression" OR "meta‐regression" OR "methodologic* overview" OR "pool* analys*" OR "pool* data" OR "quantitative* overview" OR "research integration"))) OR (AB ((("meta regression" OR "meta synth*" OR "meta‐synth*" OR "meta analy*" OR "metaanaly*" OR "meta‐analy*" OR "metanaly*" OR "metaregression" OR "meta‐regression" OR "methodologic* overview" OR "pool* analys*" OR "pool* data" OR "quantitative* overview" OR "research integration"))) OR (SU((("meta regression" OR "meta synth*" OR "meta‐synth*" OR "meta analy*" OR "metaanaly*" OR "meta‐analy*" OR "metanaly*" OR "metaregression" OR "meta‐regression" OR "methodologic* overview" OR "pool* analys*" OR "pool* data" OR "quantitative* overview" OR "research integration")))	8015
S12	(TI ((review N3 (effectiveness OR effects OR systemat* OR synth* OR integrat* OR map* OR methodologic* OR quantitative OR evidence OR literature))) OR (AB ((review N3 (effectiveness OR effects OR systemat* OR synth* OR integrat* OR map* OR methodologic* OR quantitative OR evidence OR literature))) OR (SU ((review N3 (effectiveness OR effects OR systemat* OR synth* OR integrat* OR map* OR methodologic* OR quantitative OR evidence OR literature)))	27,691
S11	(TI (("literature search" OR "database search" OR "bibliographic* search" OR comprehensive search" OR “extensive search" OR "exhaustive search" OR "purposive search" OR "representative search" OR "systemat* search")) OR (AB (("literature search" OR "database search" OR "bibliographic* search" OR comprehensive search" OR “extensive search" OR "exhaustive search" OR "purposive search" OR "representative search" OR "systemat* search")) OR (SU (("literature search" OR "database search" OR "bibliographic* search" OR comprehensive search" OR “extensive search" OR "exhaustive search" OR "purposive search" OR "representative search" OR "systemat* search"))	2230
S10	S2 OR S3 OR S4 OR S5 OR S6 OR S7 OR S8 OR S9	33,834
S9	TI ((((family OR birth OR income) N1(allowance OR maintenance OR grant OR payment)) OR (leave N1(parental OR paternity OR maternity OR sick)) OR (welfare N1(infant OR maternal)) OR (support N1(disability OR nutritional OR compensatory)) OR (voucher N1(food OR school OR education)) OR (assistance N1(humanitarian)) OR (service* N2("ancillary school" OR homemaker OR "school health" OR "child care*" OR community OR "home care" OR "adult protective")) OR(aid N4 ("to families with dependent children" OR "to the totally disabled" OR "to visually impaired")) OR (community* N1(care OR intervention OR integration OR project)) OR (care N2(day OR "for children" OR "for elderly" OR "foster home" OR "centre‐based child" OR "center‐based child")) OR (education N1(parenting OR vocational)) OR (tax N1(exemption OR break OR credit*)) OR ("after school club" OR "after school program*" OR family support)))	2030
S8	AB ((((family OR birth OR income) N1(allowance OR maintenance OR grant OR payment)) OR (leave N1(parental OR paternity OR maternity OR sick)) OR (welfare N1(infant OR maternal)) OR (support N1(disability OR nutritional OR compensatory)) OR (voucher N1(food OR school OR education)) OR (assistance N1(humanitarian)) OR (service* N2("ancillary school" OR homemaker OR "school health" OR "child care*" OR community OR "home care" OR "adult protective")) OR(aid N4 ("to families with dependent children" OR "to the totally disabled" OR "to visually impaired")) OR (community* N1(care OR intervention OR integration OR project)) OR (care N2(day OR "for children" OR "for elderly" OR "foster home" OR "centre‐based child" OR "center‐based child")) OR (education N1(parenting OR vocational)) OR (tax N1(exemption OR break OR credit*)) OR ("after school club" OR "after school program*" OR family support)))	4436
S7	SU ((((family OR birth OR income) N1(allowance OR maintenance OR grant OR payment)) OR (leave N1(parental OR paternity OR maternity OR sick)) OR (welfare N1(infant OR maternal)) OR (support N1(disability OR nutritional OR compensatory)) OR (voucher N1(food OR school OR education)) OR (assistance N1(humanitarian)) OR (service* N2("ancillary school" OR homemaker OR "school health" OR "child care*" OR community OR "home care" OR "adult protective")) OR(aid N4 ("to families with dependent children" OR "to the totally disabled" OR "to visually impaired")) OR (community* N1(care OR intervention OR integration OR project)) OR (care N2(day OR "for children" OR "for elderly" OR "foster home" OR "centre‐based child" OR "center‐based child")) OR (education N1(parenting OR vocational)) OR (tax N1(exemption OR break OR credit*)) OR ("after school club" OR "after school program*" OR family support)))	2385
S6	SU ((("economic asset" OR "fee waiver*" OR "school feeding" OR "disability grant*" OR "food stamp" OR "sick pay" OR medicaid OR medicare OR "home health nursing" OR "nurse family partnership") OR (intervention* N3("intimate partner violence" OR "domestic violence" OR "domestic abuse" OR ipv OR "family violence" OR "partner violence" OR "spous* abuse" OR "battered women" OR abusive)))) OR AB ((("economic asset" OR "fee waiver*" OR "school feeding" OR "disability grant*" OR "food stamp" OR "sick pay" OR medicaid OR medicare OR "home health nursing" OR "nurse family partnership") OR (intervention* N3("intimate partner violence" OR "domestic violence" OR "domestic abuse" OR ipv OR "family violence" OR "partner violence" OR "spous* abuse" OR "battered women" OR abusive)))) OR TI ((("economic asset" OR "fee waiver*" OR "school feeding" OR "disability grant*" OR "food stamp" OR "sick pay" OR medicaid OR medicare OR "home health nursing" OR "nurse family partnership") OR (intervention* N3("intimate partner violence" OR "domestic violence" OR "domestic abuse" OR ipv OR "family violence" OR "partner violence" OR "spous* abuse" OR "battered women" OR abusive))))	613
S5	SU (((labo#r market N1(policy OR policies OR program* OR reform * OR standard OR supply)) OR (employment N1(scheme OR support OR service)) OR (training N1 (vocational OR job OR support OR employee)) OR (subsid* N1(wage OR hiring OR health)) OR ("activation policies" OR "skills development" OR "skill development" or "entrepreneurship promotion" or "public works") OR (insurance N2 (health OR household OR community‐based OR national OR unemployment OR micro‐health OR "micro health")) OR (child NEAR/2(support OR welfare OR grant OR care OR "care cash")) OR (assistance N2 (medical OR employee OR nutrition OR food OR public OR social OR "old age")) OR (benefit* N1(paternity OR maternity OR system OR retirement OR unemployment OR fringe OR employee OR health OR child OR death OR system OR insurance)) OR ((wage OR housing) N1(subsidy OR subsidies OR public OR elderly))))	3310
S4	AB (((labo#r market N1(policy OR policies OR program* OR reform * OR standard OR supply)) OR (employment N1(scheme OR support OR service)) OR (training N1 (vocational OR job OR support OR employee)) OR (subsid* N1(wage OR hiring OR health)) OR ("activation policies" OR "skills development" OR "skill development" or "entrepreneurship promotion" or "public works") OR (insurance N2 (health OR household OR community‐based OR national OR unemployment OR micro‐health OR "micro health")) OR (child NEAR/2(support OR welfare OR grant OR care OR "care cash")) OR (assistance N2 (medical OR employee OR nutrition OR food OR public OR social OR "old age")) OR (benefit* N1(paternity OR maternity OR system OR retirement OR unemployment OR fringe OR employee OR health OR child OR death OR system OR insurance)) OR ((wage OR housing) N1(subsidy OR subsidies OR public OR elderly))))	4692
S3	TI(((labo#r market N1(policy OR policies OR program* OR reform * OR standard OR supply)) OR (employment N1(scheme OR support OR service)) OR (training N1 (vocational OR job OR support OR employee)) OR (subsid* N1(wage OR hiring OR health)) OR ("activation policies" OR "skills development" OR "skill development" or "entrepreneurship promotion" or "public works") OR (insurance N2 (health OR household OR community‐based OR national OR unemployment OR micro‐health OR "micro health")) OR (child NEAR/2(support OR welfare OR grant OR care OR "care cash")) OR (assistance N2 (medical OR employee OR nutrition OR food OR public OR social OR "old age")) OR (benefit* N1(paternity OR maternity OR system OR retirement OR unemployment OR fringe OR employee OR health OR child OR death OR system OR insurance)) OR ((wage OR housing) N1(subsidy OR subsidies OR public OR elderly))))	1364
S2	SU ((((social N1(care* OR protection OR insurance OR policy OR policies OR inclusion OR security or assistance or service* OR welfare OR grant OR pension* OR "safety net"))) OR (program* N2 ("cash plus" OR lunch OR breakfast OR "school health" OR "food security" OR "poverty alleviation" OR "poverty reduction" OR livelihood* OR "employee assistance" OR employment OR empowerment OR entrepreneurship OR enterpreneurship OR employment OR "employee assistance" OR "youth employment" OR integrated OR community‐based)) OR (transfer N1(resource OR in‐kind OR conditional OR unconditional OR financial OR monetary)) OR (pension N1(retirement OR public OR "old age")) OR (financial N1(incentive OR protection)))) OR AB ((((social N1(care* OR protection OR insurance OR policy OR policies OR inclusion OR security or assistance or service* OR welfare OR grant OR pension* OR "safety net"))) OR (program* N2 ("cash plus" OR lunch OR breakfast OR "school health" OR "food security" OR "poverty alleviation" OR "poverty reduction" OR livelihood* OR "employee assistance" OR employment OR empowerment OR entrepreneurship OR enterpreneurship OR employment OR "employee assistance" OR "youth employment" OR integrated OR community‐based)) OR (transfer N1(resource OR in‐kind OR conditional OR unconditional OR financial OR monetary)) OR (pension N1(retirement OR public OR "old age")) OR (financial N1(incentive OR protection)))) OR TI ((((social N1(care* OR protection OR insurance OR policy OR policies OR inclusion OR security or assistance or service* OR welfare OR grant OR pension* OR "safety net"))) OR (program* N2 ("cash plus" OR lunch OR breakfast OR "school health" OR "food security" OR "poverty alleviation" OR "poverty reduction" OR livelihood* OR "employee assistance" OR employment OR empowerment OR entrepreneurship OR enterpreneurship OR employment OR "employee assistance" OR "youth employment" OR integrated OR community‐based)) OR (transfer N1(resource OR in‐kind OR conditional OR unconditional OR financial OR monetary)) OR (pension N1(retirement OR public OR "old age")) OR (financial N1(incentive OR protection))))	4895

## APPENDIX C ECONLIT

Name of database: EconLit

Date of search: 25/02/2021

Number of results: 188

**Table:**

#	Search String	Results
S26	S10 AND S15 AND S16 AND S25	**188**
S25	S17 OR S18 OR S19 OR S20 OR S21 OR S22 OR S23 OR S24	458,271
S24	(TI(afghanistan OR albania OR algeria OR "american samoa" OR angola OR antigua OR barbuda OR argentina OR armenia OR armenian OR aruba OR azerbaijan OR bahrain OR bangladesh OR barbados OR belarus OR byelarus OR belorussia OR byelorussian OR belize OR "british honduras" OR benin OR dahomey OR bhutan OR bolivia OR bosnia OR herzegovina OR Botswana OR bechuanaland OR brazil OR brasil OR bulgaria OR "burkina faso" OR "burkina fasso" OR "upper volta" OR burundi OR urundi OR "cabo verde" OR "cape verde" OR cambodia OR kampuchea OR "khmer republic" OR cameroon OR cameron OR cameroun OR "central african republic" OR "ubangi shari" OR chad OR chile OR china OR colombia OR comoros OR "comoro islands" OR mayotte OR congo OR zaire OR "costa rica" OR "cote d'ivoire" OR "cote d'ivoire" OR "cote d'ivoire" OR "ivory coast" OR croatia OR cuba OR cyprus OR "czech republic" OR czechoslovakia OR djibouti OR "french somaliland" OR dominica OR "dominican republic" OR ecuador OR egypt OR "united arab republic" OR "el salvador" OR "equatorial guinea" OR "spanish guinea" OR eritrea OR estonia OR eswatini OR swaziland OR ethiopia OR fiji OR gabon OR "gabonese republic" OR gambia OR georgia OR Georgian OR ghana OR "gold coast" OR gibraltar OR greece OR grenada OR guam OR guatemala OR guinea OR guyana OR guiana OR haiti OR hispaniola OR honduras OR hungary OR india OR indonesia OR timor OR iran OR iraq OR "isle of man" OR jamaica OR jordan OR kazakhstan OR kazakh OR kenya OR korea OR kosovo OR kyrgyzstan OR kirghizia OR kirgizstan OR "kyrgyz republic" OR kirghiz OR laos OR "lao pdr" OR "lao people's democratic republic" OR latvia OR lebanon OR lesotho OR basutoland OR liberia OR libya OR "libyan arab jamahiriya" OR lithuania OR macau OR macao OR macedonia OR madagascar OR "malagasy republic" OR malawi OR nyasaland OR malaysia OR maldives OR "indian ocean" OR mali OR malta OR micronesia OR kiribati OR "marshall islands" OR nauru OR "northern mariana islands" OR palau OR tuvalu OR mauritania OR mauritius OR mexico OR moldova OR moldovian OR mongolia OR montenegro OR morocco OR ifni OR mozambique OR "portuguese east africa" OR myanmar OR burma OR namibia OR nepal OR "netherlands antilles" OR nicaragua OR niger OR nigeria OR oman OR muscat OR pakistan OR panama OR "papua new guinea" OR paraguay OR peru OR philippines OR philipines OR phillipines OR phillippines OR poland OR "polish people's republic" OR portugal OR "portuguese republic" OR "puerto rico" OR romania OR russia OR "russian federation" OR ussr OR "soviet union" OR "union of soviet socialist republics" OR rwanda OR ruanda OR samoa OR "pacific islands" OR polynesia OR "samoan islands" OR "sao tome" and principe OR "saudi arabia" OR senegal OR serbia OR seychelles OR "sierra leone" OR slovakia OR "slovak republic" OR slovenia OR melanesia OR "solomon island" OR "solomon islands" OR "norfolk island" OR somalia OR "south africa" OR "south sudan" OR "sri lanka" OR ceylon OR "saint kitts" OR nevis OR "st kitts" OR nevis OR "saint lucia" OR "st lucia" OR "saint vincent" OR "st vincent" OR grenadines OR sudan OR suriname OR surinam OR syria OR "syrian OR muscat OR pakistan OR panama OR "papua new guinea" OR paraguay OR peru OR philippines OR philipines OR phillipines OR phillippines OR poland OR "polish people's republic" OR portugal OR "portuguese republic" OR "puerto rico" OR romania OR russia OR "russian federation" OR ussr OR "soviet union" OR "union of soviet socialist republics" OR rwanda OR ruanda OR samoa OR "pacific islands" OR polynesia OR "samoan islands" OR "sao tome" and principe OR "saudi arabia" OR senegal OR serbia OR seychelles OR "sierra leone" OR slovakia OR "slovak republic" OR slovenia OR melanesia OR "solomon island" OR "solomon islands" OR "norfolk island" OR somalia OR "south africa" OR "south sudan" OR "sri lanka" OR ceylon OR "saint kitts" OR nevis OR "st kitts" OR nevis OR "saint lucia" OR "st lucia" OR "saint vincent" OR "st vincent" OR grenadines OR sudan OR suriname OR surinam OR syria OR "syrian arab republic" OR tajikistan OR tadjikistan OR tadzhikistan OR tadzhik OR tanzania OR Tanganyika OR thailand OR siam OR "timor leste" OR "east timor" OR togo OR "togolese republic" OR tonga OR trinidad OR tobago OR tunisia OR turkey OR turkmenistan OR turkmen OR uganda OR ukraine OR uruguay OR uzbekistan OR uzbek OR vanuatu OR "new hebrides" OR venezuela OR vietnam OR "viet name" OR "west bank" OR gaza OR palestine OR yemen OR yugoslavia OR zambia OR zimbabwe OR "northern rhodesia") OR **AB** (afghanistan OR albania OR algeria OR "american samoa" OR angola OR antigua OR barbuda OR argentina OR armenia OR armenian OR aruba OR azerbaijan OR bahrain OR bangladesh OR barbados OR belarus OR byelarus OR belorussia OR byelorussian OR belize OR "british honduras" OR benin OR dahomey OR bhutan OR bolivia OR bosnia OR herzegovina OR Botswana OR bechuanaland OR brazil OR brasil OR bulgaria OR "burkina faso" OR "burkina fasso" OR "upper volta" OR burundi OR urundi OR "cabo verde" OR "cape verde" OR cambodia OR kampuchea OR "khmer republic" OR cameroon OR cameron OR cameroun OR "central african republic" OR "ubangi shari" OR chad OR chile OR china OR colombia OR comoros OR "comoro islands" OR mayotte OR congo OR zaire OR "costa rica" OR "cote d'ivoire" OR "cote d'ivoire" OR "cote d'ivoire" OR "ivory coast" OR croatia OR cuba OR cyprus OR "czech republic" OR czechoslovakia OR djibouti OR "french somaliland" OR dominica OR "dominican republic" OR ecuador OR egypt OR "united arab republic" OR "el salvador" OR "equatorial guinea" OR "spanish guinea" OR eritrea OR estonia OR eswatini OR swaziland OR ethiopia OR fiji OR gabon OR "gabonese republic" OR gambia OR georgia OR Georgian OR ghana OR "gold coast" OR gibraltar OR greece OR grenada OR guam OR guatemala OR guinea OR guyana OR guiana OR haiti OR hispaniola OR honduras OR hungary OR india OR indonesia OR timor OR iran OR iraq OR "isle of man" OR jamaica OR jordan OR kazakhstan OR kazakh OR kenya OR korea OR kosovo OR kyrgyzstan OR kirghizia OR kirgizstan OR "kyrgyz republic" OR kirghiz OR laos OR "lao pdr" OR "lao people's democratic republic" OR latvia OR lebanon OR lesotho OR basutoland OR liberia OR libya OR "libyan arab jamahiriya" OR lithuania OR macau OR macao OR macedonia OR madagascar OR "malagasy republic" OR malawi OR nyasaland OR malaysia OR maldives OR "indian ocean" OR mali OR malta OR micronesia OR kiribati OR "marshall islands" OR nauru OR "northern mariana islands" OR palau OR tuvalu OR mauritania OR mauritius OR mexico OR moldova OR moldovian OR mongolia OR montenegro OR morocco OR ifni OR mozambique OR "portuguese east africa" OR myanmar OR burma OR namibia OR nepal OR "netherlands antilles" OR nicaragua OR niger OR nigeria OR oman OR muscat OR pakistan OR panama OR "papua new guinea" OR paraguay OR peru OR philippines OR philipines OR phillipines OR phillippines OR poland OR "polish people's republic" OR portugal OR "portuguese republic" OR "puerto rico" OR romania OR russia OR "russian federation" OR ussr OR "soviet union" OR "union of soviet socialist republics" OR rwanda OR ruanda OR samoa OR "pacific islands" OR polynesia OR "samoan islands" OR "sao tome" and principe OR "saudi arabia" OR senegal OR serbia OR seychelles OR "sierra leone" OR slovakia OR "slovak republic" OR slovenia OR melanesia OR "solomon island" OR "solomon islands" OR "norfolk island" OR somalia OR "south africa" OR "south sudan" OR "sri lanka" OR ceylon OR "saint kitts" OR nevis OR "st kitts" OR nevis OR "saint lucia" OR "st lucia" OR "saint vincent" OR "st vincent" OR grenadines OR sudan OR suriname OR surinam OR syria OR "syrian OR muscat OR pakistan OR panama OR "papua new guinea" OR paraguay OR peru OR philippines OR philipines OR phillipines OR phillippines OR poland OR "polish people's republic" OR portugal OR "portuguese republic" OR "puerto rico" OR romania OR russia OR "russian federation" OR ussr OR "soviet union" OR "union of soviet socialist republics" OR rwanda OR ruanda OR samoa OR "pacific islands" OR polynesia OR "samoan islands" OR "sao tome" and principe OR "saudi arabia" OR senegal OR serbia OR seychelles OR "sierra leone" OR slovakia OR "slovak republic" OR slovenia OR melanesia OR "solomon island" OR "solomon islands" OR "norfolk island" OR somalia OR "south africa" OR "south sudan" OR "sri lanka" OR ceylon OR "saint kitts" OR nevis OR "st kitts" OR nevis OR "saint lucia" OR "st lucia" OR "saint vincent" OR "st vincent" OR grenadines OR sudan OR suriname OR surinam OR syria OR "syrian arab republic" OR tajikistan OR tadjikistan OR tadzhikistan OR tadzhik OR tanzania OR Tanganyika OR thailand OR siam OR "timor leste" OR "east timor" OR togo OR "togolese republic" OR tonga OR trinidad OR tobago OR tunisia OR turkey OR turkmenistan OR turkmen OR uganda OR ukraine OR uruguay OR uzbekistan OR uzbek OR vanuatu OR "new hebrides" OR venezuela OR vietnam OR "viet name" OR "west bank" OR gaza OR palestine OR yemen OR yugoslavia OR zambia OR zimbabwe OR "northern rhodesia")) OR (**SU**(afghanistan OR albania OR algeria OR "american samoa" OR angola OR antigua OR barbuda OR argentina OR armenia OR armenian OR aruba OR azerbaijan OR bahrain OR bangladesh OR barbados OR belarus OR byelarus OR belorussia OR byelorussian OR belize OR "british honduras" OR benin OR dahomey OR bhutan OR bolivia OR bosnia OR herzegovina OR Botswana OR bechuanaland OR brazil OR brasil OR bulgaria OR "burkina faso" OR "burkina fasso" OR "upper volta" OR burundi OR urundi OR "cabo verde" OR "cape verde" OR cambodia OR kampuchea OR "khmer republic" OR cameroon OR cameron OR cameroun OR "central african republic" OR "ubangi shari" OR chad OR chile OR china OR colombia OR comoros OR "comoro islands" OR mayotte OR congo OR zaire OR "costa rica" OR "cote d'ivoire" OR "cote d'ivoire" OR "cote d'ivoire" OR "ivory coast" OR croatia OR cuba OR cyprus OR "czech republic" OR czechoslovakia OR djibouti OR "french somaliland" OR dominica OR "dominican republic" OR ecuador OR egypt OR "united arab republic" OR "el salvador" OR "equatorial guinea" OR "spanish guinea" OR eritrea OR estonia OR eswatini OR swaziland OR ethiopia OR fiji OR gabon OR "gabonese republic" OR gambia OR georgia OR Georgian OR ghana OR "gold coast" OR gibraltar OR greece OR grenada OR guam OR guatemala OR guinea OR guyana OR guiana OR haiti OR hispaniola OR honduras OR hungary OR india OR indonesia OR timor OR iran OR iraq OR "isle of man" OR jamaica OR jordan OR kazakhstan OR kazakh OR kenya OR korea OR kosovo OR kyrgyzstan OR kirghizia OR kirgizstan OR "kyrgyz republic" OR kirghiz OR laos OR "lao pdr" OR "lao people's democratic republic" OR latvia OR lebanon OR lesotho OR basutoland OR liberia OR libya OR "libyan arab jamahiriya" OR lithuania OR macau OR macao OR macedonia OR madagascar OR "malagasy republic" OR malawi OR nyasaland OR malaysia OR maldives OR "indian ocean" OR mali OR malta OR micronesia OR kiribati OR "marshall islands" OR nauru OR "northern mariana islands" OR palau OR tuvalu OR mauritania OR mauritius OR mexico OR moldova OR moldovian OR mongolia OR montenegro OR morocco OR ifni OR mozambique OR "portuguese east africa" OR myanmar OR burma OR namibia OR nepal OR "netherlands antilles" OR nicaragua OR niger OR nigeria OR oman OR muscat OR pakistan OR panama OR "papua new guinea" OR paraguay OR peru OR philippines OR philipines OR phillipines OR phillippines OR poland OR "polish people's republic" OR portugal OR "portuguese republic" OR "puerto rico" OR romania OR russia OR "russian federation" OR ussr OR "soviet union" OR "union of soviet socialist republics" OR rwanda OR ruanda OR samoa OR "pacific islands" OR polynesia OR "samoan islands" OR "sao tome" and principe OR "saudi arabia" OR senegal OR serbia OR seychelles OR "sierra leone" OR slovakia OR "slovak republic" OR slovenia OR melanesia OR "solomon island" OR "solomon islands" OR "norfolk island" OR somalia OR "south africa" OR "south sudan" OR "sri lanka" OR ceylon OR "saint kitts" OR nevis OR "st kitts" OR nevis OR "saint lucia" OR "st lucia" OR "saint vincent" OR "st vincent" OR grenadines OR sudan OR suriname OR surinam OR syria OR "syrian OR muscat OR pakistan OR panama OR "papua new guinea" OR paraguay OR peru OR philippines OR philipines OR phillipines OR phillippines OR poland OR "polish people's republic" OR portugal OR "portuguese republic" OR "puerto rico" OR romania OR russia OR "russian federation" OR ussr OR "soviet union" OR "union of soviet socialist republics" OR rwanda OR ruanda OR samoa OR "pacific islands" OR polynesia OR "samoan islands" OR "sao tome" and principe OR "saudi arabia" OR senegal OR serbia OR seychelles OR "sierra leone" OR slovakia OR "slovak republic" OR slovenia OR melanesia OR "solomon island" OR "solomon islands" OR "norfolk island" OR somalia OR "south africa" OR "south sudan" OR "sri lanka" OR ceylon OR "saint kitts" OR nevis OR "st kitts" OR nevis OR "saint lucia" OR "st lucia" OR "saint vincent" OR "st vincent" OR grenadines OR sudan OR suriname OR surinam OR syria OR "syrian arab republic" OR tajikistan OR tadjikistan OR tadzhikistan OR tadzhik OR tanzania OR Tanganyika OR thailand OR siam OR "timor leste" OR "east timor" OR togo OR "togolese republic" OR tonga OR trinidad OR tobago OR tunisia OR turkey OR turkmenistan OR turkmen OR uganda OR ukraine OR uruguay OR uzbekistan OR uzbek OR vanuatu OR "new hebrides" OR venezuela OR vietnam OR "viet name" OR "west bank" OR gaza OR palestine OR yemen OR yugoslavia OR zambia OR zimbabwe OR "northern rhodesia")	340,237
S23	TI (("transitional country" OR "transitional countries")) OR AB (("transitional country" OR "transitional countries")) OR SU (("transitional country" OR "transitional countries"))	219
S22	TI ((emerging N0 (economy or economies or nation*))) OR AB ((emerging N0 (economy or economies or nation*))) OR SU ((emerging N0 (economy or economies or nation*)))	4843
S21	TI (((low* N1 (gdp OR gnp OR "gross domestic" OR "gross national"))) OR AB (((low* N1 (gdp OR gnp OR "gross domestic" OR "gross national"))) OR SU (((low* N1 (gdp OR gnp OR "gross domestic" OR "gross national")))	334
S20	TI (low N3 middle N3 countr*) OR AB (low N3 middle N3 countr*) OR SU (low N3 middle N3 countr*)	1194
S19	(TI (((developing OR (less* N1 developed) OR "under developed" OR "middle income" or (low* N1 income)) N1 (economy or economies)))) OR (AB(((developing OR (less* N1 developed) OR "under developed" OR "middle income" or (low* N1 income)) N1 (economy or economies)))) OR (SU(((developing OR (less* N1 developed) OR "under developed" OR "middle income" or (low* N1 income)) N1 (economy or economies))))	5385
S18	(TI (((developing OR (less* N1 developed) OR "under developed" OR underdeveloped OR "under served" OR underserved OR deprived OR poor* OR "middle income" or (low* N1 income)) N1 (countr* or nation* or population* or world)))) OR (AB(((developing OR (less* N1 developed) OR "under developed" OR underdeveloped OR "under served" OR underserved OR deprived OR poor* OR "middle income" or (low* N1 income)) N1 (countr* or nation* or population* or world)))) OR (SU (((developing OR (less* N1 developed) OR "under developed" OR underdeveloped OR "under served" OR underserved OR deprived OR poor* OR "middle income" or (low* N1 income)) N1 (countr* or nation* or population* or world))))	50,439
S17	(TI (Africa OR Asia OR Caribbean OR "West Indies" OR "South America" OR "Latin America" OR "Central America" OR "Middle East" OR "global south")) OR (AB(Africa OR Asia OR Caribbean OR "West Indies" OR "South America" OR "Latin America" OR "Central America" OR "Middle East" OR "global south")) OR (SU (Africa OR Asia OR Caribbean OR "West Indies" OR "South America" OR "Latin America" OR "Central America" OR "Middle East" OR "global south"))	318,273
S16	TI ((women OR "women's right" OR "young women" OR woman* OR adolescent* OR girl* OR schoolgirl* OR "school girl*" OR female* OR man OR men OR "young men" OR boy* OR schoolboy* OR "school boy*" OR teen* OR youth* OR young* OR mother* OR father* OR parent* OR caregiver) OR (femini* OR masculi* OR female* OR male* OR gender* OR "social equality" OR "social equity" OR empower* OR "equal opportunit*" OR egalitarian*)) OR AB ((women OR "women's right" OR "young women" OR woman* OR adolescent* OR girl* OR schoolgirl* OR "school girl*" OR female* OR man OR men OR "young men" OR boy* OR schoolboy* OR "school boy*" OR teen* OR youth* OR young* OR mother* OR father* OR parent* OR caregiver) OR (femini* OR masculi* OR female* OR male* OR gender* OR "social equality" OR "social equity" OR empower* OR "equal opportunit*" OR egalitarian*)) OR SU ((women OR "women's right" OR "young women" OR woman* OR adolescent* OR girl* OR schoolgirl* OR "school girl*" OR female* OR man OR men OR "young men" OR boy* OR schoolboy* OR "school boy*" OR teen* OR youth* OR young* OR mother* OR father* OR parent* OR caregiver) OR (femini* OR masculi* OR female* OR male* OR gender* OR "social equality" OR "social equity" OR empower* OR "equal opportunit*" OR egalitarian*))	112,317
S15	S11 OR S12 OR S13 OR S14	18,744
S14	(TI (((systematic* OR synthes*) N3 (research OR evaluation* OR finding* OR thematic* OR report OR descriptive OR explanatory OR narrative OR meta* OR review* OR data OR literature OR studies OR evidence OR map OR quantitative OR study OR studies OR paper OR impact OR impacts OR effect* OR compar*))) OR (AB (((systematic* OR synthes*) N3 (research OR evaluation* OR finding* OR thematic* OR report OR descriptive OR explanatory OR narrative OR meta* OR review* OR data OR literature OR studies OR evidence OR map OR quantitative OR study OR studies OR paper OR impact OR impacts OR effect* OR compar*))) OR (SU(((systematic* OR synthes*) N3 (research OR evaluation* OR finding* OR thematic* OR report OR descriptive OR explanatory OR narrative OR meta* OR review* OR data OR literature OR studies OR evidence OR map OR quantitative OR study OR studies OR paper OR impact OR impacts OR effect* OR compar*)))	7128
S13	(TI ((("meta regression" OR "meta synth*" OR "meta‐synth*" OR "meta analy*" OR "metaanaly*" OR "meta‐analy*" OR "metanaly*" OR "metaregression" OR "meta‐regression" OR "methodologic* overview" OR "pool* analys*" OR "pool* data" OR "quantitative* overview" OR "research integration"))) OR (AB ((("meta regression" OR "meta synth*" OR "meta‐synth*" OR "meta analy*" OR "metaanaly*" OR "meta‐analy*" OR "metanaly*" OR "metaregression" OR "meta‐regression" OR "methodologic* overview" OR "pool* analys*" OR "pool* data" OR "quantitative* overview" OR "research integration"))) OR (SU((("meta regression" OR "meta synth*" OR "meta‐synth*" OR "meta analy*" OR "metaanaly*" OR "meta‐analy*" OR "metanaly*" OR "metaregression" OR "meta‐regression" OR "methodologic* overview" OR "pool* analys*" OR "pool* data" OR "quantitative* overview" OR "research integration")))	2255
S12	(TI ((review N3 (effectiveness OR effects OR systemat* OR synth* OR integrat* OR map* OR methodologic* OR quantitative OR evidence OR literature))) OR (AB ((review N3 (effectiveness OR effects OR systemat* OR synth* OR integrat* OR map* OR methodologic* OR quantitative OR evidence OR literature))) OR (SU ((review N3 (effectiveness OR effects OR systemat* OR synth* OR integrat* OR map* OR methodologic* OR quantitative OR evidence OR literature)))	11,333
S11	(TI (("literature search" OR "database search" OR "bibliographic* search" OR comprehensive search" OR “extensive search" OR "exhaustive search" OR "purposive search" OR "representative search" OR "systemat* search")) OR (AB (("literature search" OR "database search" OR "bibliographic* search" OR comprehensive search" OR “extensive search" OR "exhaustive search" OR "purposive search" OR "representative search" OR "systemat* search")) OR (SU (("literature search" OR "database search" OR "bibliographic* search" OR comprehensive search" OR “extensive search" OR "exhaustive search" OR "purposive search" OR "representative search" OR "systemat* search"))	8
S10	S1 OR S2 OR S3 OR S4 OR S5 OR S7 OR S8 OR S9	50,642
S9	SU ((((family OR birth OR income) N1(allowance OR maintenance OR grant OR payment)) OR (leave N1(parental OR paternity OR maternity OR sick)) OR (welfare N1(infant OR maternal)) OR (support N1(disability OR nutritional OR compensatory)) OR (voucher N1(food OR school OR education)) OR (assistance N1(humanitarian)) OR (service* N2("ancillary school" OR homemaker OR "school health" OR "child care*" OR community OR "home care" OR "adult protective")) OR(aid N4 ("to families with dependent children" OR "to the totally disabled" OR "to visually impaired")) OR (community* N1(care OR intervention OR integration OR project)) OR (care N2(day OR "for children" OR "for elderly" OR "foster home" OR "centre‐based child" OR "center‐based child")) OR (education N1(parenting OR vocational)) OR (tax N1(exemption OR break OR credit*)) OR ("after school club" OR "after school program*" OR family support)))	14,684
S8	AB ((((family OR birth OR income) N1(allowance OR maintenance OR grant OR payment)) OR (leave N1(parental OR paternity OR maternity OR sick)) OR (welfare N1(infant OR maternal)) OR (support N1(disability OR nutritional OR compensatory)) OR (voucher N1(food OR school OR education)) OR (assistance N1(humanitarian)) OR (service* N2("ancillary school" OR homemaker OR "school health" OR "child care*" OR community OR "home care" OR "adult protective")) OR(aid N4 ("to families with dependent children" OR "to the totally disabled" OR "to visually impaired")) OR (community* N1(care OR intervention OR integration OR project)) OR (care N2(day OR "for children" OR "for elderly" OR "foster home" OR "centre‐based child" OR "center‐based child")) OR (education N1(parenting OR vocational)) OR (tax N1(exemption OR break OR credit*)) OR ("after school club" OR "after school program*" OR family support)))	4021
S7	TI ((((family OR birth OR income) N1(allowance OR maintenance OR grant OR payment)) OR (leave N1(parental OR paternity OR maternity OR sick)) OR (welfare N1(infant OR maternal)) OR (support N1(disability OR nutritional OR compensatory)) OR (voucher N1(food OR school OR education)) OR (assistance N1(humanitarian)) OR (service* N2("ancillary school" OR homemaker OR "school health" OR "child care*" OR community OR "home care" OR "adult protective")) OR(aid N4 ("to families with dependent children" OR "to the totally disabled" OR "to visually impaired")) OR (community* N1(care OR intervention OR integration OR project)) OR (care N2(day OR "for children" OR "for elderly" OR "foster home" OR "centre‐based child" OR "center‐based child")) OR (education N1(parenting OR vocational)) OR (tax N1(exemption OR break OR credit*)) OR ("after school club" OR "after school program*" OR family support)))	1100
S6	(TI ((((family OR birth OR income) N1(allowance OR maintenance OR grant OR payment)) OR (leave N1(parental OR paternity OR maternity OR sick)) OR (welfare N1(infant OR maternal)) OR (support N1(disability OR nutritional OR compensatory)) OR (voucher N1(food OR school OR education)) OR (assistance N1(humanitarian)) OR (service* N2("ancillary school" OR homemaker OR "school health" OR "child care*" OR community OR "home care" OR "adult protective")) OR(aid N4 ("to families with dependent children" OR "to the totally disabled" OR "to visually impaired")) OR (community* N1(care OR intervention OR integration OR project)) OR (care N2(day OR "for children" OR "for elderly" OR "foster home" OR "centre‐based child" OR "center‐based child")) OR (education N1(parenting OR vocational)) OR (tax N1(exemption OR break OR credit*)) OR ("after school club" OR "after school program*" OR family support)))) OR (AB ((((family OR birth OR income) N1(allowance OR maintenance OR grant OR payment)) OR (leave N1(parental OR paternity OR maternity OR sick)) OR (welfare N1(infant OR maternal)) OR (support N1(disability OR nutritional OR compensatory)) OR (voucher N1(food OR school OR education)) OR (assistance N1(humanitarian)) OR (service* N2("ancillary school" OR homemaker OR "school health" OR "child care*" OR community OR "home care" OR "adult protective")) OR(aid N4 ("to families with dependent children" OR "to the totally disabled" OR "to visually impaired")) OR (community* N1(care OR intervention OR integration OR project)) OR (care N2(day OR "for children" OR "for elderly" OR "foster home" OR "centre‐based child" OR "center‐based child")) OR (education N1(parenting OR vocational)) OR (tax N1(exemption OR break OR credit*)) OR ("after school club" OR "after school program*" OR family support)))) OR (SU ((((family OR birth OR income) N1(allowance OR maintenance OR grant OR payment)) OR (leave N1(parental OR paternity OR maternity OR sick)) OR (welfare N1(infant OR maternal)) OR (support N1(disability OR nutritional OR compensatory)) OR (voucher N1(food OR school OR education)) OR (assistance N1(humanitarian)) OR (service* N2("ancillary school" OR homemaker OR "school health" OR "child care*" OR community OR "home care" OR "adult protective")) OR(aid N4 ("to families with dependent children" OR "to the totally disabled" OR "to visually impaired")) OR (community* N1(care OR intervention OR integration OR project)) OR (care N2(day OR "for children" OR "for elderly" OR "foster home" OR "centre‐based child" OR "center‐based child")) OR (education N1(parenting OR vocational)) OR (tax N1(exemption OR break OR credit*)) OR ("after school club" OR "after school program*" OR family support))))	0
S5	(TI ((("economic asset" OR "fee waiver*" OR "school feeding" OR "disability grant*" OR "food stamp" OR "sick pay" OR medicaid OR medicare OR "home health nursing" OR "nurse family partnership") OR (intervention* N3("intimate partner violence" OR "domestic violence" OR "domestic abuse" OR ipv OR "family violence" OR "partner violence" OR "spous* abuse" OR "battered women" OR abusive))))) OR (AB ((("economic asset" OR "fee waiver*" OR "school feeding" OR "disability grant*" OR "food stamp" OR "sick pay" OR medicaid OR medicare OR "home health nursing" OR "nurse family partnership") OR (intervention* N3("intimate partner violence" OR "domestic violence" OR "domestic abuse" OR ipv OR "family violence" OR "partner violence" OR "spous* abuse" OR "battered women" OR abusive))))) OR (SU ((("economic asset" OR "fee waiver*" OR "school feeding" OR "disability grant*" OR "food stamp" OR "sick pay" OR medicaid OR medicare OR "home health nursing" OR "nurse family partnership") OR (intervention* N3("intimate partner violence" OR "domestic violence" OR "domestic abuse" OR ipv OR "family violence" OR "partner violence" OR "spous* abuse" OR "battered women" OR abusive)))))	2007
S4	SU (((labo#r market N1(policy OR policies OR program* OR reform * OR standard OR supply)) OR (employment N1(scheme OR support OR service)) OR (training N1 (vocational OR job OR support OR employee)) OR (subsid* N1(wage OR hiring OR health)) OR ("activation policies" OR "skills development" OR "skill development" or "entrepreneurship promotion" or "public works") OR (insurance N2 (health OR household OR community‐based OR national OR unemployment OR micro‐health OR "micro health")) OR (child NEAR/2(support OR welfare OR grant OR care OR "care cash")) OR (assistance N2 (medical OR employee OR nutrition OR food OR public OR social OR "old age")) OR (benefit* N1(paternity OR maternity OR system OR retirement OR unemployment OR fringe OR employee OR health OR child OR death OR system OR insurance)) OR ((wage OR housing) N1(subsidy OR subsidies OR public OR elderly))))	10,351
S3	AB (((labo#r market N1(policy OR policies OR program* OR reform * OR standard OR supply)) OR (employment N1(scheme OR support OR service)) OR (training N1 (vocational OR job OR support OR employee)) OR (subsid* N1(wage OR hiring OR health)) OR ("activation policies" OR "skills development" OR "skill development" or "entrepreneurship promotion" or "public works") OR (insurance N2 (health OR household OR community‐based OR national OR unemployment OR micro‐health OR "micro health")) OR (child NEAR/2(support OR welfare OR grant OR care OR "care cash")) OR (assistance N2 (medical OR employee OR nutrition OR food OR public OR social OR "old age")) OR (benefit* N1(paternity OR maternity OR system OR retirement OR unemployment OR fringe OR employee OR health OR child OR death OR system OR insurance)) OR ((wage OR housing) N1(subsidy OR subsidies OR public OR elderly))))	10,318
S2	TI (((labo#r market N1(policy OR policies OR program* OR reform * OR standard OR supply)) OR (employment N1(scheme OR support OR service)) OR (training N1 (vocational OR job OR support OR employee)) OR (subsid* N1(wage OR hiring OR health)) OR ("activation policies" OR "skills development" OR "skill development" or "entrepreneurship promotion" or "public works") OR (insurance N2 (health OR household OR community‐based OR national OR unemployment OR micro‐health OR "micro health")) OR (child NEAR/2(support OR welfare OR grant OR care OR "care cash")) OR (assistance N2 (medical OR employee OR nutrition OR food OR public OR social OR "old age")) OR (benefit* N1(paternity OR maternity OR system OR retirement OR unemployment OR fringe OR employee OR health OR child OR death OR system OR insurance)) OR ((wage OR housing) N1(subsidy OR subsidies OR public OR elderly))))	3853
S1	SU ((((social N1(care* OR protection OR insurance OR policy OR policies OR inclusion OR security or assistance or service* OR welfare OR grant OR pension* OR "safety net"))) OR (program* N2 ("cash plus" OR lunch OR breakfast OR "school health" OR "food security" OR "poverty alleviation" OR "poverty reduction" OR livelihood* OR "employee assistance" OR employment OR empowerment OR entrepreneurship OR enterpreneurship OR employment OR "employee assistance" OR "youth employment" OR integrated OR community‐based)) OR (transfer N1(resource OR in‐kind OR conditional OR unconditional OR financial OR monetary)) OR (pension N1(retirement OR public OR "old age")) OR (financial N1(incentive OR protection)))) OR AB ((((social N1(care* OR protection OR insurance OR policy OR policies OR inclusion OR security or assistance or service* OR welfare OR grant OR pension* OR "safety net"))) OR (program* N2 ("cash plus" OR lunch OR breakfast OR "school health" OR "food security" OR "poverty alleviation" OR "poverty reduction" OR livelihood* OR "employee assistance" OR employment OR empowerment OR entrepreneurship OR enterpreneurship OR employment OR "employee assistance" OR "youth employment" OR integrated OR community‐based)) OR (transfer N1(resource OR in‐kind OR conditional OR unconditional OR financial OR monetary)) OR (pension N1(retirement OR public OR "old age")) OR (financial N1(incentive OR protection)))) OR TI ((((social N1(care* OR protection OR insurance OR policy OR policies OR inclusion OR security or assistance or service* OR welfare OR grant OR pension* OR "safety net"))) OR (program* N2 ("cash plus" OR lunch OR breakfast OR "school health" OR "food security" OR "poverty alleviation" OR "poverty reduction" OR livelihood* OR "employee assistance" OR employment OR empowerment OR entrepreneurship OR enterpreneurship OR employment OR "employee assistance" OR "youth employment" OR integrated OR community‐based)) OR (transfer N1(resource OR in‐kind OR conditional OR unconditional OR financial OR monetary)) OR (pension N1(retirement OR public OR "old age")) OR (financial N1(incentive OR protection))))	22,335

## APPENDIX D ERIC

Name of database: Education Resources Information Centre (ERIC)

Date of search: 24/02/2021

Number of results: 50

**Table:**

#	Query	Results
S12	S4 AND S7 AND S10 AND S11	**50**
S11	(**TI** (("literature search" OR "database search" OR "bibliographic* search" OR comprehensive search" OR “extensive search" OR "exhaustive search" OR "purposive search" OR "representative search" OR "systemat* search") OR **AB** (("literature search" OR "database search" OR "bibliographic* search" OR comprehensive search" OR “extensive search" OR "exhaustive search" OR "purposive search" OR "representative search" OR "systemat* search") OR **SU** (("literature search" OR "database search" OR "bibliographic* search" OR comprehensive search" OR “extensive search" OR "exhaustive search" OR "purposive search" OR "representative search" OR "systemat* search")) OR (**TI** ((review N3 (effectiveness OR effects OR systemat* OR synth* OR integrat* OR map* OR methodologic* OR quantitative OR evidence OR literature)) OR **AB** ((review N3 (effectiveness OR effects OR systemat* OR synth* OR integrat* OR map* OR methodologic* OR quantitative OR evidence OR literature)) OR **SU** ((review N3 (effectiveness OR effects OR systemat* OR synth* OR integrat* OR map* OR methodologic* OR quantitative OR evidence OR literature))) OR (**TI** ((("meta regression" OR "meta synth*" OR "meta‐synth*" OR "meta analy*" OR "metaanaly*" OR "meta‐analy*" OR "metanaly*" OR "metaregression" OR "meta‐regression" OR "methodologic* overview" OR "pool* analys*" OR "pool* data" OR "quantitative* overview" OR "research integration"))) OR **AB** ((("meta regression" OR "meta synth*" OR "meta‐synth*" OR "meta analy*" OR "metaanaly*" OR "meta‐analy*" OR "metanaly*" OR "metaregression" OR "meta‐regression" OR "methodologic* overview" OR "pool* analys*" OR "pool* data" OR "quantitative* overview" OR "research integration"))) OR **SU** ((("meta regression" OR "meta synth*" OR "meta‐synth*" OR "meta analy*" OR "metaanaly*" OR "meta‐analy*" OR "metanaly*" OR "metaregression" OR "meta‐regression" OR "methodologic* overview" OR "pool* analys*" OR "pool* data" OR "quantitative* overview" OR "research integration")))) OR (**TI** (((systematic* OR synthes*) N3 (research OR evaluation* OR finding* OR thematic* OR report OR descriptive OR explanatory OR narrative OR meta* OR review* OR data OR literature OR studies OR evidence OR map OR quantitative OR study OR studies OR paper OR impact OR impacts OR effect* OR compar*))) OR **AB** (((systematic* OR synthes*) N3 (research OR evaluation* OR finding* OR thematic* OR report OR descriptive OR explanatory OR narrative OR meta* OR review* OR data OR literature OR studies OR evidence OR map OR quantitative OR study OR studies OR paper OR impact OR impacts OR effect* OR compar*))) OR **SU** (((systematic* OR synthes*) N3 (research OR evaluation* OR finding* OR thematic* OR report OR descriptive OR explanatory OR narrative OR meta* OR review* OR data OR literature OR studies OR evidence OR map OR quantitative OR study OR studies OR paper OR impact OR impacts OR effect* OR compar*))))	62,476
S10	S8 OR S9	460,641
S9	**TI** ((femini* OR masculi* OR female*OR male* OR gender* OR "social equality" OR "social equity" OR empower* OR "equal opportunit*" OR egalitarian*) OR **AB** ((femini* OR masculi* OR female*OR male* OR gender* OR "social equality" OR "social equity" OR empower* OR "equal opportunit*" OR egalitarian*) OR **SU** ((femini* OR masculi* OR female*OR male* OR gender* OR "social equality" OR "social equity" OR empower* OR "equal opportunit*" OR egalitarian*)	104,670
S8	**TI** (women OR "women's right" OR "young women" OR woman* OR adolescent* OR girl* OR schoolgirl* OR "school girl*" OR female* OR man OR men OR "young men" OR boy* OR schoolboy* OR "School boy*" OR teen* OR youth* OR young* OR mother* OR father* OR parent* OR caregiver) OR **AB** (women OR "women's right" OR "young women" OR woman* OR adolescent* OR girl* OR schoolgirl* OR "school girl*" OR female* OR man OR men OR "young men" OR boy* OR schoolboy* OR "School boy*" OR teen* OR youth* OR young* OR mother* OR father* OR parent* OR caregiver) OR **SU** (women OR "women's right" OR "young women" OR woman* OR adolescent* OR girl* OR schoolgirl* OR "school girl*" OR female* OR man OR men OR "young men" OR boy* OR schoolboy* OR "School boy*" OR teen* OR youth* OR young* OR mother* OR father* OR parent* OR caregiver)	417,196
S7	S5 OR S6	85,119
S6	**TI** ("Labour Market Policy" OR "Labour Market Policies" OR "Activation Policies" OR "Active Labour Market Policies" OR "Labour Market Program*" OR "Labour Market Reform*" OR "Labour Standard*" OR "Labour Supply Program*" OR "Labor Market Policy" OR "Labor market Policies" OR "Labor Market Program*" OR "Labor Market Reform*" OR "Labor Supply Program*" OR "Labor Standard*" OR "Livelihood Support" OR "Livelihood Program*" OR "Livelihood Project" OR "Livelihood Empowerment" OR "Poverty Reduction Program*" OR "Poverty Alleviation Program*" OR "Skills Development" OR "Skill Development" OR "Vocational Training" OR "Job Training" OR "Training Support" OR "Entrepreneurship Program*" OR "Entrepreneurship Promotion" OR "Employment Program*" OR "Employment Scheme*" OR "Employment Service*" OR "Employee Assistance Programs" OR " Youth Employment Program*" OR "Wage Subsidy" OR "Wage Subsidies" OR "Hiring Subsidy" OR "Hiring Subsidies " OR "Public Works" OR "Employment, Supported" OR "Assistance Program*, Employee" OR " Integrated Program*" OR "Community Integration" OR "Social Care*" OR "Child Support" OR "Child Grant" OR Daycare OR "Centre‐based childcare" OR "Center‐based childcare" OR "After School Clubs" OR "Child Care Service*" OR "Care for Children" OR "Parenting Education" OR "Education, Parenting" OR "Care for Children" OR Care for the Elderly OR "Home Care Services" OR "Home Health Nursing" OR "Foster Home Care" OR "Nurse Family Partnership" OR "Aid to families with dependent children" OR "Adult Protective Services" OR "Aid to the Totally Disabled" OR "Aid to Visually Impaired" OR "IPV Interventions" OR "Community‐based Program*" OR "Community Care" "Community‐based Intervention*" OR "Community‐based Health Insurance*" OR "Community‐based Project*" OR "Community Services" OR "Services, Community" OR "Community Service" OR "Service, Community") OR **AB** ("Labour Market Policy" OR "Labour Market Policies" OR "Activation Policies" OR "Active Labour Market Policies" OR "Labour Market Program*" OR "Labour Market Reform*" OR "Labour Standard*" OR "Labour Supply Program*" OR "Labor Market Policy" OR "Labor market Policies" OR "Labor Market Program*" OR "Labor Market Reform*" OR "Labor Supply Program*" OR "Labor Standard*" OR "Livelihood Support" OR "Livelihood Program*" OR "Livelihood Project" OR "Livelihood Empowerment" OR "Poverty Reduction Program*" OR "Poverty Alleviation Program*" OR "Skills Development" OR "Skill Development" OR "Vocational Training" OR "Job Training" OR "Training Support" OR "Entrepreneurship Program*" OR "Entrepreneurship Promotion" OR "Employment Program*" OR "Employment Scheme*" OR "Employment Service*" OR "Employee Assistance Programs" OR " Youth Employment Program*" OR "Wage Subsidy" OR "Wage Subsidies" OR "Hiring Subsidy" OR "Hiring Subsidies " OR "Public Works" OR "Employment, Supported" OR "Assistance Program*, Employee" OR " Integrated Program*" OR "Community Integration" OR "Social Care*" OR "Child Support" OR "Child Grant" OR Daycare OR "Centre‐based childcare" OR "Center‐based childcare" OR "After School Clubs" OR "Child Care Service*" OR "Care for Children" OR "Parenting Education" OR "Education, Parenting" OR "Care for Children" OR Care for the Elderly OR "Home Care Services" OR "Home Health Nursing" OR "Foster Home Care" OR "Nurse Family Partnership" OR "Aid to families with dependent children" OR "Adult Protective Services" OR "Aid to the Totally Disabled" OR "Aid to Visually Impaired" OR "IPV Interventions" OR "Community‐based Program*" OR "Community Care" "Community‐based Intervention*" OR "Community‐based Health Insurance*" OR "Community‐based Project*" OR "Community Services" OR "Services, Community" OR "Community Service" OR "Service, Community") OR **SU** ("Labour Market Policy" OR "Labour Market Policies" OR "Activation Policies" OR "Active Labour Market Policies" OR "Labour Market Program*" OR "Labour Market Reform*" OR "Labour Standard*" OR "Labour Supply Program*" OR "Labor Market Policy" OR "Labor market Policies" OR "Labor Market Program*" OR "Labor Market Reform*" OR "Labor Supply Program*" OR "Labor Standard*" OR "Livelihood Support" OR "Livelihood Program*" OR "Livelihood Project" OR "Livelihood Empowerment" OR "Poverty Reduction Program*" OR "Poverty Alleviation Program*" OR "Skills Development" OR "Skill Development" OR "Vocational Training" OR "Job Training" OR "Training Support" OR "Entrepreneurship Program*" OR "Entrepreneurship Promotion" OR "Employment Program*" OR "Employment Scheme*" OR "Employment Service*" OR "Employee Assistance Programs" OR " Youth Employment Program*" OR "Wage Subsidy" OR "Wage Subsidies" OR "Hiring Subsidy" OR "Hiring Subsidies " OR "Public Works" OR "Employment, Supported" OR "Assistance Program*, Employee" OR " Integrated Program*" OR "Community Integration" OR "Social Care*" OR "Child Support" OR "Child Grant" OR Daycare OR "Centre‐based childcare" OR "Center‐based childcare" OR "After School Clubs" OR "Child Care Service*" OR "Care for Children" OR "Parenting Education" OR "Education, Parenting" OR "Care for Children" OR Care for the Elderly OR "Home Care Services" OR "Home Health Nursing" OR "Foster Home Care" OR "Nurse Family Partnership" OR "Aid to families with dependent children" OR "Adult Protective Services" OR "Aid to the Totally Disabled" OR "Aid to Visually Impaired" OR "IPV Interventions" OR "Community‐based Program*" OR "Community Care" "Community‐based Intervention*" OR "Community‐based Health Insurance*" OR "Community‐based Project*" OR "Community Services" OR "Services, Community" OR "Community Service" OR "Service, Community")	67,608
S5	**TI** ("Social assistance" OR "Social Pension*" OR "Public Pension" OR "Pension*" OR "In‐kind Transfer*" OR "Conditional Cash Transfer" OR "Unconditional Cash Transfer" "Cash Plus Program*" OR "Social Transfer" OR "Financial Transfer" OR "Monetary Transfer" OR "Financial Incentive" OR "Economic Asset" OR "Financial Protection" OR "Resource Transfer" OR "Childcare Cash Benefits" OR "Birth Grant" OR "Paternity Benefit" OR "Maternity Benefit" OR "Parental Leave" OR "Paternity Leave" OR "Maternity Leave" OR " Birth Payment" OR "Family Support" OR "Income Maintenance" OR "Income Support" OR "Universal Basic Income" OR "minimum income guarantee" OR "Fee waiver*" OR "Food Voucher" OR "School Voucher*" OR "School Feeding" OR "Unemployment Benefit*" OR Housing Subsidy " or " Housing Subsidies " or " Public Housing " or " Housing for the Elderly " or " Disability Grant* " or " Disability Support " or " Tax Exemption " or " Old Age Assistance " or " Old Age Pension " or " Lunch Program* " or " Breakfast Program* " or " Food Security Program* " or " Food Stamp " or " Sick Leave " or " Disability Leave " or " Compensatory Support " or " Child Program* " or Nutritional Assistance" OR "Nutritional Support" OR "Social Protection*" OR "Social Policy" OR "Social Policies" OR "Policy, Social" OR "Policies, Social" OR "Protection, Social" OR Welfare, Social " or " Infant Welfare " or " Child Welfare " " Maternal Welfare " or " Welfare, Maternal " or " Unemployment Insurance " or " Insurance, Social " or " Insurance, Health " or " Medical Assistance " or " Medicaid " or " School Health Service* " or " School Health Program* " or " Ancillary School Service* " or " Security, Social " or Medicare or or " Household Insurance " or " Homemaker Services " or " Benefit, Retirement " or " Benefits, Retirement " or " Retirement Pension " or Social Insurance" OR "Death Benefit" OR "Annuities" OR "Social Service*" OR "Humanitarian Assistance" OR "Health Insurance" OR "National Insurance" OR "Micro Health Insurance" OR "Community‐based Health Insurance*" OR "Health Benefit" OR "Health Subsidies" OR " Insurance Benefit" OR "Benefit System" OR "Social Security" OR "Social Welfare" OR "Social grant" OR "Non‐contributory" OR "Contributory" OR "Cash Transfer*" OR "Social Safety Net") OR **AB** ("Social assistance" OR "Social Pension*" OR "Public Pension" OR "Pension*" OR "In‐kind Transfer*" OR "Conditional Cash Transfer" OR "Unconditional Cash Transfer" "Cash Plus Program*" OR "Social Transfer" OR "Financial Transfer" OR "Monetary Transfer" OR "Financial Incentive" OR "Economic Asset" OR "Financial Protection" OR "Resource Transfer" OR "Childcare Cash Benefits" OR "Birth Grant" OR "Paternity Benefit" OR "Maternity Benefit" OR "Parental Leave" OR "Paternity Leave" OR "Maternity Leave" OR " Birth Payment" OR "Family Support" OR "Income Maintenance" OR "Income Support" OR "Universal Basic Income" OR "minimum income guarantee" OR "Fee waiver*" OR "Food Voucher" OR "School Voucher*" OR "School Feeding" OR "Unemployment Benefit*" OR Housing Subsidy " or " Housing Subsidies " or " Public Housing " or " Housing for the Elderly " or " Disability Grant* " or " Disability Support " or " Tax Exemption " or " Old Age Assistance " or " Old Age Pension " or " Lunch Program* " or " Breakfast Program* " or " Food Security Program* " or " Food Stamp " or " Sick Leave " or " Disability Leave " or " Compensatory Support " or " Child Program* " or Nutritional Assistance" OR "Nutritional Support" OR "Social Protection*" OR "Social Policy" OR "Social Policies" OR "Policy, Social" OR "Policies, Social" OR "Protection, Social" OR Welfare, Social " or " Infant Welfare " or " Child Welfare " " Maternal Welfare " or " Welfare, Maternal " or " Unemployment Insurance " or " Insurance, Social " or " Insurance, Health " or " Medical Assistance " or " Medicaid " or " School Health Service* " or " School Health Program* " or " Ancillary School Service* " or " Security, Social " or Medicare or or " Household Insurance " or " Homemaker Services " or " Benefit, Retirement " or " Benefits, Retirement " or " Retirement Pension " or Social Insurance" OR "Death Benefit" OR "Annuities" OR "Social Service*" OR "Humanitarian Assistance" OR "Health Insurance" OR "National Insurance" OR "Micro Health Insurance" OR "Community‐based Health Insurance*" OR "Health Benefit" OR "Health Subsidies" OR " Insurance Benefit" OR "Benefit System" OR "Social Security" OR "Social Welfare" OR "Social grant" OR "Non‐contributory" OR "Contributory" OR "Cash Transfer*" OR "Social Safety Net") OR **SU** ("Social assistance" OR "Social Pension*" OR "Public Pension" OR "Pension*" OR "In‐kind Transfer*" OR "Conditional Cash Transfer" OR "Unconditional Cash Transfer" "Cash Plus Program*" OR "Social Transfer" OR "Financial Transfer" OR "Monetary Transfer" OR "Financial Incentive" OR "Economic Asset" OR "Financial Protection" OR "Resource Transfer" OR "Childcare Cash Benefits" OR "Birth Grant" OR "Paternity Benefit" OR "Maternity Benefit" OR "Parental Leave" OR "Paternity Leave" OR "Maternity Leave" OR " Birth Payment" OR "Family Support" OR "Income Maintenance" OR "Income Support" OR "Universal Basic Income" OR "minimum income guarantee" OR "Fee waiver*" OR "Food Voucher" OR "School Voucher*" OR "School Feeding" OR "Unemployment Benefit*" OR Housing Subsidy " or " Housing Subsidies " or " Public Housing " or " Housing for the Elderly " or " Disability Grant* " or " Disability Support " or " Tax Exemption " or " Old Age Assistance " or " Old Age Pension " or " Lunch Program* " or " Breakfast Program* " or " Food Security Program* " or " Food Stamp " or " Sick Leave " or " Disability Leave " or " Compensatory Support " or " Child Program* " or Nutritional Assistance" OR "Nutritional Support" OR "Social Protection*" OR "Social Policy" OR "Social Policies" OR "Policy, Social" OR "Policies, Social" OR "Protection, Social" OR Welfare, Social " or " Infant Welfare " or " Child Welfare " " Maternal Welfare " or " Welfare, Maternal " or " Unemployment Insurance " or " Insurance, Social " or " Insurance, Health " or " Medical Assistance " or " Medicaid " or " School Health Service* " or " School Health Program* " or " Ancillary School Service* " or " Security, Social " or Medicare or or " Household Insurance " or " Homemaker Services " or " Benefit, Retirement " or " Benefits, Retirement " or " Retirement Pension " or Social Insurance" OR "Death Benefit" OR "Annuities" OR "Social Service*" OR "Humanitarian Assistance" OR "Health Insurance" OR "National Insurance" OR "Micro Health Insurance" OR "Community‐based Health Insurance*" OR "Health Benefit" OR "Health Subsidies" OR " Insurance Benefit" OR "Benefit System" OR "Social Security" OR "Social Welfare" OR "Social grant" OR "Non‐contributory" OR "Contributory" OR "Cash Transfer*" OR "Social Safety Net")	20,607
S4	S1 OR S2 OR S3	143,551
S3	**TI** (afghanistan OR albania OR algeria OR "american samoa" OR angola OR antigua OR barbuda OR argentina OR armenia OR armenian OR aruba OR azerbaijan OR bahrain OR bangladesh OR barbados OR belarus OR byelarus OR belorussia OR byelorussian OR belize OR "british honduras" OR benin OR dahomey OR bhutan OR bolivia OR bosnia OR herzegovina OR Botswana OR bechuanaland OR brazil OR brasil OR bulgaria OR "burkina faso" OR "burkina fasso" OR "upper volta" OR burundi OR urundi OR "cabo verde" OR "cape verde" OR cambodia OR kampuchea OR "khmer republic" OR cameroon OR cameron OR cameroun OR "central african republic" OR "ubangi shari" OR chad OR chile OR china OR colombia OR comoros OR "comoro islands" OR mayotte OR congo OR zaire OR "costa rica" OR "cote d'ivoire" OR "cote d'ivoire" OR "cote d'ivoire" OR "ivory coast" OR croatia OR cuba OR cyprus OR "czech republic" OR czechoslovakia OR djibouti OR "french somaliland" OR dominica OR "dominican republic" OR ecuador OR egypt OR "united arab republic" OR "el salvador" OR "equatorial guinea" OR "spanish guinea" OR eritrea OR estonia OR eswatini OR swaziland OR ethiopia OR fiji OR gabon OR "gabonese republic" OR gambia OR georgia OR Georgian OR ghana OR "gold coast" OR gibraltar OR greece OR grenada OR guam OR guatemala OR guinea OR guyana OR guiana OR haiti OR hispaniola OR honduras OR hungary OR india OR indonesia OR timor OR iran OR iraq OR "isle of man" OR jamaica OR jordan OR kazakhstan OR kazakh OR kenya OR korea OR kosovo OR kyrgyzstan OR kirghizia OR kirgizstan OR "kyrgyz republic" OR kirghiz OR laos OR "lao pdr" OR "lao people's democratic republic" OR latvia OR lebanon OR lesotho OR basutoland OR liberia OR libya OR "libyan arab jamahiriya" OR lithuania OR macau OR macao OR macedonia OR madagascar OR "malagasy republic" OR malawi OR nyasaland OR malaysia OR maldives OR "indian ocean" OR mali OR malta OR micronesia OR kiribati OR "marshall islands" OR nauru OR "northern mariana islands" OR palau OR tuvalu OR mauritania OR mauritius OR mexico OR moldova OR moldovian OR mongolia OR montenegro OR morocco OR ifni OR mozambique OR "portuguese east africa" OR myanmar OR burma OR namibia OR nepal OR "netherlands antilles" OR nicaragua OR niger OR nigeria OR oman OR muscat OR pakistan OR panama OR "papua new guinea" OR paraguay OR peru OR philippines OR philipines OR phillipines OR phillippines OR poland OR "polish people's republic" OR portugal OR "portuguese republic" OR "puerto rico" OR romania OR russia OR "russian federation" OR ussr OR "soviet union" OR "union of soviet socialist republics" OR rwanda OR ruanda OR samoa OR "pacific islands" OR polynesia OR "samoan islands" OR "sao tome" and principe OR "saudi arabia" OR senegal OR serbia OR seychelles OR "sierra leone" OR slovakia OR "slovak republic" OR slovenia OR melanesia OR "solomon island" OR "solomon islands" OR "norfolk island" OR somalia OR "south africa" OR "south sudan" OR "sri lanka" OR ceylon OR "saint kitts" OR nevis OR "st kitts" OR nevis OR "saint lucia" OR "st lucia" OR "saint vincent" OR "st vincent" OR grenadines OR sudan OR suriname OR surinam OR syria OR "syrian OR muscat OR pakistan OR panama OR "papua new guinea" OR paraguay OR peru OR philippines OR philipines OR phillipines OR phillippines OR poland OR "polish people's republic" OR portugal OR "portuguese republic" OR "puerto rico" OR romania OR russia OR "russian federation" OR ussr OR "soviet union" OR "union of soviet socialist republics" OR rwanda OR ruanda OR samoa OR "pacific islands" OR polynesia OR "samoan islands" OR "sao tome" and principe OR "saudi arabia" OR senegal OR serbia OR seychelles OR "sierra leone" OR slovakia OR "slovak republic" OR slovenia OR melanesia OR "solomon island" OR "solomon islands" OR "norfolk island" OR somalia OR "south africa" OR "south sudan" OR "sri lanka" OR ceylon OR "saint kitts" OR nevis OR "st kitts" OR nevis OR "saint lucia" OR "st lucia" OR "saint vincent" OR "st vincent" OR grenadines OR sudan OR suriname OR surinam OR syria OR "syrian arab republic" OR tajikistan OR tadjikistan OR tadzhikistan OR tadzhik OR tanzania OR Tanganyika OR thailand OR siam OR "timor leste" OR "east timor" OR togo OR "togolese republic" OR tonga OR trinidad OR tobago OR tunisia OR turkey OR turkmenistan OR turkmen OR uganda OR ukraine OR uruguay OR uzbekistan OR uzbek OR vanuatu OR "new hebrides" OR venezuela OR vietnam OR "viet name" OR "west bank" OR gaza OR palestine OR yemen OR yugoslavia OR zambia OR zimbabwe OR "northern rhodesia") OR **SU** (afghanistan OR albania OR algeria OR "american samoa" OR angola OR antigua OR barbuda OR argentina OR armenia OR armenian OR aruba OR azerbaijan OR bahrain OR bangladesh OR barbados OR belarus OR byelarus OR belorussia OR byelorussian OR belize OR "british honduras" OR benin OR dahomey OR bhutan OR bolivia OR bosnia OR herzegovina OR Botswana OR bechuanaland OR brazil OR brasil OR bulgaria OR "burkina faso" OR "burkina fasso" OR "upper volta" OR burundi OR urundi OR "cabo verde" OR "cape verde" OR cambodia OR kampuchea OR "khmer republic" OR cameroon OR cameron OR cameroun OR "central african republic" OR "ubangi shari" OR chad OR chile OR china OR colombia OR comoros OR "comoro islands" OR mayotte OR congo OR zaire OR "costa rica" OR "cote d'ivoire" OR "cote d'ivoire" OR "cote d'ivoire" OR "ivory coast" OR croatia OR cuba OR cyprus OR "czech republic" OR czechoslovakia OR djibouti OR "french somaliland" OR dominica OR "dominican republic" OR ecuador OR egypt OR "united arab republic" OR "el salvador" OR "equatorial guinea" OR "spanish guinea" OR eritrea OR estonia OR eswatini OR swaziland OR ethiopia OR fiji OR gabon OR "gabonese republic" OR gambia OR georgia OR Georgian OR ghana OR "gold coast" OR gibraltar OR greece OR grenada OR guam OR guatemala OR guinea OR guyana OR guiana OR haiti OR hispaniola OR honduras OR hungary OR india OR indonesia OR timor OR iran OR iraq OR "isle of man" OR jamaica OR jordan OR kazakhstan OR kazakh OR kenya OR korea OR kosovo OR kyrgyzstan OR kirghizia OR kirgizstan OR "kyrgyz republic" OR kirghiz OR laos OR "lao pdr" OR "lao people's democratic republic" OR latvia OR lebanon OR lesotho OR basutoland OR liberia OR libya OR "libyan arab jamahiriya" OR lithuania OR macau OR macao OR macedonia OR madagascar OR "malagasy republic" OR malawi OR nyasaland OR malaysia OR maldives OR "indian ocean" OR mali OR malta OR micronesia OR kiribati OR "marshall islands" OR nauru OR "northern mariana islands" OR palau OR tuvalu OR mauritania OR mauritius OR mexico OR moldova OR moldovian OR mongolia OR montenegro OR morocco OR ifni OR mozambique OR "portuguese east africa" OR myanmar OR burma OR namibia OR nepal OR "netherlands antilles" OR nicaragua OR niger OR nigeria OR oman OR muscat OR pakistan OR panama OR "papua new guinea" OR paraguay OR peru OR philippines OR philipines OR phillipines OR phillippines OR poland OR "polish people's republic" OR portugal OR "portuguese republic" OR "puerto rico" OR romania OR russia OR "russian federation" OR ussr OR "soviet union" OR "union of soviet socialist republics" OR rwanda OR ruanda OR samoa OR "pacific islands" OR polynesia OR "samoan islands" OR "sao tome" and principe OR "saudi arabia" OR senegal OR serbia OR seychelles OR "sierra leone" OR slovakia OR "slovak republic" OR slovenia OR melanesia OR "solomon island" OR "solomon islands" OR "norfolk island" OR somalia OR "south africa" OR "south sudan" OR "sri lanka" OR ceylon OR "saint kitts" OR nevis OR "st kitts" OR nevis OR "saint lucia" OR "st lucia" OR "saint vincent" OR "st vincent" OR grenadines OR sudan OR suriname OR surinam OR syria OR "syrian OR muscat OR pakistan OR panama OR "papua new guinea" OR paraguay OR peru OR philippines OR philipines OR phillipines OR phillippines OR poland OR "polish people's republic" OR portugal OR "portuguese republic" OR "puerto rico" OR romania OR russia OR "russian federation" OR ussr OR "soviet union" OR "union of soviet socialist republics" OR rwanda OR ruanda OR samoa OR "pacific islands" OR polynesia OR "samoan islands" OR "sao tome" and principe OR "saudi arabia" OR senegal OR serbia OR seychelles OR "sierra leone" OR slovakia OR "slovak republic" OR slovenia OR melanesia OR "solomon island" OR "solomon islands" OR "norfolk island" OR somalia OR "south africa" OR "south sudan" OR "sri lanka" OR ceylon OR "saint kitts" OR nevis OR "st kitts" OR nevis OR "saint lucia" OR "st lucia" OR "saint vincent" OR "st vincent" OR grenadines OR sudan OR suriname OR surinam OR syria OR "syrian arab republic" OR tajikistan OR tadjikistan OR tadzhikistan OR tadzhik OR tanzania OR Tanganyika OR thailand OR siam OR "timor leste" OR "east timor" OR togo OR "togolese republic" OR tonga OR trinidad OR tobago OR tunisia OR turkey OR turkmenistan OR turkmen OR uganda OR ukraine OR uruguay OR uzbekistan OR uzbek OR vanuatu OR "new hebrides" OR venezuela OR vietnam OR "viet name" OR "west bank" OR gaza OR palestine OR yemen OR yugoslavia OR zambia OR zimbabwe OR "northern rhodesia") OR **AB** (afghanistan OR albania OR algeria OR "american samoa" OR angola OR antigua OR barbuda OR argentina OR armenia OR armenian OR aruba OR azerbaijan OR bahrain OR bangladesh OR barbados OR belarus OR byelarus OR belorussia OR byelorussian OR belize OR "british honduras" OR benin OR dahomey OR bhutan OR bolivia OR bosnia OR herzegovina OR Botswana OR bechuanaland OR brazil OR brasil OR bulgaria OR "burkina faso" OR "burkina fasso" OR "upper volta" OR burundi OR urundi OR "cabo verde" OR "cape verde" OR cambodia OR kampuchea OR "khmer republic" OR cameroon OR cameron OR cameroun OR "central african republic" OR "ubangi shari" OR chad OR chile OR china OR colombia OR comoros OR "comoro islands" OR mayotte OR congo OR zaire OR "costa rica" OR "cote d'ivoire" OR "cote d'ivoire" OR "cote d'ivoire" OR "ivory coast" OR croatia OR cuba OR cyprus OR "czech republic" OR czechoslovakia OR djibouti OR "french somaliland" OR dominica OR "dominican republic" OR ecuador OR egypt OR "united arab republic" OR "el salvador" OR "equatorial guinea" OR "spanish guinea" OR eritrea OR estonia OR eswatini OR swaziland OR ethiopia OR fiji OR gabon OR "gabonese republic" OR gambia OR georgia OR Georgian OR ghana OR "gold coast" OR gibraltar OR greece OR grenada OR guam OR guatemala OR guinea OR guyana OR guiana OR haiti OR hispaniola OR honduras OR hungary OR india OR indonesia OR timor OR iran OR iraq OR "isle of man" OR jamaica OR jordan OR kazakhstan OR kazakh OR kenya OR korea OR kosovo OR kyrgyzstan OR kirghizia OR kirgizstan OR "kyrgyz republic" OR kirghiz OR laos OR "lao pdr" OR "lao people's democratic republic" OR latvia OR lebanon OR lesotho OR basutoland OR liberia OR libya OR "libyan arab jamahiriya" OR lithuania OR macau OR macao OR macedonia OR madagascar OR "malagasy republic" OR malawi OR nyasaland OR malaysia OR maldives OR "indian ocean" OR mali OR malta OR micronesia OR kiribati OR "marshall islands" OR nauru OR "northern mariana islands" OR palau OR tuvalu OR mauritania OR mauritius OR mexico OR moldova OR moldovian OR mongolia OR montenegro OR morocco OR ifni OR mozambique OR "portuguese east africa" OR myanmar OR burma OR namibia OR nepal OR "netherlands antilles" OR nicaragua OR niger OR nigeria OR oman OR muscat OR pakistan OR panama OR "papua new guinea" OR paraguay OR peru OR philippines OR philipines OR phillipines OR phillippines OR poland OR "polish people's republic" OR portugal OR "portuguese republic" OR "puerto rico" OR romania OR russia OR "russian federation" OR ussr OR "soviet union" OR "union of soviet socialist republics" OR rwanda OR ruanda OR samoa OR "pacific islands" OR polynesia OR "samoan islands" OR "sao tome" and principe OR "saudi arabia" OR senegal OR serbia OR seychelles OR "sierra leone" OR slovakia OR "slovak republic" OR slovenia OR melanesia OR "solomon island" OR "solomon islands" OR "norfolk island" OR somalia OR "south africa" OR "south sudan" OR "sri lanka" OR ceylon OR "saint kitts" OR nevis OR "st kitts" OR nevis OR "saint lucia" OR "st lucia" OR "saint vincent" OR "st vincent" OR grenadines OR sudan OR suriname OR surinam OR syria OR "syrian OR muscat OR pakistan OR panama OR "papua new guinea" OR paraguay OR peru OR philippines OR philipines OR phillipines OR phillippines OR poland OR "polish people's republic" OR portugal OR "portuguese republic" OR "puerto rico" OR romania OR russia OR "russian federation" OR ussr OR "soviet union" OR "union of soviet socialist republics" OR rwanda OR ruanda OR samoa OR "pacific islands" OR polynesia OR "samoan islands" OR "sao tome" and principe OR "saudi arabia" OR senegal OR serbia OR seychelles OR "sierra leone" OR slovakia OR "slovak republic" OR slovenia OR melanesia OR "solomon island" OR "solomon islands" OR "norfolk island" OR somalia OR "south africa" OR "south sudan" OR "sri lanka" OR ceylon OR "saint kitts" OR nevis OR "st kitts" OR nevis OR "saint lucia" OR "st lucia" OR "saint vincent" OR "st vincent" OR grenadines OR sudan OR suriname OR surinam OR syria OR "syrian arab republic" OR tajikistan OR tadjikistan OR tadzhikistan OR tadzhik OR tanzania OR Tanganyika OR thailand OR siam OR "timor leste" OR "east timor" OR togo OR "togolese republic" OR tonga OR trinidad OR tobago OR tunisia OR turkey OR turkmenistan OR turkmen OR uganda OR ukraine OR uruguay OR uzbekistan OR uzbek OR vanuatu OR "new hebrides" OR venezuela OR vietnam OR "viet name" OR "west bank" OR gaza OR palestine OR yemen OR yugoslavia OR zambia OR zimbabwe OR "northern rhodesia")	125,298
S2	**TI** ((developing OR (less* N1 developed) OR "under developed" OR underdeveloped OR "under served" OR underserved OR deprived OR poor* OR "middle income" OR (low* N1 income)) N1 (countr* OR nation* OR population* OR world)) OR **AB** ((developing OR (less* N1 developed) OR "under developed" OR underdeveloped OR "under served" OR underserved OR deprived OR poor* OR "middle income" OR (low* N1 income)) N1 (countr* OR nation* or population* OR world)) OR **SU**((developing OR (less* N1 developed) OR "under developed" OR underdeveloped OR "under served" OR underserved OR deprived OR poor* OR "middle income" OR (low* N1 income)) N1 (countr* OR nation* or population* OR world)) OR **TI**((developing OR (less* N1 developed) OR "under developed" OR “middle income" OR (low* N1 income)) N1 (economy OR economies)) OR **AB** (developing OR (less* N1 developed) OR "under developed" OR "middle income" OR (low* N1 income)) N1 (economy OR economies)) OR **SU**((developing OR (less* N1 developed) OR "under developed" OR "middle income" OR (low* N1 income) N1 (economy OR economies)) OR **TI**(low N3 middle N3 countr*) OR **AB**(low N3 middle N3 countr*) OR **SU**(low N3 middle N3 countr*) OR **TI**(low* N1 (gdp OR gnp OR "gross domestic" OR "gross national")) OR **AB** (low* N1 (gdp OR gnp OR "gross domestic" OR "gross national")) OR **SU** (low* N1 (gdp OR gnp OR "gross domestic" OR "gross national")) OR **TI**(lmic OR lmics OR "third world" OR "lami country" OR "lami countries") OR **AB** (lmic OR lmics OR "third world" OR "lami country" OR "lami countries") OR **SU**(lmic OR lmics OR "third world" OR "lami country" OR "lami countries") OR **TI**("transitional country" OR "transitional countries") OR **AB**("transitional country" OR "transitional countries") OR **SU**("transitional country" OR "transitional countries") OR **TI** (emerging N1 (economy or economies OR nation*) OR **AB** (emerging N1 (economy OR economies OR nation*) OR **SU** (emerging N1 (economy OR economies OR nation*)	20,070
S1	**TI** ("west indies" OR "indian ocean islands" OR caribbean OR "central america" OR "latin america" OR "south america" OR magreb OR maghrib OR "central asia" OR "north asia" OR "northern asia" OR "southeastern asia" OR "south eastern asia" OR "southeast asia" OR "south east asia" OR "western asia" OR "east europe" OR "eastern europe") OR **AB**("west indies" OR "indian ocean islands" OR caribbean OR "central america" OR "latin america" OR "south america" OR magreb OR maghrib OR "central asia" OR "north asia" OR "northern asia" OR "southeastern asia" OR "south eastern asia" OR "southeast asia" OR "south east asia" OR "western asia" OR "east europe" OR "eastern europe") OR **SU**("west indies" OR "indian ocean islands" OR caribbean OR "central america" OR "latin america" OR "south america" OR magreb OR maghrib OR "central asia" OR "north asia" OR "northern asia" OR "southeastern asia" OR "south eastern asia" OR "southeast asia" OR "south east asia" OR "western asia" OR "east europe" OR "eastern europe") OR **TI(**africa OR asia OR caribbean OR "west indies" OR "south america" OR "latin america" OR "central america" OR "middle east" OR "global south") OR **AB**(africa OR asia OR caribbean OR "west indies" OR "south america" OR "latin america" OR "central america" OR "middle east" OR "global south") OR **SU** (africa OR asia OR caribbean OR "west indies" OR "south america" OR "latin america" OR "central america" OR "middle east" OR "global south")	25,373

## APPENDIX E INTERNATIONAL BIBLIOGRAPHY OF THE SOCIAL SCIENCE DATABASE

Database name: International Bibliography of the Social Science Database

Date of search: 23/02/2021

Number of results: 39

**Table:**

#	Searched for	Results
S1	TI,AB,SU("Social assistance" OR "Social Pension*" OR "Public Pension" OR "Pension*" OR "In‐kind Transfer*" OR "Conditional Cash Transfer" OR "Unconditional Cash Transfer" "Cash Plus Program*" OR "Social Transfer" OR "Financial Transfer" OR "Monetary Transfer" OR "Financial Incentive" OR "Economic Asset" OR "Financial Protection" OR "Resource Transfer" OR "Childcare Cash Benefits" OR "Birth Grant" OR "Paternity Benefit" OR "Maternity Benefit" OR "Parental Leave" OR "Paternity Leave" OR "Maternity Leave" OR " Birth Payment" OR "Family Support" OR "Income Maintenance" OR "Income Support" OR "Universal Basic Income" OR "minimum income guarantee" OR "Fee waiver*" OR "Food Voucher" OR "School Voucher*" OR "School Feeding" OR "Unemployment Benefit*" OR Housing Subsidy " or " Housing Subsidies " or " Public Housing " or " Housing for the Elderly " or " Disability Grant* " or " Disability Support " or " Tax Exemption " or " Old Age Assistance " or " Old Age Pension " or " Lunch Program* " or " Breakfast Program* " or " Food Security Program* " or " Food Stamp " or " Sick Leave " or " Disability Leave " or " Compensatory Support " or " Child Program* " or Nutritional Assistance" OR "Nutritional Support" OR "Social Protection*" OR "Social Policy" OR "Social Policies" OR "Policy, Social" OR "Policies, Social" OR "Protection, Social" OR Welfare, Social " or " Infant Welfare " or " Child Welfare " " Maternal Welfare " or " Welfare, Maternal " or " Unemployment Insurance " or " Insurance, Social " or " Insurance, Health " or " Medical Assistance " or " Medicaid " or " School Health Service* " or " School Health Program* " or " Ancillary School Service* " or " Security, Social " or Medicare or or " Household Insurance " or " Homemaker Services " or " Benefit, Retirement " or " Benefits, Retirement " or " Retirement Pension " or Social Insurance" OR "Death Benefit" OR "Annuities" OR "Social Service*" OR "Humanitarian Assistance" OR "Health Insurance" OR "National Insurance" OR "Micro Health Insurance" OR "Community‐based Health Insurance*" OR "Health Benefit" OR "Health Subsidies" OR " Insurance Benefit" OR "Benefit System" OR "Social Security" OR "Social Welfare" OR "Social grant" OR "Non‐contributory" OR "Contributory" OR "Cash Transfer*" OR "Social Safety Net") AND pd(20100101‐20210301)	42,832
S2	TI,AB,SU("Labour Market Policy" OR "Labour Market Policies" OR "Activation Policies" OR "Active Labour Market Policies" OR "Labour Market Program*" OR "Labour Market Reform*" OR "Labour Standard*" OR "Labour Supply Program*" OR "Labor Market Policy" OR "Labor market Policies" OR "Labor Market Program*" OR "Labor Market Reform*" OR "Labor Supply Program*" OR "Labor Standard*" OR "Livelihood Support" OR "Livelihood Program*" OR "Livelihood Project" OR "Livelihood Empowerment" OR "Poverty Reduction Program*" OR "Poverty Alleviation Program*" OR "Skills Development" OR "Skill Development" OR "Vocational Training" OR "Job Training" OR "Training Support" OR "Entrepreneurship Program*" OR "Entrepreneurship Promotion" OR "Employment Program*" OR "Employment Scheme*" OR "Employment Service*" OR "Employee Assistance Programs" OR " Youth Employment Program*" OR "Wage Subsidy" OR "Wage Subsidies" OR "Hiring Subsidy" OR "Hiring Subsidies " OR "Public Works" OR "Employment, Supported" OR "Assistance Program*, Employee" OR " Integrated Program*" OR "Community Integration" OR "Social Care*" OR "Child Support" OR "Child Grant" OR Daycare OR "Centre‐based childcare" OR "Center‐based childcare" OR "After School Clubs" OR "Child Care Service*" OR "Care for Children" OR "Parenting Education" OR "Education, Parenting" OR "Care for Children" OR Care for the Elderly OR "Home Care Services" OR "Home Health Nursing" OR "Foster Home Care" OR "Nurse Family Partnership" OR "Aid to families with dependent children" OR "Adult Protective Services" OR "Aid to the Totally Disabled" OR "Aid to Visually Impaired" OR "IPV Interventions" OR "Community‐based Program*" OR "Community Care" "Community‐based Intervention*" OR "Community‐based Health Insurance*" OR "Community‐based Project*" OR "Community Services" OR "Services, Community" OR "Community Service" OR "Service, Community") AND pd(20100101‐20210301)	12,109
S3	((TI, AB, SU("meta regression" OR "meta synth*" OR "meta‐synth*" OR"meta analy*" OR "metaanaly*" OR "meta‐analy*" OR "metanaly*" OR "metaregression" OR "meta‐regression" OR "methodologic* overview" OR "pool* analys*" OR "pool* data" OR "quantitative* overview" OR "research integration")) OR (TI, AB, SU((systematic* OR synthes*) N4 (research OR evaluation* OR finding* OR thematic* OR report OR descriptive OR explanatory OR narrative OR meta* OR review* OR data OR literature OR studies OR evidence OR map OR quantitative OR study OR studies OR paper OR impact OR impacts OR effect* OR compar*))) AND (TI, AB, SU(review N4 (effectiveness OR effects OR systemat* OR synth* OR integrat* OR map* OR methodologic* OR quantitative OR evidence OR literature)))) AND pd(20100101‐20210301)	83
S4	(TI, AB, SU(women OR "women's right" OR "young women" OR woman* OR adolescent* OR girl* OR schoolgirl* OR "school girl*" OR female* OR man OR men OR "young men" OR boy* OR schoolboy* OR "school boy*" OR teen* OR youth* OR young* OR mother* OR father* OR parent* OR caregiver)) OR (TI, AB, SU(femini* OR masculi* OR female*OR male* OR gender* OR "social equality" OR "social equity" OR empower* OR "equal opportunit*" OR egalitarian*)) AND pd(20100101‐20210301)	812
S5	TI("west indies" OR "indian ocean islands" OR caribbean OR "central america" OR "latin america" OR "south america" OR "central asia" OR "north asia" OR "northern asia" OR "southeastern asia" OR "south eastern asia" OR "southeast asia" OR "south east asia" OR "western asia" OR "east europe" OR "eastern europe" OR "developing country" OR "developing countries" OR "developing nation" OR "developing nations" OR "developing population" OR "developing populations" OR "developing world" OR "less developed country" OR "less developed countries" OR "less developed nation" OR "less developed nations" OR "less developed world" OR "lesser developed countries" OR "lesser developed nations" OR "under developed country" OR "under developed countries" OR "under developed nations" OR "under developed world" OR "underdeveloped country" OR "underdeveloped countries" OR "underdeveloped nation" OR "underdeveloped nations" OR "underdeveloped population" OR "underdeveloped populations" OR "underdeveloped world" OR "middle income country" OR "middle income countries" OR "middle income nation" OR "middle income nations" OR "middle income population" OR "middle income populations" OR "low income country" OR "low income countries" OR "low income nation" OR "low income nations" OR "low income population" OR "low income populations" OR "lower income country" OR "lower income countries" OR "lower income nations" OR "lower income population" OR "lower income populations" OR "underserved countries" OR "underserved nations" OR "underserved population" OR "underserved populations" OR "under served population" OR "under served populations" OR "deprived countries" OR "deprived population" OR "deprived populations" OR "poor country" OR "poor countries" OR "poor nation" OR "poor nations" OR "poor population" OR "poor populations" OR "poor world" OR "poorer countries" OR "poorer nations" OR "poorer population" OR "poorer populations" OR "developing economy" OR "developing economies" OR "less developed economy" OR "less developed economies" OR "underdeveloped economies" OR "middle income economy" OR "middle income economies" OR "low income economy" OR "low income economies" OR "lower income economies " OR "low gdp" OR "low gnp" OR "low gross domestic" OR "low gross national" OR "lower gdp" OR "lower gross domestic" OR lmic OR lmics OR "third world" OR "lami country" OR "lami countries" OR "transitional country" OR "transitional countries" OR "emerging economies " OR "emerging nation" OR "emerging nations" Africa OR "south america" OR "latin america" OR "central america" OR "middle east" OR "global south") AND pd(20100101‐20210301)	10,388
S6	AB("west indies" OR "indian ocean islands" OR caribbean OR "central america" OR "latin america" OR "south america" OR "central asia" OR "north asia" OR "northern asia" OR "southeastern asia" OR "south eastern asia" OR "southeast asia" OR "south east asia" OR "western asia" OR "east europe" OR "eastern europe" OR "developing country" OR "developing countries" OR "developing nation" OR "developing nations" OR "developing population" OR "developing populations" OR "developing world" OR "less developed country" OR "less developed countries" OR "less developed nation" OR "less developed nations" OR "less developed world" OR "lesser developed countries" OR "lesser developed nations" OR "under developed country" OR "under developed countries" OR "under developed nations" OR "under developed world" OR "underdeveloped country" OR "underdeveloped countries" OR "underdeveloped nation" OR "underdeveloped nations" OR "underdeveloped population" OR "underdeveloped populations" OR "underdeveloped world" OR "middle income country" OR "middle income countries" OR "middle income nation" OR "middle income nations" OR "middle income population" OR "middle income populations" OR "low income country" OR "low income countries" OR "low income nation" OR "low income nations" OR "low income population" OR "low income populations" OR "lower income country" OR "lower income countries" OR "lower income nations" OR "lower income population" OR "lower income populations" OR "underserved countries" OR "underserved nations" OR "underserved population" OR "underserved populations" OR "under served population" OR "under served populations" OR "deprived countries" OR "deprived population" OR "deprived populations" OR "poor country" OR "poor countries" OR "poor nation" OR "poor nations" OR "poor population" OR "poor populations" OR "poor world" OR "poorer countries" OR "poorer nations" OR "poorer population" OR "poorer populations" OR "developing economy" OR "developing economies" OR "less developed economy" OR "less developed economies" OR "underdeveloped economies" OR "middle income economy" OR "middle income economies" OR "low income economy" OR "low income economies" OR "lower income economies " OR "low gdp" OR "low gnp" OR "low gross domestic" OR "low gross national" OR "lower gdp" OR "lower gross domestic" OR lmic OR lmics OR "third world" OR "lami country" OR "lami countries" OR "transitional country" OR "transitional countries" OR "emerging economies " OR "emerging nation" OR "emerging nations" Africa OR "south america" OR "latin america" OR "central america" OR "middle east" OR "global south") AND pd(20100101‐20210301)	25,852
S7	SU("west indies" OR "indian ocean islands" OR caribbean OR "central america" OR "latin america" OR "south america" OR "central asia" OR "north asia" OR "northern asia" OR "southeastern asia" OR "south eastern asia" OR "southeast asia" OR "south east asia" OR "western asia" OR "east europe" OR "eastern europe" OR "developing country" OR "developing countries" OR "developing nation" OR "developing nations" OR "developing population" OR "developing populations" OR "developing world" OR "less developed country" OR "less developed countries" OR "less developed nation" OR "less developed nations" OR "less developed world" OR "lesser developed countries" OR "lesser developed nations" OR "under developed country" OR "under developed countries" OR "under developed nations" OR "under developed world" OR "underdeveloped country" OR "underdeveloped countries" OR "underdeveloped nation" OR "underdeveloped nations" OR "underdeveloped population" OR "underdeveloped populations" OR "underdeveloped world" OR "middle income country" OR "middle income countries" OR "middle income nation" OR "middle income nations" OR "middle income population" OR "middle income populations" OR "low income country" OR "low income countries" OR "low income nation" OR "low income nations" OR "low income population" OR "low income populations" OR "lower income country" OR "lower income countries" OR "lower income nations" OR "lower income population" OR "lower income populations" OR "underserved countries" OR "underserved nations" OR "underserved population" OR "underserved populations" OR "under served population" OR "under served populations" OR "deprived countries" OR "deprived population" OR "deprived populations" OR "poor country" OR "poor countries" OR "poor nation" OR "poor nations" OR "poor population" OR "poor populations" OR "poor world" OR "poorer countries" OR "poorer nations" OR "poorer population" OR "poorer populations" OR "developing economy" OR "developing economies" OR "less developed economy" OR "less developed economies" OR "underdeveloped economies" OR "middle income economy" OR "middle income economies" OR "low income economy" OR "low income economies" OR "lower income economies " OR "low gdp" OR "low gnp" OR "low gross domestic" OR "low gross national" OR "lower gdp" OR "lower gross domestic" OR lmic OR lmics OR "third world" OR "lami country" OR "lami countries" OR "transitional country" OR "transitional countries" OR "emerging economies " OR "emerging nation" OR "emerging nations" Africa OR "south america" OR "latin america" OR "central america" OR "middle east" OR "global south") AND pd(20100101‐20210301)	37,063
S8	TI(afghanistan OR albania OR algeria OR "american samoa" OR angola OR antigua OR barbuda OR argentina OR armenia OR armenian OR aruba OR azerbaijan OR bahrain OR bangladesh OR barbados OR belarus OR byelarus OR belorussia OR byelorussian OR belize OR "british honduras" OR benin OR dahomey OR bhutan OR bolivia OR bosnia OR herzegovina OR botswana OR bechuanaland OR brazil OR brasil OR bulgaria OR "burkina faso" OR "burkina fasso" OR "upper volta" OR burundi OR urundi OR "cabo verde" OR "cape verde" OR cambodia OR kampuchea OR "khmer republic" OR cameroon OR cameron OR cameroun OR "central african republic" OR "ubangi shari" OR chad OR chile OR china OR colombia OR comoros OR "comoro islands" OR mayotte OR congo OR zaire OR "costa rica" OR "cote d'ivoire" OR "cote d'ivoire" OR "cote d'ivoire" OR "ivory coast" OR croatia OR cuba OR Cyprus OR "czech republic" OR czechoslovakia OR djibouti OR "french somaliland" OR dominica OR "dominican republic" OR ecuador OR egypt OR "united arab republic" OR "el salvador" OR "equatorial guinea" OR "spanish guinea" OR eritrea OR Estonia OR eswatini OR swaziland OR ethiopia OR fiji OR gabon OR "gabonese republic" OR gambia OR georgia OR Georgian OR ghana OR "gold coast" OR gibraltar OR greece OR grenada OR guam OR guatemala OR guinea OR guyana OR guiana OR haiti OR hispaniola OR honduras OR hungary OR india OR indonesia OR timor OR iran OR iraq OR "isle of man" OR jamaica OR jordan OR kazakhstan OR kazakh OR kenya OR korea OR kosovo OR kyrgyzstan OR kirghizia OR kirgizstan OR "kyrgyz republic" OR kirghiz OR laos OR "lao pdr" OR "lao people's democratic republic" OR latvia OR lebanon OR lesotho OR basutoland OR liberia OR libya OR "libyan arab OR jamahiriya" OR lithuania OR macau OR macao OR macedonia OR madagascar OR "malagasy republic" OR malawi OR nyasaland OR malaysia OR maldives OR "indian ocean" OR mali OR malta OR micronesia OR kiribati OR "marshall islands" OR nauru OR "northern mariana islands" OR palau OR tuvalu OR mauritania OR mauritius OR mexico OR moldova OR moldovian OR mongolia OR montenegro OR morocco OR ifni OR mozambique OR "portuguese east africa" OR myanmar OR burma OR namibia OR nepal OR "netherlands antilles" OR nicaragua OR niger OR nigeria OR oman OR muscat OR pakistan OR panama OR "papua new guinea" OR paraguay OR peru OR philippines OR philipines OR phillipines OR phillippines OR poland OR "polish people's republic" OR portugal OR "portuguese republic" OR "puerto rico" OR romania OR russia OR "russian federation" OR ussr OR "soviet union" OR "union of soviet socialist republics" OR rwanda OR ruanda OR samoa OR "pacific islands" OR polynesia OR "samoan islands" OR "sao tome" AND principe OR "saudi arabia" OR senegal OR serbia OR seychelles OR "sierra leone" OR slovakia OR "slovak republic" OR slovenia OR melanesia OR "solomon island" OR "solomon islands" OR "norfolk island" OR somalia OR "south africa" OR "south sudan" OR "sri lanka" OR ceylon OR "saint kitts" OR nevis OR "st kitts" OR nevis OR "saint lucia" OR "st lucia" OR "saint vincent" OR "st vincent" OR grenadines OR sudan OR suriname OR surinam OR syria OR "syrian arab republic" OR tajikistan OR tadjikistan OR tadzhikistan OR tadzhik OR tanzania OR tanganyika OR thailand OR siam OR "timor leste" OR "east timor" OR togo OR "togolese republic" OR tonga OR trinidad OR tobago OR tunisia OR turkey OR turkmenistan OR turkmen OR uganda OR ukraine OR uruguay OR Uzbekistan OR uzbek OR vanuatu OR "new hebrides" OR venezuela OR vietnam OR "vietname" OR "west bank" OR gaza OR palestine OR yemen OR yugoslavia OR zambia OR zimbabwe OR "northern rhodesia") AND pd(20100101‐20210301)	170,682
S9	AB(afghanistan OR albania OR algeria OR "american samoa" OR angola OR antigua OR barbuda OR argentina OR armenia OR armenian OR aruba OR azerbaijan OR bahrain OR bangladesh OR barbados OR belarus OR byelarus OR belorussia OR byelorussian OR belize OR "british honduras" OR benin OR dahomey OR bhutan OR bolivia OR bosnia OR herzegovina OR botswana OR bechuanaland OR brazil OR brasil OR bulgaria OR "burkina faso" OR "burkina fasso" OR "upper volta" OR burundi OR urundi OR "cabo verde" OR "cape verde" OR cambodia OR kampuchea OR "khmer republic" OR cameroon OR cameron OR cameroun OR "central african republic" OR "ubangi shari" OR chad OR chile OR china OR colombia OR comoros OR "comoro islands" OR mayotte OR congo OR zaire OR "costa rica" OR "cote d'ivoire" OR "cote d'ivoire" OR "cote d'ivoire" OR "ivory coast" OR croatia OR cuba OR Cyprus OR "czech republic" OR czechoslovakia OR djibouti OR "french somaliland" OR dominica OR "dominican republic" OR ecuador OR egypt OR "united arab republic" OR "el salvador" OR "equatorial guinea" OR "spanish guinea" OR eritrea OR Estonia OR eswatini OR swaziland OR ethiopia OR fiji OR gabon OR "gabonese republic" OR gambia OR georgia OR Georgian OR ghana OR "gold coast" OR gibraltar OR greece OR grenada OR guam OR guatemala OR guinea OR guyana OR guiana OR haiti OR hispaniola OR honduras OR hungary OR india OR indonesia OR timor OR iran OR iraq OR "isle of man" OR jamaica OR jordan OR kazakhstan OR kazakh OR kenya OR korea OR kosovo OR kyrgyzstan OR kirghizia OR kirgizstan OR "kyrgyz republic" OR kirghiz OR laos OR "lao pdr" OR "lao people's democratic republic" OR latvia OR lebanon OR lesotho OR basutoland OR liberia OR libya OR "libyan arab OR jamahiriya" OR lithuania OR macau OR macao OR macedonia OR madagascar OR "malagasy republic" OR malawi OR nyasaland OR malaysia OR maldives OR "indian ocean" OR mali OR malta OR micronesia OR kiribati OR "marshall islands" OR nauru OR "northern mariana islands" OR palau OR tuvalu OR mauritania OR mauritius OR mexico OR moldova OR moldovian OR mongolia OR montenegro OR morocco OR ifni OR mozambique OR "portuguese east africa" OR myanmar OR burma OR namibia OR nepal OR "netherlands antilles" OR nicaragua OR niger OR nigeria OR oman OR muscat OR pakistan OR panama OR "papua new guinea" OR paraguay OR peru OR philippines OR philipines OR phillipines OR phillippines OR poland OR "polish people's republic" OR portugal OR "portuguese republic" OR "puerto rico" OR romania OR russia OR "russian federation" OR ussr OR "soviet union" OR "union of soviet socialist republics" OR rwanda OR ruanda OR samoa OR "pacific islands" OR polynesia OR "samoan islands" OR "sao tome" AND principe OR "saudi arabia" OR senegal OR serbia OR seychelles OR "sierra leone" OR slovakia OR "slovak republic" OR slovenia OR melanesia OR "solomon island" OR "solomon islands" OR "norfolk island" OR somalia OR "south africa" OR "south sudan" OR "sri lanka" OR ceylon OR "saint kitts" OR nevis OR "st kitts" OR nevis OR "saint lucia" OR "st lucia" OR "saint vincent" OR "st vincent" OR grenadines OR sudan OR suriname OR surinam OR syria OR "syrian arab republic" OR tajikistan OR tadjikistan OR tadzhikistan OR tadzhik OR tanzania OR tanganyika OR thailand OR siam OR "timor leste" OR "east timor" OR togo OR "togolese republic" OR tonga OR trinidad OR tobago OR tunisia OR turkey OR turkmenistan OR turkmen OR uganda OR ukraine OR uruguay OR Uzbekistan OR uzbek OR vanuatu OR "new hebrides" OR venezuela OR vietnam OR "vietname" OR "west bank" OR gaza OR palestine OR yemen OR yugoslavia OR zambia OR zimbabwe OR "northern rhodesia") AND pd(20100101‐20210301)	169,704
S10	SU(afghanistan OR albania OR algeria OR "american samoa" OR angola OR antigua OR barbuda OR argentina OR armenia OR armenian OR aruba OR azerbaijan OR bahrain OR bangladesh OR barbados OR belarus OR byelarus OR belorussia OR byelorussian OR belize OR "british honduras" OR benin OR dahomey OR bhutan OR bolivia OR bosnia OR herzegovina OR botswana OR bechuanaland OR brazil OR brasil OR bulgaria OR "burkina faso" OR "burkina fasso" OR "upper volta" OR burundi OR urundi OR "cabo verde" OR "cape verde" OR cambodia OR kampuchea OR "khmer republic" OR cameroon OR cameron OR cameroun OR "central african republic" OR "ubangi shari" OR chad OR chile OR china OR colombia OR comoros OR "comoro islands" OR mayotte OR congo OR zaire OR "costa rica" OR "cote d'ivoire" OR "cote d'ivoire" OR "cote d'ivoire" OR "ivory coast" OR croatia OR cuba OR Cyprus OR "czech republic" OR czechoslovakia OR djibouti OR "french somaliland" OR dominica OR "dominican republic" OR ecuador OR egypt OR "united arab republic" OR "el salvador" OR "equatorial guinea" OR "spanish guinea" OR eritrea OR Estonia OR eswatini OR swaziland OR ethiopia OR fiji OR gabon OR "gabonese republic" OR gambia OR georgia OR Georgian OR ghana OR "gold coast" OR gibraltar OR greece OR grenada OR guam OR guatemala OR guinea OR guyana OR guiana OR haiti OR hispaniola OR honduras OR hungary OR india OR indonesia OR timor OR iran OR iraq OR "isle of man" OR jamaica OR jordan OR kazakhstan OR kazakh OR kenya OR korea OR kosovo OR kyrgyzstan OR kirghizia OR kirgizstan OR "kyrgyz republic" OR kirghiz OR laos OR "lao pdr" OR "lao people's democratic republic" OR latvia OR lebanon OR lesotho OR basutoland OR liberia OR libya OR "libyan arab OR jamahiriya" OR lithuania OR macau OR macao OR macedonia OR madagascar OR "malagasy republic" OR malawi OR nyasaland OR malaysia OR maldives OR "indian ocean" OR mali OR malta OR micronesia OR kiribati OR "marshall islands" OR nauru OR "northern mariana islands" OR palau OR tuvalu OR mauritania OR mauritius OR mexico OR moldova OR moldovian OR mongolia OR montenegro OR morocco OR ifni OR mozambique OR "portuguese east africa" OR myanmar OR burma OR namibia OR nepal OR "netherlands antilles" OR nicaragua OR niger OR nigeria OR oman OR muscat OR pakistan OR panama OR "papua new guinea" OR paraguay OR peru OR philippines OR philipines OR phillipines OR phillippines OR poland OR "polish people's republic" OR portugal OR "portuguese republic" OR "puerto rico" OR romania OR russia OR "russian federation" OR ussr OR "soviet union" OR "union of soviet socialist republics" OR rwanda OR ruanda OR samoa OR "pacific islands" OR polynesia OR "samoan islands" OR "sao tome" AND principe OR "saudi arabia" OR senegal OR serbia OR seychelles OR "sierra leone" OR slovakia OR "slovak republic" OR slovenia OR melanesia OR "solomon island" OR "solomon islands" OR "norfolk island" OR somalia OR "south africa" OR "south sudan" OR "sri lanka" OR ceylon OR "saint kitts" OR nevis OR "st kitts" OR nevis OR "saint lucia" OR "st lucia" OR "saint vincent" OR "st vincent" OR grenadines OR sudan OR suriname OR surinam OR syria OR "syrian arab republic" OR tajikistan OR tadjikistan OR tadzhikistan OR tadzhik OR tanzania OR tanganyika OR thailand OR siam OR "timor leste" OR "east timor" OR togo OR "togolese republic" OR tonga OR trinidad OR tobago OR tunisia OR turkey OR turkmenistan OR turkmen OR uganda OR ukraine OR uruguay OR Uzbekistan OR uzbek OR vanuatu OR "new hebrides" OR venezuela OR vietnam OR "vietname" OR "west bank" OR gaza OR palestine OR yemen OR yugoslavia OR zambia OR zimbabwe OR "northern rhodesia") AND pd(20100101‐20210301)	245,284
S11	S5 OR S6 OR S7 OR S8 OR S9 OR S10	343,316
S12	(S1 OR S2) AND S3 AND S4 AND S11	39

## APPENDIX F MEDLINE COMPLETE

Name of database: Medline Complete

Date of search: 01/03/2021

Number of results: 54

**Table:**

#	Query	Results
S17	S5 AND S6 AND S7 AND S16	**54**
S16	(S8 OR S9 OR S10 OR S11 OR S12 OR S13 OR S14 OR S15)	1,725,463
S15	**AB** (afghanistan OR albania OR algeria OR "american samoa" OR angola OR antigua OR barbuda OR argentina OR armenia OR armenian OR aruba OR azerbaijan OR bahrain OR bangladesh OR barbados OR belarus OR byelarus OR belorussia OR byelorussian OR belize OR "british honduras" OR benin OR dahomey OR bhutan OR bolivia OR bosnia OR herzegovina OR Botswana OR bechuanaland OR brazil OR brasil OR bulgaria OR "burkina faso" OR "burkina fasso" OR "upper volta" OR burundi OR urundi OR "cabo verde" OR "cape verde" OR cambodia OR kampuchea OR "khmer republic" OR cameroon OR cameron OR cameroun OR "central african republic" OR "ubangi shari" OR chad OR chile OR china OR colombia OR comoros OR "comoro islands" OR mayotte OR congo OR zaire OR "costa rica" OR "cote d'ivoire" OR "cote d'ivoire" OR "cote d'ivoire" OR "ivory coast" OR croatia OR cuba OR cyprus OR "czech republic" OR czechoslovakia OR djibouti OR "french somaliland" OR dominica OR "dominican republic" OR ecuador OR egypt OR "united arab republic" OR "el salvador" OR "equatorial guinea" OR "spanish guinea" OR eritrea OR estonia OR eswatini OR swaziland OR ethiopia OR fiji OR gabon OR "gabonese republic" OR gambia OR georgia OR Georgian OR ghana OR "gold coast" OR gibraltar OR greece OR grenada OR guam OR guatemala OR guinea OR guyana OR guiana OR haiti OR hispaniola OR honduras OR hungary OR india OR indonesia OR timor OR iran OR iraq OR "isle of man" OR jamaica OR jordan OR kazakhstan OR kazakh OR kenya OR korea OR kosovo OR kyrgyzstan OR kirghizia OR kirgizstan OR "kyrgyz republic" OR kirghiz OR laos OR "lao pdr" OR "lao people's democratic republic" OR latvia OR lebanon OR lesotho OR basutoland OR liberia OR libya OR "libyan arab jamahiriya" OR lithuania OR macau OR macao OR macedonia OR madagascar OR "malagasy republic" OR malawi OR nyasaland OR malaysia OR maldives OR "indian ocean" OR mali OR malta OR micronesia OR kiribati OR "marshall islands" OR nauru OR "northern mariana islands" OR palau OR tuvalu OR mauritania OR mauritius OR mexico OR moldova OR moldovian OR mongolia OR montenegro OR morocco OR ifni OR mozambique OR "portuguese east africa" OR myanmar OR burma OR namibia OR nepal OR "netherlands antilles" OR nicaragua OR niger OR nigeria OR oman OR muscat OR pakistan OR panama OR "papua new guinea" OR paraguay OR peru OR philippines OR philipines OR phillipines OR phillippines OR poland OR "polish people's republic" OR portugal OR "portuguese republic" OR "puerto rico" OR romania OR russia OR "russian federation" OR ussr OR "soviet union" OR "union of soviet socialist republics" OR rwanda OR ruanda OR samoa OR "pacific islands" OR polynesia OR "samoan islands" OR "sao tome" and principe OR "saudi arabia" OR senegal OR serbia OR seychelles OR "sierra leone" OR slovakia OR "slovak republic" OR slovenia OR melanesia OR "solomon island" OR "solomon islands" OR "norfolk island" OR somalia OR "south africa" OR "south sudan" OR "sri lanka" OR ceylon OR "saint kitts" OR nevis OR "st kitts" OR nevis OR "saint lucia" OR "st lucia" OR "saint vincent" OR "st vincent" OR grenadines OR sudan OR suriname OR surinam OR syria OR "syrian OR muscat OR pakistan OR panama OR "papua new guinea" OR paraguay OR peru OR philippines OR philipines OR phillipines OR phillippines OR poland OR "polish people's republic" OR portugal OR "portuguese republic" OR "puerto rico" OR romania OR russia OR "russian federation" OR ussr OR "soviet union" OR "union of soviet socialist republics" OR rwanda OR ruanda OR samoa OR "pacific islands" OR polynesia OR "samoan islands" OR "sao tome" and principe OR "saudi arabia" OR senegal OR serbia OR seychelles OR "sierra leone" OR slovakia OR "slovak republic" OR slovenia OR melanesia OR "solomon island" OR "solomon islands" OR "norfolk island" OR somalia OR "south africa" OR "south sudan" OR "sri lanka" OR ceylon OR "saint kitts" OR nevis OR "st kitts" OR nevis OR "saint lucia" OR "st lucia" OR "saint vincent" OR "st vincent" OR grenadines OR sudan OR suriname OR surinam OR syria OR "syrian arab republic" OR tajikistan OR tadjikistan OR tadzhikistan OR tadzhik OR tanzania OR Tanganyika OR thailand OR siam OR "timor leste" OR "east timor" OR togo OR "togolese republic" OR tonga OR trinidad OR tobago OR tunisia OR turkey OR turkmenistan OR turkmen OR uganda OR ukraine OR uruguay OR uzbekistan OR uzbek OR vanuatu OR "new hebrides" OR venezuela OR vietnam OR "viet name" OR "west bank" OR gaza OR palestine OR yemen OR yugoslavia OR zambia OR zimbabwe OR "northern rhodesia") OR **TI** (afghanistan OR albania OR algeria OR "american samoa" OR angola OR antigua OR barbuda OR argentina OR armenia OR armenian OR aruba OR azerbaijan OR bahrain OR bangladesh OR barbados OR belarus OR byelarus OR belorussia OR byelorussian OR belize OR "british honduras" OR benin OR dahomey OR bhutan OR bolivia OR bosnia OR herzegovina OR Botswana OR bechuanaland OR brazil OR brasil OR bulgaria OR "burkina faso" OR "burkina fasso" OR "upper volta" OR burundi OR urundi OR "cabo verde" OR "cape verde" OR cambodia OR kampuchea OR "khmer republic" OR cameroon OR cameron OR cameroun OR "central african republic" OR "ubangi shari" OR chad OR chile OR china OR colombia OR comoros OR "comoro islands" OR mayotte OR congo OR zaire OR "costa rica" OR "cote d'ivoire" OR "cote d'ivoire" OR "cote d'ivoire" OR "ivory coast" OR croatia OR cuba OR cyprus OR "czech republic" OR czechoslovakia OR djibouti OR "french somaliland" OR dominica OR "dominican republic" OR ecuador OR egypt OR "united arab republic" OR "el salvador" OR "equatorial guinea" OR "spanish guinea" OR eritrea OR estonia OR eswatini OR swaziland OR ethiopia OR fiji OR gabon OR "gabonese republic" OR gambia OR georgia OR Georgian OR ghana OR "gold coast" OR gibraltar OR greece OR grenada OR guam OR guatemala OR guinea OR guyana OR guiana OR haiti OR hispaniola OR honduras OR hungary OR india OR indonesia OR timor OR iran OR iraq OR "isle of man" OR jamaica OR jordan OR kazakhstan OR kazakh OR kenya OR korea OR kosovo OR kyrgyzstan OR kirghizia OR kirgizstan OR "kyrgyz republic" OR kirghiz OR laos OR "lao pdr" OR "lao people's democratic republic" OR latvia OR lebanon OR lesotho OR basutoland OR liberia OR libya OR "libyan arab jamahiriya" OR lithuania OR macau OR macao OR macedonia OR madagascar OR "malagasy republic" OR malawi OR nyasaland OR malaysia OR maldives OR "indian ocean" OR mali OR malta OR micronesia OR kiribati OR "marshall islands" OR nauru OR "northern mariana islands" OR palau OR tuvalu OR mauritania OR mauritius OR mexico OR moldova OR moldovian OR mongolia OR montenegro OR morocco OR ifni OR mozambique OR "portuguese east africa" OR myanmar OR burma OR namibia OR nepal OR "netherlands antilles" OR nicaragua OR niger OR nigeria OR oman OR muscat OR pakistan OR panama OR "papua new guinea" OR paraguay OR peru OR philippines OR philipines OR phillipines OR phillippines OR poland OR "polish people's republic" OR portugal OR "portuguese republic" OR "puerto rico" OR romania OR russia OR "russian federation" OR ussr OR "soviet union" OR "union of soviet socialist republics" OR rwanda OR ruanda OR samoa OR "pacific islands" OR polynesia OR "samoan islands" OR "sao tome" and principe OR "saudi arabia" OR senegal OR serbia OR seychelles OR "sierra leone" OR slovakia OR "slovak republic" OR slovenia OR melanesia OR "solomon island" OR "solomon islands" OR "norfolk island" OR somalia OR "south africa" OR "south sudan" OR "sri lanka" OR ceylon OR "saint kitts" OR nevis OR "st kitts" OR nevis OR "saint lucia" OR "st lucia" OR "saint vincent" OR "st vincent" OR grenadines OR sudan OR suriname OR surinam OR syria OR "syrian OR muscat OR pakistan OR panama OR "papua new guinea" OR paraguay OR peru OR philippines OR philipines OR phillipines OR phillippines OR poland OR "polish people's republic" OR portugal OR "portuguese republic" OR "puerto rico" OR romania OR russia OR "russian federation" OR ussr OR "soviet union" OR "union of soviet socialist republics" OR rwanda OR ruanda OR samoa OR "pacific islands" OR polynesia OR "samoan islands" OR "sao tome" and principe OR "saudi arabia" OR senegal OR serbia OR seychelles OR "sierra leone" OR slovakia OR "slovak republic" OR slovenia OR melanesia OR "solomon island" OR "solomon islands" OR "norfolk island" OR somalia OR "south africa" OR "south sudan" OR "sri lanka" OR ceylon OR "saint kitts" OR nevis OR "st kitts" OR nevis OR "saint lucia" OR "st lucia" OR "saint vincent" OR "st vincent" OR grenadines OR sudan OR suriname OR surinam OR syria OR "syrian arab republic" OR tajikistan OR tadjikistan OR tadzhikistan OR tadzhik OR tanzania OR Tanganyika OR thailand OR siam OR "timor leste" OR "east timor" OR togo OR "togolese republic" OR tonga OR trinidad OR tobago OR tunisia OR turkey OR turkmenistan OR turkmen OR uganda OR ukraine OR uruguay OR uzbekistan OR uzbek OR vanuatu OR "new hebrides" OR venezuela OR vietnam OR "viet name" OR "west bank" OR gaza OR palestine OR yemen OR yugoslavia OR zambia OR zimbabwe OR "northern rhodesia") OR **SU** (afghanistan OR albania OR algeria OR "american samoa" OR angola OR antigua OR barbuda OR argentina OR armenia OR armenian OR aruba OR azerbaijan OR bahrain OR bangladesh OR barbados OR belarus OR byelarus OR belorussia OR byelorussian OR belize OR "british honduras" OR benin OR dahomey OR bhutan OR bolivia OR bosnia OR herzegovina OR Botswana OR bechuanaland OR brazil OR brasil OR bulgaria OR "burkina faso" OR "burkina fasso" OR "upper volta" OR burundi OR urundi OR "cabo verde" OR "cape verde" OR cambodia OR kampuchea OR "khmer republic" OR cameroon OR cameron OR cameroun OR "central african republic" OR "ubangi shari" OR chad OR chile OR china OR colombia OR comoros OR "comoro islands" OR mayotte OR congo OR zaire OR "costa rica" OR "cote d'ivoire" OR "cote d'ivoire" OR "cote d'ivoire" OR "ivory coast" OR croatia OR cuba OR cyprus OR "czech republic" OR czechoslovakia OR djibouti OR "french somaliland" OR dominica OR "dominican republic" OR ecuador OR egypt OR "united arab republic" OR "el salvador" OR "equatorial guinea" OR "spanish guinea" OR eritrea OR estonia OR eswatini OR swaziland OR ethiopia OR fiji OR gabon OR "gabonese republic" OR gambia OR georgia OR Georgian OR ghana OR "gold coast" OR gibraltar OR greece OR grenada OR guam OR guatemala OR guinea OR guyana OR guiana OR haiti OR hispaniola OR honduras OR hungary OR india OR indonesia OR timor OR iran OR iraq OR "isle of man" OR jamaica OR jordan OR kazakhstan OR kazakh OR kenya OR korea OR kosovo OR kyrgyzstan OR kirghizia OR kirgizstan OR "kyrgyz republic" OR kirghiz OR laos OR "lao pdr" OR "lao people's democratic republic" OR latvia OR lebanon OR lesotho OR basutoland OR liberia OR libya OR "libyan arab jamahiriya" OR lithuania OR macau OR macao OR macedonia OR madagascar OR "malagasy republic" OR malawi OR nyasaland OR malaysia OR maldives OR "indian ocean" OR mali OR malta OR micronesia OR kiribati OR "marshall islands" OR nauru OR "northern mariana islands" OR palau OR tuvalu OR mauritania OR mauritius OR mexico OR moldova OR moldovian OR mongolia OR montenegro OR morocco OR ifni OR mozambique OR "portuguese east africa" OR myanmar OR burma OR namibia OR nepal OR "netherlands antilles" OR nicaragua OR niger OR nigeria OR oman OR muscat OR pakistan OR panama OR "papua new guinea" OR paraguay OR peru OR philippines OR philipines OR phillipines OR phillippines OR poland OR "polish people's republic" OR portugal OR "portuguese republic" OR "puerto rico" OR romania OR russia OR "russian federation" OR ussr OR "soviet union" OR "union of soviet socialist republics" OR rwanda OR ruanda OR samoa OR "pacific islands" OR polynesia OR "samoan islands" OR "sao tome" and principe OR "saudi arabia" OR senegal OR serbia OR seychelles OR "sierra leone" OR slovakia OR "slovak republic" OR slovenia OR melanesia OR "solomon island" OR "solomon islands" OR "norfolk island" OR somalia OR "south africa" OR "south sudan" OR "sri lanka" OR ceylon OR "saint kitts" OR nevis OR "st kitts" OR nevis OR "saint lucia" OR "st lucia" OR "saint vincent" OR "st vincent" OR grenadines OR sudan OR suriname OR surinam OR syria OR "syrian OR muscat OR pakistan OR panama OR "papua new guinea" OR paraguay OR peru OR philippines OR philipines OR phillipines OR phillippines OR poland OR "polish people's republic" OR portugal OR "portuguese republic" OR "puerto rico" OR romania OR russia OR "russian federation" OR ussr OR "soviet union" OR "union of soviet socialist republics" OR rwanda OR ruanda OR samoa OR "pacific islands" OR polynesia OR "samoan islands" OR "sao tome" and principe OR "saudi arabia" OR senegal OR serbia OR seychelles OR "sierra leone" OR slovakia OR "slovak republic" OR slovenia OR melanesia OR "solomon island" OR "solomon islands" OR "norfolk island" OR somalia OR "south africa" OR "south sudan" OR "sri lanka" OR ceylon OR "saint kitts" OR nevis OR "st kitts" OR nevis OR "saint lucia" OR "st lucia" OR "saint vincent" OR "st vincent" OR grenadines OR sudan OR suriname OR surinam OR syria OR "syrian arab republic" OR tajikistan OR tadjikistan OR tadzhikistan OR tadzhik OR tanzania OR Tanganyika OR thailand OR siam OR "timor leste" OR "east timor" OR togo OR "togolese republic" OR tonga OR trinidad OR tobago OR tunisia OR turkey OR turkmenistan OR turkmen OR uganda OR ukraine OR uruguay OR uzbekistan OR uzbek OR vanuatu OR "new hebrides" OR venezuela OR vietnam OR "viet name" OR "west bank" OR gaza OR palestine OR yemen OR yugoslavia OR zambia OR zimbabwe OR "northern rhodesia")	1,757,015
S14	**TI** (("transitional country" OR "transitional countries")) OR AB (("transitional country" OR "transitional countries")) OR SU (("transitional country" OR "transitional countries"))	167
S13	**TI** ((emerging N0 (economy or economies or nation*))) OR **AB** ((emerging N0 (economy or economies or nation*))) OR **SU** ((emerging N0 (economy or economies or nation*)))	707
S12	**TI** (((low* N1 (gdp OR gnp OR "gross domestic" OR "gross national"))) OR **AB** (((low* N1 (gdp OR gnp OR "gross domestic" OR "gross national"))) OR **SU** (((low* N1 (gdp OR gnp OR "gross domestic" OR "gross national")))	398
S11	TI (low N3 middle N3 countr*) OR AB (low N3 middle N3 countr*) OR SU (low N3 middle N3 countr*)	20,150
S10	**TI** (((developing OR (less* N1 developed) OR "under developed" OR "middle income" or (low* N1 income)) N1 (economy or economies))) OR **AB** (((developing OR (less* N1 developed) OR "under developed" OR "middle income" or (low* N1 income)) N1 (economy or economies))) OR **SU** (((developing OR (less* N1 developed) OR "under developed" OR "middle income" or (low* N1 income)) N1 (economy or economies)))	730
S9	**TI** (((developing OR (less* N1 developed) OR "under developed" OR underdeveloped OR "under served" OR underserved OR deprived OR poor* OR "middle income" or (low* N1 income)) N1 (countr* or nation* or population* or world))) OR **AB** (((developing OR (less* N1 developed) OR "under developed" OR underdeveloped OR "under served" OR underserved OR deprived OR poor* OR "middle income" or (low* N1 income)) N1 (countr* or nation* or population* or world))) OR **SU** (((developing OR (less* N1 developed) OR "under developed" OR underdeveloped OR "under served" OR underserved OR deprived OR poor* OR "middle income" or (low* N1 income)) N1 (countr* or nation* or population* or world)))	171,336
S8	**TI** (Africa OR Asia OR Caribbean OR "West Indies" OR "South America" OR "Latin America" OR "Central America" OR "Middle East" OR "global south") OR **AB** (Africa OR Asia OR Caribbean OR "West Indies" OR "South America" OR "Latin America" OR "Central America" OR "Middle East" OR "global south") OR **SU** (Africa OR Asia OR Caribbean OR "West Indies" OR "South America" OR "Latin America" OR "Central America" OR "Middle East" OR "global south")	303,651
S7	**AB** ((women OR "women's right" OR "young women" OR woman* OR adolescent* OR girl* OR schoolgirl* OR "school girl*" OR female* OR man OR men OR "young men" OR boy* OR schoolboy* OR "school boy*" OR teen* OR youth* OR young* OR mother* OR father* OR parent* OR caregiver) OR (femini* OR masculi* OR female* OR male* OR gender* OR "social equality" OR "social equity" OR empower* OR "equal opportunit*" OR egalitarian*)) OR **SU** ((women OR "women's right" OR "young women" OR woman* OR adolescent* OR girl* OR schoolgirl* OR "school girl*" OR female* OR man OR men OR "young men" OR boy* OR schoolboy* OR "school boy*" OR teen* OR youth* OR young* OR mother* OR father* OR parent* OR caregiver) OR (femini* OR masculi* OR female* OR male* OR gender* OR "social equality" OR "social equity" OR empower* OR "equal opportunit*" OR egalitarian*)) OR **TI** ((women OR "women's right" OR "young women" OR woman* OR adolescent* OR girl* OR schoolgirl* OR "school girl*" OR female* OR man OR men OR "young men" OR boy* OR schoolboy* OR "school boy*" OR teen* OR youth* OR young* OR mother* OR father* OR parent* OR caregiver) OR (femini* OR masculi* OR female* OR male* OR gender* OR "social equality" OR "social equity" OR empower* OR "equal opportunit*" OR egalitarian*))	13,196,523
S6	(**TI** (("literature search" OR "database search" OR "bibliographic* search" OR comprehensive search" OR “extensive search" OR "exhaustive search" OR "purposive search" OR "representative search" OR "systemat* search") OR **AB** (("literature search" OR "database search" OR "bibliographic* search" OR comprehensive search" OR “extensive search" OR "exhaustive search" OR "purposive search" OR "representative search" OR "systemat* search") OR **SU** (("literature search" OR "database search" OR "bibliographic* search" OR comprehensive search" OR “extensive search" OR "exhaustive search" OR "purposive search" OR "representative search" OR "systemat* search")) OR (**TI** ((review N3 (effectiveness OR effects OR systemat* OR synth* OR integrat* OR map* OR methodologic* OR quantitative OR evidence OR literature)) OR **AB** ((review N3 (effectiveness OR effects OR systemat* OR synth* OR integrat* OR map* OR methodologic* OR quantitative OR evidence OR literature)) OR **SU** ((review N3 (effectiveness OR effects OR systemat* OR synth* OR integrat* OR map* OR methodologic* OR quantitative OR evidence OR literature))) OR (**TI** ((("meta regression" OR "meta synth*" OR "meta‐synth*" OR "meta analy*" OR "metaanaly*" OR "meta‐analy*" OR "metanaly*" OR "metaregression" OR "meta‐regression" OR "methodologic* overview" OR "pool* analys*" OR "pool* data" OR "quantitative* overview" OR "research integration"))) OR **AB** ((("meta regression" OR "meta synth*" OR "meta‐synth*" OR "meta analy*" OR "metaanaly*" OR "meta‐analy*" OR "metanaly*" OR "metaregression" OR "meta‐regression" OR "methodologic* overview" OR "pool* analys*" OR "pool* data" OR "quantitative* overview" OR "research integration"))) OR **SU** ((("meta regression" OR "meta synth*" OR "meta‐synth*" OR "meta analy*" OR "metaanaly*" OR "meta‐analy*" OR "metanaly*" OR "metaregression" OR "meta‐regression" OR "methodologic* overview" OR "pool* analys*" OR "pool* data" OR "quantitative* overview" OR "research integration")))) OR (**TI** (((systematic* OR synthes*) N3 (research OR evaluation* OR finding* OR thematic* OR report OR descriptive OR explanatory OR narrative OR meta* OR review* OR data OR literature OR studies OR evidence OR map OR quantitative OR study OR studies OR paper OR impact OR impacts OR effect* OR compar*))) OR **AB** (((systematic* OR synthes*) N3 (research OR evaluation* OR finding* OR thematic* OR report OR descriptive OR explanatory OR narrative OR meta* OR review* OR data OR literature OR studies OR evidence OR map OR quantitative OR study OR studies OR paper OR impact OR impacts OR effect* OR compar*))) OR **SU** (((systematic* OR synthes*) N3 (research OR evaluation* OR finding* OR thematic* OR report OR descriptive OR explanatory OR narrative OR meta* OR review* OR data OR literature OR studies OR evidence OR map OR quantitative OR study OR studies OR paper OR impact OR impacts OR effect* OR compar*))))	427,674
S5	S1 OR S2 OR S3 OR S4	237,830
S4	**SU** ((((family OR birth OR income) N1(allowance OR maintenance OR grant OR payment)) OR (leave N1(parental OR paternity OR maternity OR sick)) OR (welfare N1(infant OR maternal)) OR (support N1(disability OR nutritional OR compensatory)) OR (voucher N1(food OR school OR education)) OR (assistance N1(humanitarian)) OR (service* N2("ancillary school" OR homemaker OR "school health" OR "child care*" OR community OR "home care" OR "adult protective")) OR(aid N4 ("to families with dependent children" OR "to the totally disabled" OR "to visually impaired")) OR (community* N1(care OR intervention OR integration OR project)) OR (care N2(day OR "for children" OR "for elderly" OR "foster home" OR "centre‐based child" OR "center‐based child")) OR (education N1(parenting OR vocational)) OR (tax N1(exemption OR break OR credit*)) OR ("after school club" OR "after school program*" OR family support))) OR **AB** ((((family OR birth OR income) N1(allowance OR maintenance OR grant OR payment)) OR (leave N1(parental OR paternity OR maternity OR sick)) OR (welfare N1(infant OR maternal)) OR (support N1(disability OR nutritional OR compensatory)) OR (voucher N1(food OR school OR education)) OR (assistance N1(humanitarian)) OR (service* N2("ancillary school" OR homemaker OR "school health" OR "child care*" OR community OR "home care" OR "adult protective")) OR(aid N4 ("to families with dependent children" OR "to the totally disabled" OR "to visually impaired")) OR (community* N1(care OR intervention OR integration OR project)) OR (care N2(day OR "for children" OR "for elderly" OR "foster home" OR "centre‐based child" OR "center‐based child")) OR (education N1(parenting OR vocational)) OR (tax N1(exemption OR break OR credit*)) OR ("after school club" OR "after school program*" OR family support))) OR **TI** ((((family OR birth OR income) N1(allowance OR maintenance OR grant OR payment)) OR (leave N1(parental OR paternity OR maternity OR sick)) OR (welfare N1(infant OR maternal)) OR (support N1(disability OR nutritional OR compensatory)) OR (voucher N1(food OR school OR education)) OR (assistance N1(humanitarian)) OR (service* N2("ancillary school" OR homemaker OR "school health" OR "child care*" OR community OR "home care" OR "adult protective")) OR(aid N4 ("to families with dependent children" OR "to the totally disabled" OR "to visually impaired")) OR (community* N1(care OR intervention OR integration OR project)) OR (care N2(day OR "for children" OR "for elderly" OR "foster home" OR "centre‐based child" OR "center‐based child")) OR (education N1(parenting OR vocational)) OR (tax N1(exemption OR break OR credit*)) OR ("after school club" OR "after school program*" OR family support)))	100,664
S3	**SU** ((("economic asset" OR "fee waiver*" OR "school feeding" OR "disability grant*" OR "food stamp" OR "sick pay" OR medicaid OR medicare OR "home health nursing" OR "nurse family partnership") OR (intervention* N3("intimate partner violence" OR "domestic violence" OR "domestic abuse" OR ipv OR "family violence" OR "partner violence" OR "spous* abuse" OR "battered women" OR abusive)))) OR **AB** ((("economic asset" OR "fee waiver*" OR "school feeding" OR "disability grant*" OR "food stamp" OR "sick pay" OR medicaid OR medicare OR "home health nursing" OR "nurse family partnership") OR (intervention* N3("intimate partner violence" OR "domestic violence" OR "domestic abuse" OR ipv OR "family violence" OR "partner violence" OR "spous* abuse" OR "battered women" OR abusive)))) OR **TI** ((("economic asset" OR "fee waiver*" OR "school feeding" OR "disability grant*" OR "food stamp" OR "sick pay" OR medicaid OR medicare OR "home health nursing" OR "nurse family partnership") OR (intervention* N3("intimate partner violence" OR "domestic violence" OR "domestic abuse" OR ipv OR "family violence" OR "partner violence" OR "spous* abuse" OR "battered women" OR abusive))))	40,613
S2	**SU** (((labo#r market N1(policy OR policies OR program* OR reform * OR standard OR supply)) OR (employment N1(scheme OR support OR service)) OR (training N1 (vocational OR job OR support OR employee)) OR (subsid* N1(wage OR hiring OR health)) OR ("activation policies" OR "skills development" OR "skill development" or "entrepreneurship promotion" or "public works") OR (insurance N2 (health OR household OR community‐based OR national OR unemployment OR micro‐health OR "micro health")) OR (child NEAR/2(support OR welfare OR grant OR care OR "care cash")) OR (assistance N2 (medical OR employee OR nutrition OR food OR public OR social OR "old age")) OR (benefit* N1(paternity OR maternity OR system OR retirement OR unemployment OR fringe OR employee OR health OR child OR death OR system OR insurance)) OR ((wage OR housing) N1(subsidy OR subsidies OR public OR elderly)))) OR **AB** (((labo#r market N1(policy OR policies OR program* OR reform * OR standard OR supply)) OR (employment N1(scheme OR support OR service)) OR (training N1 (vocational OR job OR support OR employee)) OR (subsid* N1(wage OR hiring OR health)) OR ("activation policies" OR "skills development" OR "skill development" or "entrepreneurship promotion" or "public works") OR (insurance N2 (health OR household OR community‐based OR national OR unemployment OR micro‐health OR "micro health")) OR (child NEAR/2(support OR welfare OR grant OR care OR "care cash")) OR (assistance N2 (medical OR employee OR nutrition OR food OR public OR social OR "old age")) OR (benefit* N1(paternity OR maternity OR system OR retirement OR unemployment OR fringe OR employee OR health OR child OR death OR system OR insurance)) OR ((wage OR housing) N1(subsidy OR subsidies OR public OR elderly)))) OR **TI** (((labo#r market N1(policy OR policies OR program* OR reform * OR standard OR supply)) OR (employment N1(scheme OR support OR service)) OR (training N1 (vocational OR job OR support OR employee)) OR (subsid* N1(wage OR hiring OR health)) OR ("activation policies" OR "skills development" OR "skill development" or "entrepreneurship promotion" or "public works") OR (insurance N2 (health OR household OR community‐based OR national OR unemployment OR micro‐health OR "micro health")) OR (child NEAR/2(support OR welfare OR grant OR care OR "care cash")) OR (assistance N2 (medical OR employee OR nutrition OR food OR public OR social OR "old age")) OR (benefit* N1(paternity OR maternity OR system OR retirement OR unemployment OR fringe OR employee OR health OR child OR death OR system OR insurance)) OR ((wage OR housing) N1(subsidy OR subsidies OR public OR elderly))))	82,525
S1	**SU** ((((social N1(care* OR protection OR insurance OR policy OR policies OR inclusion OR security or assistance or service* OR welfare OR grant OR pension* OR "safety net"))) OR (program* N2 ("cash plus" OR lunch OR breakfast OR "school health" OR "food security" OR "poverty alleviation" OR "poverty reduction" OR livelihood* OR "employee assistance" OR employment OR empowerment OR entrepreneurship OR enterpreneurship OR employment OR "employee assistance" OR "youth employment" OR integrated OR community‐based)) OR (transfer N1(resource OR in‐kind OR conditional OR unconditional OR financial OR monetary)) OR (pension N1(retirement OR public OR "old age")) OR (financial N1(incentive OR protection)))) OR **AB** ((((social N1(care* OR protection OR insurance OR policy OR policies OR inclusion OR security or assistance or service* OR welfare OR grant OR pension* OR "safety net"))) OR (program* N2 ("cash plus" OR lunch OR breakfast OR "school health" OR "food security" OR "poverty alleviation" OR "poverty reduction" OR livelihood* OR "employee assistance" OR employment OR empowerment OR entrepreneurship OR enterpreneurship OR employment OR "employee assistance" OR "youth employment" OR integrated OR community‐based)) OR (transfer N1(resource OR in‐kind OR conditional OR unconditional OR financial OR monetary)) OR (pension N1(retirement OR public OR "old age")) OR (financial N1(incentive OR protection)))) OR **TI** ((((social N1(care* OR protection OR insurance OR policy OR policies OR inclusion OR security or assistance or service* OR welfare OR grant OR pension* OR "safety net"))) OR (program* N2 ("cash plus" OR lunch OR breakfast OR "school health" OR "food security" OR "poverty alleviation" OR "poverty reduction" OR livelihood* OR "employee assistance" OR employment OR empowerment OR entrepreneurship OR enterpreneurship OR employment OR "employee assistance" OR "youth employment" OR integrated OR community‐based)) OR (transfer N1(resource OR in‐kind OR conditional OR unconditional OR financial OR monetary)) OR (pension N1(retirement OR public OR "old age")) OR (financial N1(incentive OR protection))))	36,348

## APPENDIX G PsycINFO

Name of database: PsycINFO

Date of search: 26/02/2021

Number of results: 391

**Table:**

#	Query	Limiters/Expanders	Last Run Via	Results
S5	S1 AND S2 AND S3 AND S4	Expanders ‐ Apply equivalent subjects Search modes ‐ Boolean/Phrase	Interface ‐ EBSCOhost Research Databases Search Screen ‐ Advanced Search Database ‐ APA PsycArticles;APA PsycInfo	398
S4	(TI((social N1 (care* OR protection OR insurance OR policy OR policies OR inclusion OR security OR assistance OR service* OR welfare OR grant OR pension* OR "safety net")) OR ((program* N2("cash plus" OR lunch OR breakfast OR "school health" OR "food security" OR "poverty alleviation" or "poverty reduction" OR livelihood* OR "employee assistance" OR "employment" OR empowerment OR entrepreneurship OR enterpreneurship OR employment OR "employee assistance" OR "youth employment" OR integrated OR “community based”)) OR (transfer N1(resource OR in‐kind OR conditional OR unconditional OR financial OR monetary")) OR (pension N1(retirement OR public OR "old age")) OR (financial N1(incentive or protection))) OR (AB((social N1 (care* OR protection OR insurance OR policy OR policies OR inclusion OR security OR assistance OR service* OR welfare OR grant OR pension* OR "safety net")) OR ((program* N2("cash plus" OR lunch OR breakfast OR "school health" OR "food security" OR "poverty alleviation" OR "poverty reduction" OR livelihood* OR "employee assistance" OR "employment" OR empowerment OR entrepreneurship OR enterpreneurship OR employment OR "employee assistance" OR "youth employment" OR integrated OR “community based”)) OR (transfer N1(resource OR in‐kind OR conditional OR unconditional OR financial OR monetary")) OR (pension N1(retirement OR public OR "old age")) OR (financial N1(incentive or protection))) OR (SU((social N1 (care* OR protection OR insurance OR policy OR policies OR inclusion OR security OR assistance OR service* OR welfare OR grant OR pension* OR "safety net")) OR ((program* N2("cash plus" OR lunch OR breakfast OR "school health" OR "food security" OR "poverty alleviation" or "poverty reduction" OR livelihood* OR "employee assistance" OR "employment" OR empowerment OR entrepreneurship OR enterpreneurship OR employment OR "employee assistance" OR "youth employment" OR integrated OR “community based”)) OR (transfer N1(resource OR in‐kind OR conditional OR unconditional OR financial OR monetary")) OR (pension N1(retirement OR public OR "old age")) OR (financial N1(incentive OR protection))) OR (TI(labo$r market N1(policy OR policies OR program* OR reform* OR standard OR supply)) OR (employment N1(scheme OR support OR service)) OR (training N1 (vocational OR job OR support OR employee)) OR (subsid* N1(wage OR hiring OR health)) OR ("activation policies" OR "skills development" OR "skill development" OR "entrepreneurship promotion" OR "public works") OR (insurance N2 (health OR household OR “community based” OR national OR unemployment OR micro‐health OR "micro Health")) OR(child N2 (support OR welfare OR grant OR care OR "care cash")) OR (assistance N2(medical OR employee OR nutrition OR food OR public OR social OR "old age")) OR (benefit* N1(paternity OR maternity OR system OR retirement OR unemployment OR fringe OR employee OR health OR "child OR death OR system OR insurance)) OR ((wage OR housing) N1 (subsidy OR subsidies OR public OR elderly))) OR (AB(labo$r market N1(policy OR policies OR program* OR reform* OR standard OR supply)) OR (employment N1(scheme OR support OR service)) OR (training N1 (vocational OR job OR support OR employee)) OR (subsid* N1(wage OR hiring OR health)) OR ("activation policies" OR "skills development" OR "skill development" OR "entrepreneurship promotion" OR "public works") OR (insurance N2 (health OR household OR “community based” OR national OR unemployment OR micro‐health OR "micro health")) OR(child N2 (support OR welfare OR grant OR care OR "care cash")) OR (assistance N2(medical OR employee OR nutrition OR food OR public OR social OR "old age")) OR (benefit* N1(paternity OR maternity OR system OR retirement OR unemployment OR fringe OR employee OR health OR "child OR death OR system OR insurance)) OR ((wage OR housing) N1 (subsidy OR subsidies OR public OR elderly))) OR (SU(labo$r market N1(policy OR policies OR program* OR reform* OR standard OR supply)) OR (employment N1(scheme OR support OR service)) OR (training N1(vocational OR job OR support OR employee)) OR (subsid* N1(wage OR hiring OR health)) OR ("activation policies" OR "skills development" OR "skill development" OR "entrepreneurship promotion" or "public works") OR (insurance N2(health OR household OR “community based” OR national OR unemployment OR micro‐health OR "micro health")) OR(child N2(support OR welfare OR grant OR care OR "care cash")) OR (assistance N2(medical OR employee OR nutrition OR food OR public OR social OR "old age")) OR (benefit* N1(paternity OR maternity OR system OR retirement OR unemployment OR fringe OR employee OR health OR "child OR death OR system OR insurance)) OR ((wage OR housing) N1(subsidy OR subsidies OR public OR elderly))) OR (TI(income N2(support OR maintenance)) OR (family N2(support OR allowance OR grant)) OR (leave N1(parental OR paternity OR maternity OR sick OR disability)) OR (welfare N1(infant OR child OR maternal)) OR (birth N1(grant OR payment)) OR (income N1(support OR maintenance OR minimum) OR (voucher N1(food OR school) OR (assistance N1(nutritional OR medical OR “old age” OR humanitarian) OR (service* N2("ancillary school" OR homemaker OR "school health" OR "child care*" OR community OR "home care" OR "adult protective")) OR (support N1(family OR disability OR "nutritional OR compensatory OR child)) OR (community* N1(care OR intervention OR integration OR project)) OR (aid N3("to families with dependent children" OR "to the totally disabled" OR "to visually impaired")) OR (care N2(day OR "for children" OR "for elderly" OR "foster home" OR "centre‐based child" OR "center‐based child")) OR (education N1(parenting OR vocational OR vouchers)) OR (tax N1(exemption OR break OR credit*) OR (after school N1(club OR program*))) OR (AB(income N2(support OR maintenance)) OR (family N2(support OR allowance OR grant)) OR (leave N1(parental OR paternity OR maternity OR sick OR disability)) OR (welfare N1(infant OR child OR maternal)) OR (birth N1(grant OR payment)) OR (income N1(support OR maintenance OR minimum) OR (voucher N1(food OR school) OR (assistance N1(nutritional OR medical OR “old age” OR humanitarian) OR (service* N2("ancillary school" OR homemaker OR "school health" OR "child care*" OR community OR "home care" OR "adult protective")) OR (support N1(family OR disability OR "nutritional OR compensatory OR child)) OR (community* N1(care OR intervention OR integration OR project)) OR (aid N3("to families with dependent children" OR "to the totally disabled" OR "to visually impaired")) OR (care N2(day OR "for children" OR "for elderly" OR "foster home" OR "centre‐based child" OR "center‐based child")) OR (education N1(parenting OR vocational OR vouchers)) OR (tax N1(exemption OR break OR credit*) OR (after school N1(club OR program*))) OR (SU(income N2(support OR maintenance)) OR (family N2(support OR allowance OR grant)) OR (leave N1(parental OR paternity OR maternity OR sick OR disability)) OR (welfare N1(infant OR child OR maternal)) OR (birth N1(grant OR payment)) OR (income N1(support OR maintenance OR minimum) OR (voucher N1(food OR school) OR (assistance N1(nutritional OR medical OR “old age” OR humanitarian) OR (service* N2("ancillary school" OR homemaker OR "school health" OR "child care*" OR community OR "home care" OR "adult protective")) OR (support N1(family OR disability OR "nutritional OR compensatory OR child)) OR (community* N1(care OR intervention OR integration OR project)) OR (aid N3("to families with dependent children" OR "to the totally disabled" OR "to visually impaired")) OR (care N2(day OR "for children" OR "for elderly" OR "foster home" OR "centre‐based child" OR "center‐based child")) OR (education N1(parenting OR vocational OR vouchers)) OR (tax N1(exemption OR break OR credit*) OR (after school N1(club OR program*))) OR (TI("economic asset" OR "fee waiver*" OR "school feeding" OR "disability grant*" OR "food stamp" OR "sick pay" or "medicaid" OR medicare OR "home health nursing" OR "nurse family partnership") OR (intervention* NEAR/3(“intimate partner violence” OR “domestic violence” OR “domestic abuse” OR ipv OR “family violence” OR “partner violence” OR “spous* abuse” OR “battered women” OR abusive)) OR (AB("economic asset" OR "fee waiver*" OR "school feeding" OR "disability grant*" OR "food stamp" OR "sick pay" or "medicaid" OR medicare OR "home health nursing" OR "nurse family partnership") OR (intervention* NEAR/3(“intimate partner violence” OR “domestic violence” OR “domestic abuse” OR ipv OR “family violence” OR “partner violence” OR “spous* abuse” OR “battered women” OR abusive)) OR (SU("economic asset" OR "fee waiver*" OR "school feeding" OR "disability grant*" OR "food stamp" OR "sick pay" or "medicaid" OR medicare OR "home health nursing" OR "nurse family partnership") OR (intervention* NEAR/3(“intimate partner violence” OR “domestic violence” OR “domestic abuse” OR ipv OR “family violence” OR “partner violence” OR “spous* abuse” OR “battered women” OR abusive))	Limiters ‐ Year of Publication: 2010‐2021 Expanders ‐ Apply equivalent subjects Search modes ‐ Boolean/Phrase	Interface ‐ EBSCOhost Research Databases Search Screen ‐ Advanced Search Database ‐ APA PsycArticles;APA PsycInfo	136,948
S3	(TI("literature search" OR "database search" OR "bibliographic* search" OR comprehensive search" OR “extensive search" OR "exhaustive search" OR "purposive search" OR "representative search" OR "systemat* search") OR AB("literature search" OR "database search" OR "bibliographic* search" OR comprehensive search" OR extensive search" OR "exhaustive search" OR "purposive search" OR "representative search" OR "systemat* search") OR SU("literature search" OR "database search" OR "bibliographic* search" OR comprehensive search" OR extensive search" OR "exhaustive search" OR "purposive search" OR "representative search" OR "systemat* search")) OR (TI(review N3 (effectiveness OR effects OR systemat* OR synth* OR integrat* OR map* OR methodologic* OR quantitative OR evidence OR literature)) OR AB ((review N3 (effectiveness OR effects OR systemat* OR synth* OR integrat* OR map* OR methodologic* OR quantitative OR evidence OR literature)) OR SU ((review N3 (effectiveness OR effects OR systemat* OR synth* OR integrat* OR map* OR methodologic* OR quantitative OR evidence OR literature))) OR (TI(("meta regression" OR "meta synth*" OR "meta‐synth*" OR "meta analy*" OR "metaanaly*" OR "meta‐analy*" OR "metanaly*" OR "metaregression" OR "meta‐regression" OR "methodologic* overview" OR "pool* analys*" OR "pool* data" OR "quantitative* overview" OR "research integration")) OR AB ("meta regression" OR "meta synth*" OR "meta‐synth*" OR "meta analy*" OR "metaanaly*" OR "meta‐analy*" OR "metanaly*" OR "metaregression" OR "metaregression" OR "methodologic* overview" OR "pool* analys*" OR "pool* data" OR "quantitative* overview" OR "research integration") OR SU ("meta regression" OR "meta synth*" OR "meta‐synth*" OR "meta analy*" OR "metaanaly*" OR "meta‐analy*" OR "metanaly*" OR "metaregression" OR "meta‐regression" OR "methodologic* overview" OR "pool* analys*" OR "pool* data" OR "quantitative* overview" OR "research integration")) OR (TI((systematic* OR synthes*) N3 (research OR evaluation* OR finding* OR thematic* OR report OR descriptive OR explanatory OR narrative OR meta* OR review* OR data OR literature OR studies OR evidence OR map OR quantitative OR study OR studies OR paper OR impact OR impacts OR effect* OR compar*)) OR AB((systematic* OR synthes*) N3 (research OR evaluation* OR finding* OR thematic* OR report OR descriptive OR explanatory OR narrative OR meta* OR review* OR data OR literature OR studies OR evidence OR map OR quantitative OR study OR studies OR paper OR impact OR impacts OR effect* OR compar*)) OR SU((systematic* OR synthes*) N3 (research OR evaluation* OR finding* OR thematic* OR report OR descriptive OR explanatory OR narrative OR meta* OR review* OR data OR literature OR studies OR evidence OR map OR quantitative OR study OR studies OR paper OR impact OR impacts OR effect* OR compar*)))	Limiters ‐ Year of Publication: 2010‐2021 Expanders ‐ Apply equivalent subjects Search modes ‐ Boolean/Phrase	Interface ‐ EBSCOhost Research Databases Search Screen ‐ Advanced Search Database ‐ APA PsycArticles;APA PsycInfo	115,458
S2	(TI(women OR "women's right" OR "young women" OR woman* OR adolescent* OR girl* OR schoolgirl* OR "school girl*" OR female* OR man OR men OR "young men" OR boy* OR schoolboy* OR "School boy*" OR teen* OR youth* OR young* OR mother* OR father* OR parent* OR caregiver) OR AB (women OR "women's right" OR "young women" OR woman* OR adolescent* OR girl* OR schoolgirl* OR “school girl*” OR female* OR man OR men OR "young men" OR boy* OR schoolboy* OR "school boy*" OR teen* OR youth* OR young* OR mother* OR father* OR parent* OR caregiver OR SU (women OR "women's right" OR "young women" OR woman* OR adolescent* OR Girl* OR schoolgirl* OR “school girl*” OR female* OR man OR men OR "young men" OR boy* OR schoolboy* OR "school boy*" OR teen* OR youth* OR young* OR mother* OR father* OR parent* OR caregiver)) OR (TI(femini* OR masculi* OR female*OR male* OR gender* OR "social equality" OR "social equity" OR empower* OR "equal opportunit*" OR egalitarian*) OR AB(femini* OR masculi* OR female*OR male* OR gender* OR "social equality" OR "social equity" OR empower* OR "equal opportunit*" OR egalitarian*) OR SU(femini* OR masculi* OR female*OR male* OR gender* OR "social equality" OR "social equity" OR empower* OR "equal opportunit*" OR egalitarian*))	Limiters ‐ Year of Publication: 2010‐2021 Expanders ‐ Apply equivalent subjects Search modes ‐ Boolean/Phrase	Interface ‐ EBSCOhost Research Databases Search Screen ‐ Advanced Search Database ‐ APA PsycArticles;APA PsycInfo	925,928
S1	(TI(afghanistan OR albania OR algeria OR "american samoa" OR angola OR antigua OR barbuda OR argentina OR armenia OR armenian OR aruba OR azerbaijan OR bahrain OR bangladesh OR barbados OR belarus OR byelarus OR belorussia OR byelorussian OR belize OR "british honduras" OR benin OR dahomey OR Bhutan OR bolivia OR bosnia OR herzegovina OR botswana OR bechuanaland OR brazil OR brasil OR bulgaria OR "burkina faso" OR "burkina fasso" OR "upper volta" OR burundi OR urundi OR "cabo verde" OR "cape verde" OR cambodia OR kampuchea OR "khmer republic" OR cameroon OR cameron OR cameroun OR "central african republic" OR "ubangi shari" OR chad OR chile OR china OR colombia OR comoros OR "comoro islands" OR mayotte OR congo OR zaire OR "costa rica" OR "cote d'ivoire" OR "cote d'ivoire" OR "cote d'ivoire" OR "ivory coast" OR croatia OR cuba OR cyprus OR "czech republic" OR czechoslovakia OR djibouti OR "french somaliland" OR dominica OR "dominican republic" OR ecuador OR egypt OR "united arab republic" OR "el salvador" OR "equatorial guinea" OR "spanish guinea" OR eritrea OR Estonia OR eswatini OR swaziland OR ethiopia OR fiji OR gabon OR "gabonese republic" OR gambia OR georgia OR georgian OR ghana OR "gold coast" OR gibraltar OR greece OR grenada OR guam OR guatemala OR guinea OR guyana OR guiana OR haiti OR hispaniola OR honduras OR hungary OR india OR indonesia OR timor OR iran OR iraq OR "isle of man" OR jamaica OR jordan OR kazakhstan OR kazakh OR kenya OR korea OR kosovo OR kyrgyzstan OR kirghizia OR kirgizstan OR "kyrgyz republic" OR kirghiz OR laos OR "lao pdr" OR "lao people's democratic republic" OR latvia OR lebanon OR lesotho OR basutoland OR liberia OR libya OR "libyan arab OR jamahiriya" OR lithuania OR macau OR macao OR macedonia OR madagascar OR "malagasy republic" OR malawi OR nyasaland OR malaysia OR maldives OR "indian ocean" OR mali OR malta OR micronesia OR kiribati OR "marshall islands" OR nauru OR "northern mariana islands" OR palau OR tuvalu OR mauritania OR mauritius OR mexico OR moldova OR moldovian OR mongolia OR montenegro OR morocco OR ifni OR mozambique OR "portuguese east africa" OR myanmar OR burma OR namibia OR nepal OR "netherlands antilles" OR nicaragua OR niger OR nigeria OR oman OR muscat OR pakistan OR panama OR "papua new guinea" OR paraguay OR peru OR philippines OR philipines OR phillipines OR phillippines OR poland OR "polish people's republic" OR portugal OR "portuguese republic" OR "puerto rico" OR romania OR russia OR "russian federation" OR ussr OR "soviet union" OR "union of soviet socialist republics" OR rwanda OR ruanda OR samoa OR "pacific islands" OR polynesia OR "samoan islands" OR "sao tome" and principe OR "saudi arabia" OR senegal OR serbia OR seychelles OR "sierra leone" OR slovakia OR "slovak republic" OR slovenia OR melanesia OR "solomon island" OR "solomon islands" OR "norfolk island" OR somalia OR "south africa" OR "south sudan" OR "sri lanka" OR ceylon OR "saint kitts" OR nevis OR "st kitts" OR nevis OR "saint lucia" OR "st lucia" OR "saint vincent" OR "st vincent" OR grenadines OR sudan OR suriname OR surinam OR syria OR "syrian arab republic" OR tajikistan OR tadjikistan OR tadzhikistan OR tadzhik OR tanzania OR tanganyika OR thailand OR siam OR "timor leste" OR "east timor" OR togo OR "togolese republic" OR tonga OR trinidad OR tobago OR tunisia OR turkey OR turkmenistan OR turkmen OR uganda OR ukraine OR uruguay OR uzbekistan OR uzbek OR vanuatu OR "new hebrides" OR venezuela OR vietnam OR "vietname" OR "west bank" OR gaza OR palestine OR yemen OR yugoslavia OR zambia OR zimbabwe OR "northern rhodesia")) OR (AB(afghanistan OR albania OR algeria OR "american samoa" OR angola OR antigua OR barbuda OR argentina OR armenia OR armenian OR aruba OR azerbaijan OR bahrain OR bangladesh OR barbados OR belarus OR byelarus OR belorussia OR byelorussian OR belize OR "british honduras" OR benin OR dahomey OR bhutan OR bolivia OR bosnia OR herzegovina OR botswana OR bechuanaland OR brazil OR brasil OR bulgaria OR "burkina faso" OR "burkina fasso" OR "upper volta" OR burundi OR urundi OR "cabo verde" OR "cape verde" OR cambodia OR kampuchea OR "khmer republic" OR cameroon OR cameron OR cameroun OR "central african republic" OR "ubangi shari" OR chad OR chile OR china OR colombia OR comoros OR "comoro islands" OR mayotte OR congo OR zaire OR "costa rica" OR "cote d'ivoire" OR "cote d'ivoire" OR "cote d'ivoire" OR "ivory coast" OR croatia OR cuba OR cyprus OR "czech republic" OR czechoslovakia OR djibouti OR "french somaliland" OR dominica OR "dominican republic" OR ecuador OR egypt OR "united arab republic" OR "el salvador" OR "equatorial guinea" OR "spanish guinea" OR eritrea OR Estonia OR eswatini OR swaziland OR ethiopia OR fiji OR gabon OR "gabonese republic" OR gambia OR georgia OR georgian OR ghana OR "gold coast" OR gibraltar OR greece OR grenada OR guam OR guatemala OR guinea OR guyana OR guiana OR haiti OR hispaniola OR honduras OR hungary OR india OR indonesia OR timor OR iran OR iraq OR "isle of man" OR jamaica OR jordan OR kazakhstan OR kazakh OR kenya OR korea OR kosovo OR kyrgyzstan OR kirghizia OR kirgizstan OR "kyrgyz republic" OR kirghiz OR laos OR "lao pdr" OR "lao people's democratic republic" OR latvia OR lebanon OR lesotho OR basutoland OR liberia OR libya OR "libyan arab OR jamahiriya" OR lithuania OR macau OR macao OR macedonia OR madagascar OR "malagasy republic" OR malawi OR nyasaland OR malaysia OR maldives OR "indian ocean" OR mali OR malta OR micronesia OR kiribati OR "marshall islands" OR nauru OR "northern mariana islands" OR palau OR tuvalu OR mauritania OR mauritius OR mexico OR moldova OR moldovian OR mongolia OR montenegro OR morocco OR ifni OR mozambique OR "portuguese east africa" OR myanmar OR burma OR namibia OR nepal OR "netherlands antilles" OR nicaragua OR niger OR nigeria OR oman OR muscat OR pakistan OR panama OR "papua new guinea" OR paraguay OR peru OR philippines OR philipines OR phillipines OR phillippines OR poland OR "polish people's republic" OR portugal OR "portuguese republic" OR "puerto rico" OR romania OR russia OR "russian federation" OR ussr OR "soviet union" OR "union of soviet socialist republics" OR rwanda OR ruanda OR samoa OR "pacific islands" OR polynesia OR "samoan islands" OR "sao tome" and principe OR "saudi arabia" OR senegal OR serbia OR seychelles OR "sierra leone" OR slovakia OR "slovak republic" OR slovenia OR melanesia OR "solomon island" OR "solomon islands" OR "norfolk island" OR somalia OR "south africa" OR "south sudan" OR "sri lanka" OR ceylon OR "saint kitts" OR nevis OR "st kitts" OR nevis OR "saint lucia" OR "st lucia" OR "saint vincent" OR "st vincent" OR grenadines OR sudan OR suriname OR surinam OR syria OR "syrian arab republic" OR tajikistan OR tadjikistan OR tadzhikistan OR tadzhik OR tanzania OR Tanganyika OR thailand OR siam OR "timor leste" OR "east timor" OR togo OR "togolese republic" OR tonga OR trinidad OR tobago OR tunisia OR turkey OR turkmenistan OR turkmen OR uganda OR ukraine OR uruguay OR uzbekistan OR uzbek OR vanuatu OR "new hebrides" OR venezuela OR vietnam OR "vietname" OR "west bank" OR gaza OR palestine OR yemen OR yugoslavia OR zambia OR zimbabwe OR "northern rhodesia")) OR (SU(afghanistan OR albania OR algeria OR "american samoa" OR angola OR antigua OR barbuda OR argentina OR armenia OR armenian OR aruba OR azerbaijan OR bahrain OR bangladesh OR barbados OR belarus OR byelarus OR belorussia OR byelorussian OR belize OR "british honduras" OR benin OR dahomey OR bhutan OR bolivia OR bosnia OR herzegovina OR botswana OR bechuanaland OR brazil OR brasil OR bulgaria OR "burkina faso" OR "burkina fasso" OR "upper volta" OR burundi OR urundi OR "cabo verde" OR "cape verde" OR cambodia OR kampuchea OR "khmer republic" OR cameroon OR cameron OR cameroun OR "central african republic" OR "ubangi shari" OR chad OR chile OR china OR colombia OR comoros OR "comoro islands" OR mayotte OR congo OR zaire OR "costa rica" OR "cote d'ivoire" OR "cote d'ivoire" OR "cote d'ivoire" OR "ivory coast" OR croatia OR cuba OR cyprus OR "czech republic" OR czechoslovakia OR djibouti OR "french somaliland" OR dominica OR "dominican republic" OR ecuador OR egypt OR "united arab republic" OR "el salvador" OR "equatorial guinea" OR "spanish guinea" OR eritrea OR Estonia OR eswatini OR swaziland OR ethiopia OR fiji OR gabon OR "gabonese republic" OR gambia OR georgia OR georgian OR ghana OR "gold coast" OR gibraltar OR greece OR grenada OR guam OR guatemala OR guinea OR guyana OR guiana OR haiti OR hispaniola OR honduras OR hungary OR india OR indonesia OR timor OR iran OR iraq OR "isle of man" OR jamaica OR jordan OR kazakhstan OR kazakh OR kenya OR korea OR kosovo OR kyrgyzstan OR kirghizia OR kirgizstan OR "kyrgyz republic" OR kirghiz OR laos OR "lao pdr" OR "lao people's democratic republic" OR latvia OR lebanon OR lesotho OR basutoland OR liberia OR libya OR "libyan arab OR jamahiriya" OR lithuania OR macau OR macao OR macedonia OR madagascar OR "malagasy republic" OR malawi OR nyasaland OR malaysia OR maldives OR "indian ocean" OR mali OR malta OR micronesia OR kiribati OR "marshall islands" OR nauru OR "northern mariana islands" OR palau OR tuvalu OR mauritania OR mauritius OR mexico OR moldova OR moldovian OR mongolia OR montenegro OR morocco OR ifni OR mozambique OR "portuguese east africa" OR myanmar OR burma OR namibia OR nepal OR "netherlands antilles" OR nicaragua OR niger OR nigeria OR oman OR muscat OR pakistan OR panama OR "papua new guinea" OR paraguay OR peru OR philippines OR philipines OR phillipines OR phillippines OR poland OR "polish people's republic" OR portugal OR "portuguese republic" OR "puerto rico" OR romania OR russia OR "russian federation" OR ussr OR "soviet union" OR "union of soviet socialist republics" OR rwanda OR ruanda OR samoa OR "pacific islands" OR polynesia OR "samoan islands" OR "sao tome" and principe OR "saudi arabia" OR senegal OR serbia OR seychelles OR "sierra leone" OR slovakia OR "slovak republic" OR slovenia OR melanesia OR "solomon island" OR "solomon islands" OR "norfolk island" OR somalia OR "south africa" OR "south sudan" OR "sri lanka" OR ceylon OR "saint kitts" OR nevis OR "st kitts" OR nevis OR "saint lucia" OR "st lucia" OR "saint vincent" OR "st vincent" OR grenadines OR sudan OR suriname OR surinam OR syria OR "syrian arab republic" OR tajikistan OR tadjikistan OR tadzhikistan OR tadzhik OR tanzania OR tanganyika OR thailand OR siam OR "timor leste" OR "east timor" OR togo OR "togolese republic" OR tonga OR trinidad OR tobago OR tunisia OR turkey OR turkmenistan OR turkmen OR uganda OR ukraine OR uruguay OR uzbekistan OR uzbek OR vanuatu OR "new hebrides" OR venezuela OR vietnam OR "vietname" OR "west bank" OR gaza OR palestine OR yemen OR yugoslavia OR zambia OR zimbabwe OR "northern rhodesia")) OR (TI("west indies" OR "indian ocean islands" OR caribbean OR "central america" OR "latin america" OR "south america" OR magreb OR maghrib OR "central asia" OR "north asia" OR "northern asia" OR "southeastern asia" OR "south eastern asia" OR "southeast asia" OR "south east asia" OR "western asia" OR "east europe" OR "eastern europe") OR AB("west indies" OR "indian ocean islands" OR caribbean OR "central america" OR "latin america" OR "south america" OR magreb OR maghrib OR "central asia" OR "north asia" OR "northern asia" OR "southeastern asia" OR "south eastern asia" OR "southeast asia" OR "south east asia" OR "western asia" OR "east europe" OR "eastern europe") OR SU("west indies" OR "indian ocean islands" OR caribbean OR "central america" OR "latin america" OR "south america" OR magreb OR maghrib OR "central asia" OR "north asia" OR "northern asia" OR "southeastern asia" OR "south eastern asia" OR "southeast asia" OR "south east asia" OR "western asia" OR "east europe" OR "eastern europe")) OR (TI(africa OR asia OR caribbean OR "west indies" OR "south america" OR "latin america" OR "central america" OR "middle east" OR "global south") OR AB(africa OR asia OR caribbean OR "west indies" OR "south america" OR "latin america" OR "central america" OR "middle east" OR "global south") OR SU(africa OR asia OR caribbean OR "west indies" OR "south america" OR "latin america" OR "central america" OR "middle east" OR "global south")) OR (TI((developing OR (less* N1 developed) OR "under developed" OR underdeveloped OR "under served" OR underserved OR deprived OR poor* OR "middle income" OR (low* N1 income)) N1 (countr* OR nation* OR population* OR world)) OR AB((developing OR (less* N1 developed) OR "under developed" OR underdeveloped OR "under served" OR underserved OR deprived OR poor* OR "middle income" OR (low* N1 income)) N1 (countr* OR nation* or population* OR world)) OR SU((developing OR (less* N1 developed) OR "under developed" OR underdeveloped OR "under served" OR underserved OR deprived OR poor* OR "middle income" OR (low* N1 income)) N1 (countr* OR nation* or population* OR world))) OR (TI((developing OR (less* N1 developed) OR "under developed" OR “middle income" OR (low* N1 income)) N1 (economy OR economies)) OR AB(developing OR (less* N1 developed) OR "under developed" OR "middle income" OR (low* N1 income)) N1 (economy OR economies)) OR SU((developing OR (less* N1 developed) OR "under developed" OR "middle income" OR (low* N1 income) N1 (economy OR economies))) OR (TI(low N3 middle N3 countr*) OR AB(low N3 middle N3 countr*) OR SU(low N3 middle N3 countr*)) OR (TI(low* N1 (gdp OR gnp OR "gross domestic" OR "gross national")) OR AB(low* N1 (gdp OR gnp OR "gross domestic" OR "gross national")) OR SU(low* N1 (gdp OR gnp OR "gross domestic" OR "gross national"))) OR (TI(lmic OR lmics OR "third world" OR "lami country" OR "lami countries") OR AB(lmic OR lmics OR "third world" OR "lami country" OR "lami countries") OR SU(lmic OR lmics OR "third world" OR "lami country" OR "lami countries")) OR (TI("transitional country" OR "transitional countries") OR AB("transitional country" OR "transitional countries") OR SU("transitional country" OR "transitional countries")) OR (TI(emerging N1 (economy or economies OR nation*) OR AB(emerging N1 (economy OR economies OR nation*) OR SU(emerging N1 (economy OR economies OR nation*))	Limiters ‐ Year of Publication: 2010‐2021 Expanders ‐ Apply equivalent subjects Search modes ‐ Boolean/Phrase	Interface ‐ EBSCOhost Research Databases Search Screen ‐ Advanced Search Database ‐ APA PsycArticles;APA PsycInfo	167,147

## APPENDIX H WEB OF SCIENCE

Database name: Web of Science

Date of search: 01/03/2021

Number of search results: 3860

**Table:**

# 16	3860	#15 AND #7 AND #6 AND #5 Indexes = SCI‐EXPANDED, SSCI, A&HCI, CPCI‐S, CPCI‐SSH, ESCI, CCR‐EXPANDED, IC Timespan = 2010‐2021
# 15	15,872,095	#14 OR #13 OR #12 OR #11 OR #10 OR #9 OR #8 *Indexes = SCI‐EXPANDED, SSCI, A&HCI, CPCI‐S, CPCI‐SSH, ESCI, CCR‐EXPANDED, IC Timespan = 2010‐2021*
# 14	13,240,007	CU = (afghanistan OR albania OR algeria OR "american samoa" OR angola OR antigua OR barbuda OR argentina OR armenia OR armenian OR aruba OR azerbaijan OR bahrain OR bangladesh OR barbados OR belarus OR byelarus OR belorussia OR byelorussian OR belize OR "british honduras" OR benin OR dahomey OR bhutan OR bolivia OR bosnia OR herzegovina OR Botswana OR bechuanaland OR brazil OR brasil OR bulgaria OR "burkina faso" OR "burkina fasso" OR "upper volta" OR burundi OR urundi OR "cabo verde" OR "cape verde" OR cambodia OR kampuchea OR "khmer republic" OR cameroon OR cameron OR cameroun OR "central african republic" OR "ubangi shari" OR chad OR chile OR china OR colombia OR comoros OR "comoro islands" OR mayotte OR congo OR zaire OR "costa rica" OR "cote d'ivoire" OR "cote d'ivoire" OR "cote d'ivoire" OR "ivory coast" OR croatia OR cuba OR cyprus OR "czech republic" OR czechoslovakia OR djibouti OR "french somaliland" OR dominica OR "dominican republic" OR ecuador OR egypt OR "united arab republic" OR "el salvador" OR "equatorial guinea" OR "spanish guinea" OR eritrea OR estonia OR eswatini OR swaziland OR ethiopia OR fiji OR gabon OR "gabonese republic" OR gambia OR georgia OR Georgian OR ghana OR "gold coast" OR gibraltar OR greece OR grenada OR guam OR guatemala OR guinea OR guyana OR guiana OR haiti OR hispaniola OR honduras OR hungary OR india OR indonesia OR timor OR iran OR iraq OR "isle of man" OR jamaica OR jordan OR kazakhstan OR kazakh OR kenya OR korea OR kosovo OR kyrgyzstan OR kirghizia OR kirgizstan OR "kyrgyz republic" OR kirghiz OR laos OR "lao pdr" OR "lao people's democratic republic" OR latvia OR lebanon OR lesotho OR basutoland OR liberia OR libya OR "libyan arab jamahiriya" OR lithuania OR macau OR macao OR macedonia OR madagascar OR "malagasy republic" OR malawi OR nyasaland OR malaysia OR maldives OR "indian ocean" OR mali OR malta OR micronesia OR kiribati OR "marshall islands" OR nauru OR "northern mariana islands" OR palau OR tuvalu OR mauritania OR mauritius OR mexico OR moldova OR moldovian OR mongolia OR montenegro OR morocco OR ifni OR mozambique OR "portuguese east africa" OR myanmar OR burma OR namibia OR nepal OR "netherlands antilles" OR nicaragua OR niger OR nigeria OR oman OR muscat OR pakistan OR panama OR "papua new guinea" OR paraguay OR peru OR philippines OR philipines OR phillipines OR phillippines OR poland OR "polish people's republic" OR portugal OR "portuguese republic" OR "puerto rico" OR romania OR russia OR "russian federation" OR ussr OR "soviet union" OR "union of soviet socialist republics" OR rwanda OR ruanda OR samoa OR "pacific islands" OR polynesia OR "samoan islands" OR "sao tome" and principe OR "saudi arabia" OR senegal OR serbia OR seychelles OR "sierra leone" OR slovakia OR "slovak republic" OR slovenia OR melanesia OR "solomon island" OR "solomon islands" OR "norfolk island" OR somalia OR "south africa" OR "south sudan" OR "sri lanka" OR ceylon OR "saint kitts" OR nevis OR "st kitts" OR nevis OR "saint lucia" OR "st lucia" OR "saint vincent" OR "st vincent" OR grenadines OR sudan OR suriname OR surinam OR syria OR "syrian OR muscat OR pakistan OR panama OR "papua new guinea" OR paraguay OR peru OR philippines OR philipines OR phillipines OR phillippines OR poland OR "polish people's republic" OR portugal OR "portuguese republic" OR "puerto rico" OR romania OR russia OR "russian federation" OR ussr OR "soviet union" OR "union of soviet socialist republics" OR rwanda OR ruanda OR samoa OR "pacific islands" OR polynesia OR "samoan islands" OR "sao tome" and principe OR "saudi arabia" OR senegal OR serbia OR seychelles OR "sierra leone" OR slovakia OR "slovak republic" OR slovenia OR melanesia OR "solomon island" OR "solomon islands" OR "norfolk island" OR somalia OR "south africa" OR "south sudan" OR "sri lanka" OR ceylon OR "saint kitts" OR nevis OR "st kitts" OR nevis OR "saint lucia" OR "st lucia" OR "saint vincent" OR "st vincent" OR grenadines OR sudan OR suriname OR surinam OR syria OR "syrian arab republic" OR tajikistan OR tadjikistan OR tadzhikistan OR tadzhik OR tanzania OR Tanganyika OR thailand OR siam OR "timor leste" OR "east timor" OR togo OR "togolese republic" OR tonga OR trinidad OR tobago OR tunisia OR turkey OR turkmenistan OR turkmen OR uganda OR ukraine OR uruguay OR uzbekistan OR uzbek OR vanuatu OR "new hebrides" OR venezuela OR vietnam OR "viet name" OR "west bank" OR gaza OR palestine OR yemen OR yugoslavia OR zambia OR zimbabwe OR "northern rhodesia") OR SU (afghanistan OR albania OR algeria OR "american samoa" OR angola OR antigua OR barbuda OR argentina OR armenia OR armenian OR aruba OR azerbaijan OR bahrain OR bangladesh OR barbados OR belarus OR byelarus OR belorussia OR byelorussian OR belize OR "british honduras" OR benin OR dahomey OR bhutan OR bolivia OR bosnia OR herzegovina OR Botswana OR bechuanaland OR brazil OR brasil OR bulgaria OR "burkina faso" OR "burkina fasso" OR "upper volta" OR burundi OR urundi OR "cabo verde" OR "cape verde" OR cambodia OR kampuchea OR "khmer republic" OR cameroon OR cameron OR cameroun OR "central african republic" OR "ubangi shari" OR chad OR chile OR china OR colombia OR comoros OR "comoro islands" OR mayotte OR congo OR zaire OR "costa rica" OR "cote d'ivoire" OR "cote d'ivoire" OR "cote d'ivoire" OR "ivory coast" OR croatia OR cuba OR cyprus OR "czech republic" OR czechoslovakia OR djibouti OR "french somaliland" OR dominica OR "dominican republic" OR ecuador OR egypt OR "united arab republic" OR "el salvador" OR "equatorial guinea" OR "spanish guinea" OR eritrea OR estonia OR eswatini OR swaziland OR ethiopia OR fiji OR gabon OR "gabonese republic" OR gambia OR georgia OR Georgian OR ghana OR "gold coast" OR gibraltar OR greece OR grenada OR guam OR guatemala OR guinea OR guyana OR guiana OR haiti OR hispaniola OR honduras OR hungary OR india OR indonesia OR timor OR iran OR iraq OR "isle of man" OR jamaica OR jordan OR kazakhstan OR kazakh OR kenya OR korea OR kosovo OR kyrgyzstan OR kirghizia OR kirgizstan OR "kyrgyz republic" OR kirghiz OR laos OR "lao pdr" OR "lao people's democratic republic" OR latvia OR lebanon OR lesotho OR basutoland OR liberia OR libya OR "libyan arab jamahiriya" OR lithuania OR macau OR macao OR macedonia OR madagascar OR "malagasy republic" OR malawi OR nyasaland OR malaysia OR maldives OR "indian ocean" OR mali OR malta OR micronesia OR kiribati OR "marshall islands" OR nauru OR "northern mariana islands" OR palau OR tuvalu OR mauritania OR mauritius OR mexico OR moldova OR moldovian OR mongolia OR montenegro OR morocco OR ifni OR mozambique OR "portuguese east africa" OR myanmar OR burma OR namibia OR nepal OR "netherlands antilles" OR nicaragua OR niger OR nigeria OR oman OR muscat OR pakistan OR panama OR "papua new guinea" OR paraguay OR peru OR philippines OR philipines OR phillipines OR phillippines OR poland OR "polish people's republic" OR portugal OR "portuguese republic" OR "puerto rico" OR romania OR russia OR "russian federation" OR ussr OR "soviet union" OR "union of soviet socialist republics" OR rwanda OR ruanda OR samoa OR "pacific islands" OR polynesia OR "samoan islands" OR "sao tome" and principe OR "saudi arabia" OR senegal OR serbia OR seychelles OR "sierra leone" OR slovakia OR "slovak republic" OR slovenia OR melanesia OR "solomon island" OR "solomon islands" OR "norfolk island" OR somalia OR "south africa" OR "south sudan" OR "sri lanka" OR ceylon OR "saint kitts" OR nevis OR "st kitts" OR nevis OR "saint lucia" OR "st lucia" OR "saint vincent" OR "st vincent" OR grenadines OR sudan OR suriname OR surinam OR syria OR "syrian OR muscat OR pakistan OR panama OR "papua new guinea" OR paraguay OR peru OR philippines OR philipines OR phillipines OR phillippines OR poland OR "polish people's republic" OR portugal OR "portuguese republic" OR "puerto rico" OR romania OR russia OR "russian federation" OR ussr OR "soviet union" OR "union of soviet socialist republics" OR rwanda OR ruanda OR samoa OR "pacific islands" OR polynesia OR "samoan islands" OR "sao tome" and principe OR "saudi arabia" OR senegal OR serbia OR seychelles OR "sierra leone" OR slovakia OR "slovak republic" OR slovenia OR melanesia OR "solomon island" OR "solomon islands" OR "norfolk island" OR somalia OR "south africa" OR "south sudan" OR "sri lanka" OR ceylon OR "saint kitts" OR nevis OR "st kitts" OR nevis OR "saint lucia" OR "st lucia" OR "saint vincent" OR "st vincent" OR grenadines OR sudan OR suriname OR surinam OR syria OR "syrian arab republic" OR tajikistan OR tadjikistan OR tadzhikistan OR tadzhik OR tanzania OR Tanganyika OR thailand OR siam OR "timor leste" OR "east timor" OR togo OR "togolese republic" OR tonga OR trinidad OR tobago OR tunisia OR turkey OR turkmenistan OR turkmen OR uganda OR ukraine OR uruguay OR uzbekistan OR uzbek OR vanuatu OR "new hebrides" OR venezuela OR vietnam OR "viet name" OR "west bank" OR gaza OR palestine OR yemen OR yugoslavia OR zambia OR zimbabwe OR "northern rhodesia") *Indexes = SCI‐EXPANDED, SSCI, A&HCI, CPCI‐S, CPCI‐SSH, ESCI, CCR‐EXPANDED, IC Timespan = 2010‐2021*
# 13	2,495,095	TS = (afghanistan OR albania OR algeria OR "american samoa" OR angola OR antigua OR barbuda OR argentina OR armenia OR armenian OR aruba OR azerbaijan OR bahrain OR bangladesh OR barbados OR belarus OR byelarus OR belorussia OR byelorussian OR belize OR "british honduras" OR benin OR dahomey OR bhutan OR bolivia OR bosnia OR herzegovina OR Botswana OR bechuanaland OR brazil OR brasil OR bulgaria OR "burkina faso" OR "burkina fasso" OR "upper volta" OR burundi OR urundi OR "cabo verde" OR "cape verde" OR cambodia OR kampuchea OR "khmer republic" OR cameroon OR cameron OR cameroun OR "central african republic" OR "ubangi shari" OR chad OR chile OR china OR colombia OR comoros OR "comoro islands" OR mayotte OR congo OR zaire OR "costa rica" OR "cote d'ivoire" OR "cote d'ivoire" OR "cote d'ivoire" OR "ivory coast" OR croatia OR cuba OR cyprus OR "czech republic" OR czechoslovakia OR djibouti OR "french somaliland" OR dominica OR "dominican republic" OR ecuador OR egypt OR "united arab republic" OR "el salvador" OR "equatorial guinea" OR "spanish guinea" OR eritrea OR estonia OR eswatini OR swaziland OR ethiopia OR fiji OR gabon OR "gabonese republic" OR gambia OR georgia OR Georgian OR ghana OR "gold coast" OR gibraltar OR greece OR grenada OR guam OR guatemala OR guinea OR guyana OR guiana OR haiti OR hispaniola OR honduras OR hungary OR india OR indonesia OR timor OR iran OR iraq OR "isle of man" OR jamaica OR jordan OR kazakhstan OR kazakh OR kenya OR korea OR kosovo OR kyrgyzstan OR kirghizia OR kirgizstan OR "kyrgyz republic" OR kirghiz OR laos OR "lao pdr" OR "lao people's democratic republic" OR latvia OR lebanon OR lesotho OR basutoland OR liberia OR libya OR "libyan arab jamahiriya" OR lithuania OR macau OR macao OR macedonia OR madagascar OR "malagasy republic" OR malawi OR nyasaland OR malaysia OR maldives OR "indian ocean" OR mali OR malta OR micronesia OR kiribati OR "marshall islands" OR nauru OR "northern mariana islands" OR palau OR tuvalu OR mauritania OR mauritius OR mexico OR moldova OR moldovian OR mongolia OR montenegro OR morocco OR ifni OR mozambique OR "portuguese east africa" OR myanmar OR burma OR namibia OR nepal OR "netherlands antilles" OR nicaragua OR niger OR nigeria OR oman OR muscat OR pakistan OR panama OR "papua new guinea" OR paraguay OR peru OR philippines OR philipines OR phillipines OR phillippines OR poland OR "polish people's republic" OR portugal OR "portuguese republic" OR "puerto rico" OR romania OR russia OR "russian federation" OR ussr OR "soviet union" OR "union of soviet socialist republics" OR rwanda OR ruanda OR samoa OR "pacific islands" OR polynesia OR "samoan islands" OR "sao tome" and principe OR "saudi arabia" OR senegal OR serbia OR seychelles OR "sierra leone" OR slovakia OR "slovak republic" OR slovenia OR melanesia OR "solomon island" OR "solomon islands" OR "norfolk island" OR somalia OR "south africa" OR "south sudan" OR "sri lanka" OR ceylon OR "saint kitts" OR nevis OR "st kitts" OR nevis OR "saint lucia" OR "st lucia" OR "saint vincent" OR "st vincent" OR grenadines OR sudan OR suriname OR surinam OR syria OR "syrian OR muscat OR pakistan OR panama OR "papua new guinea" OR paraguay OR peru OR philippines OR philipines OR phillipines OR phillippines OR poland OR "polish people's republic" OR portugal OR "portuguese republic" OR "puerto rico" OR romania OR russia OR "russian federation" OR ussr OR "soviet union" OR "union of soviet socialist republics" OR rwanda OR ruanda OR samoa OR "pacific islands" OR polynesia OR "samoan islands" OR "sao tome" and principe OR "saudi arabia" OR senegal OR serbia OR seychelles OR "sierra leone" OR slovakia OR "slovak republic" OR slovenia OR melanesia OR "solomon island" OR "solomon islands" OR "norfolk island" OR somalia OR "south africa" OR "south sudan" OR "sri lanka" OR ceylon OR "saint kitts" OR nevis OR "st kitts" OR nevis OR "saint lucia" OR "st lucia" OR "saint vincent" OR "st vincent" OR grenadines OR sudan OR suriname OR surinam OR syria OR "syrian arab republic" OR tajikistan OR tadjikistan OR tadzhikistan OR tadzhik OR tanzania OR Tanganyika OR thailand OR siam OR "timor leste" OR "east timor" OR togo OR "togolese republic" OR tonga OR trinidad OR tobago OR tunisia OR turkey OR turkmenistan OR turkmen OR uganda OR ukraine OR uruguay OR uzbekistan OR uzbek OR vanuatu OR "new hebrides" OR venezuela OR vietnam OR "viet name" OR "west bank" OR gaza OR palestine OR yemen OR yugoslavia OR zambia OR zimbabwe OR "northern rhodesia") OR SU (afghanistan OR albania OR algeria OR "american samoa" OR angola OR antigua OR barbuda OR argentina OR armenia OR armenian OR aruba OR azerbaijan OR bahrain OR bangladesh OR barbados OR belarus OR byelarus OR belorussia OR byelorussian OR belize OR "british honduras" OR benin OR dahomey OR bhutan OR bolivia OR bosnia OR herzegovina OR Botswana OR bechuanaland OR brazil OR brasil OR bulgaria OR "burkina faso" OR "burkina fasso" OR "upper volta" OR burundi OR urundi OR "cabo verde" OR "cape verde" OR cambodia OR kampuchea OR "khmer republic" OR cameroon OR cameron OR cameroun OR "central african republic" OR "ubangi shari" OR chad OR chile OR china OR colombia OR comoros OR "comoro islands" OR mayotte OR congo OR zaire OR "costa rica" OR "cote d'ivoire" OR "cote d'ivoire" OR "cote d'ivoire" OR "ivory coast" OR croatia OR cuba OR cyprus OR "czech republic" OR czechoslovakia OR djibouti OR "french somaliland" OR dominica OR "dominican republic" OR ecuador OR egypt OR "united arab republic" OR "el salvador" OR "equatorial guinea" OR "spanish guinea" OR eritrea OR estonia OR eswatini OR swaziland OR ethiopia OR fiji OR gabon OR "gabonese republic" OR gambia OR georgia OR Georgian OR ghana OR "gold coast" OR gibraltar OR greece OR grenada OR guam OR guatemala OR guinea OR guyana OR guiana OR haiti OR hispaniola OR honduras OR hungary OR india OR indonesia OR timor OR iran OR iraq OR "isle of man" OR jamaica OR jordan OR kazakhstan OR kazakh OR kenya OR korea OR kosovo OR kyrgyzstan OR kirghizia OR kirgizstan OR "kyrgyz republic" OR kirghiz OR laos OR "lao pdr" OR "lao people's democratic republic" OR latvia OR lebanon OR lesotho OR basutoland OR liberia OR libya OR "libyan arab jamahiriya" OR lithuania OR macau OR macao OR macedonia OR madagascar OR "malagasy republic" OR malawi OR nyasaland OR malaysia OR maldives OR "indian ocean" OR mali OR malta OR micronesia OR kiribati OR "marshall islands" OR nauru OR "northern mariana islands" OR palau OR tuvalu OR mauritania OR mauritius OR mexico OR moldova OR moldovian OR mongolia OR montenegro OR morocco OR ifni OR mozambique OR "portuguese east africa" OR myanmar OR burma OR namibia OR nepal OR "netherlands antilles" OR nicaragua OR niger OR nigeria OR oman OR muscat OR pakistan OR panama OR "papua new guinea" OR paraguay OR peru OR philippines OR philipines OR phillipines OR phillippines OR poland OR "polish people's republic" OR portugal OR "portuguese republic" OR "puerto rico" OR romania OR russia OR "russian federation" OR ussr OR "soviet union" OR "union of soviet socialist republics" OR rwanda OR ruanda OR samoa OR "pacific islands" OR polynesia OR "samoan islands" OR "sao tome" and principe OR "saudi arabia" OR senegal OR serbia OR seychelles OR "sierra leone" OR slovakia OR "slovak republic" OR slovenia OR melanesia OR "solomon island" OR "solomon islands" OR "norfolk island" OR somalia OR "south africa" OR "south sudan" OR "sri lanka" OR ceylon OR "saint kitts" OR nevis OR "st kitts" OR nevis OR "saint lucia" OR "st lucia" OR "saint vincent" OR "st vincent" OR grenadines OR sudan OR suriname OR surinam OR syria OR "syrian OR muscat OR pakistan OR panama OR "papua new guinea" OR paraguay OR peru OR philippines OR philipines OR phillipines OR phillippines OR poland OR "polish people's republic" OR portugal OR "portuguese republic" OR "puerto rico" OR romania OR russia OR "russian federation" OR ussr OR "soviet union" OR "union of soviet socialist republics" OR rwanda OR ruanda OR samoa OR "pacific islands" OR polynesia OR "samoan islands" OR "sao tome" and principe OR "saudi arabia" OR senegal OR serbia OR seychelles OR "sierra leone" OR slovakia OR "slovak republic" OR slovenia OR melanesia OR "solomon island" OR "solomon islands" OR "norfolk island" OR somalia OR "south africa" OR "south sudan" OR "sri lanka" OR ceylon OR "saint kitts" OR nevis OR "st kitts" OR nevis OR "saint lucia" OR "st lucia" OR "saint vincent" OR "st vincent" OR grenadines OR sudan OR suriname OR surinam OR syria OR "syrian arab republic" OR tajikistan OR tadjikistan OR tadzhikistan OR tadzhik OR tanzania OR Tanganyika OR thailand OR siam OR "timor leste" OR "east timor" OR togo OR "togolese republic" OR tonga OR trinidad OR tobago OR tunisia OR turkey OR turkmenistan OR turkmen OR uganda OR ukraine OR uruguay OR uzbekistan OR uzbek OR vanuatu OR "new hebrides" OR venezuela OR vietnam OR "viet name" OR "west bank" OR gaza OR palestine OR yemen OR yugoslavia OR zambia OR zimbabwe OR "northern rhodesia")*Indexes = SCI‐EXPANDED, SSCI, A&HCI, CPCI‐S, CPCI‐SSH, ESCI, CCR‐EXPANDED, IC Timespan = 2010‐2021*
# 12	20,442	TS = ("transitional country" OR "transitional countries") OR TS = (emerging NEAR (economy OR economies OR nation*)) *Indexes = SCI‐EXPANDED, SSCI, A&HCI, CPCI‐S, CPCI‐SSH, ESCI, CCR‐EXPANDED, IC Timespan = 2010‐2021*
# 11	26,354	TS = (low NEAR/3 middle NEAR/3 countr*) OR TS = (low* NEAR (gdp OR gnp OR "gross domestic" OR "gross national")) OR TS = (lmic OR lmics OR "third world" OR "lami country" OR "lami countries") *Indexes = SCI‐EXPANDED, SSCI, A&HCI, CPCI‐S, CPCI‐SSH, ESCI, CCR‐EXPANDED, IC Timespan = 2010‐2021*
# 10	21,084	TS = ((developing OR (less* NEAR developed) OR "under developed" OR "middle income" OR (low* NEAR income)) NEAR (economy OR economies)) *Indexes = SCI‐EXPANDED, SSCI, A&HCI, CPCI‐S, CPCI‐SSH, ESCI, CCR‐EXPANDED, IC Timespan = 2010‐2021*
# 9	4,063,953	TS = (((developing OR (less* NEAR developed) OR "under developed" OR underdeveloped OR "under served" OR underserved OR deprived OR poor* OR "middle income") OR (low* NEAR income) NEAR (countr* OR nation* OR population* OR world))) *Indexes = SCI‐EXPANDED, SSCI, A&HCI, CPCI‐S, CPCI‐SSH, ESCI, CCR‐EXPANDED, IC Timespan = 2010‐2021*
# 8	438,341	TS = ("west indies" OR "indian ocean islands" OR caribbean OR "central america" OR "latin america" OR "south america" OR magreb OR maghrib OR "central asia" OR "north asia" OR "northern asia" OR "southeastern asia" OR "south eastern asia" OR "southeast asia" OR "south east asia" OR "western asia" OR "east europe" OR "eastern europe") OR TS = (africa OR asia OR caribbean OR "west indies" OR "south america" OR "latin america" OR "central america" OR "middle east" OR "global south") *Indexes = SCI‐EXPANDED, SSCI, A&HCI, CPCI‐S, CPCI‐SSH, ESCI, CCR‐EXPANDED, IC Timespan = 2010‐2021*
# 7	3,387,879	TS = (women OR "women's right" OR "young women" OR woman* OR adolescent* OR girl* OR schoolgirl* OR "school girl*" OR female* OR man OR men OR "young men" OR boy* OR schoolboy* OR "school boy*" OR teen* OR youth* OR young* OR mother* OR father* OR parent* OR caregiver) OR TS = (femini* OR masculi* OR female* OR male* OR gender* OR "social equality" OR "social equity" OR empower* OR "equal opportunit*" OR egalitarian*) *Indexes = SCI‐EXPANDED, SSCI, A&HCI, CPCI‐S, CPCI‐SSH, ESCI, CCR‐EXPANDED, IC Timespan = 2010‐2021*
# 6	1,554,196	TS = (review NEAR(effectiveness OR effects OR systemat* OR synth* OR integrat* OR map* OR methodologic* OR quantitative OR evidence OR literature)) OR TS = ("meta regression" OR "meta synth*" OR "meta‐synth*" OR "meta analy*" OR “metaanaly*” OR "meta‐analy*" OR “metanaly*” OR "metaregression" OR "meta‐regression" OR "methodologic* overview" OR "pool* analys*" OR "pool* data" OR "quantitative* overview" OR "research integration") OR TS = ((systematic* OR synthes*) NEAR (research OR evaluation* OR finding* OR thematic* OR report OR descriptive OR explanatory OR narrative OR meta* OR review* OR data OR literature OR evidence OR map OR quantitative OR study OR studies OR paper OR impact OR impacts OR effect* OR compar* OR comprehensive OR overview)) *Indexes = SCI‐EXPANDED, SSCI, A&HCI, CPCI‐S, CPCI‐SSH, ESCI, CCR‐EXPANDED, IC Timespan = 2010‐2021*
# 5	445,056	#4 OR #3 OR #2 OR #1 *Indexes = SCI‐EXPANDED, SSCI, A&HCI, CPCI‐S, CPCI‐SSH, ESCI, CCR‐EXPANDED, IC Timespan = 2010‐2021*
# 4	188,356	TS = (((family OR birth OR income) NEAR/1(allowance OR maintenance OR grant OR payment)) OR (leave NEAR/1(parental OR paternity OR maternity OR sick)) OR (welfare NEAR/1(infant OR maternal)) OR (support NEAR/1(disability OR nutritional OR compensatory)) OR (voucher NEAR/1(food OR school OR education)) OR (assistance NEAR/1(humanitarian)) OR (service* NEAR/2("ancillary school" OR homemaker OR "school health" OR "child care*" OR community OR "home care" OR "adult protective")) OR(aid NEAR/4 ("to families with dependent children" OR "to the totally disabled" OR "to visually impaired")) OR (community* NEAR/1(care OR intervention OR integration OR project)) OR (care NEAR/2(day OR "for children" OR "for elderly" OR "foster home" OR "centre‐based child" OR "center‐based child")) OR (education NEAR/1(parenting OR vocational)) OR (tax NEAR/1(exemption OR break OR credit*)) OR ("after school club" OR "after school program*" OR family support)) *Indexes = SCI‐EXPANDED, SSCI, A&HCI, CPCI‐S, CPCI‐SSH, ESCI, CCR‐EXPANDED, IC Timespan = 2010‐2021*
# 3	40,235	TS = (("economic asset" OR "fee waiver*" OR "school feeding" OR "disability grant*" OR "food stamp" OR "sick pay" OR medicaid OR medicare OR "home health nursing" OR "nurse family partnership") OR (intervention* NEAR/3("intimate partner violence" OR "domestic violence" OR "domestic abuse" OR ipv OR "family violence" OR "partner violence" OR "spous* abuse" OR "battered women" OR abusive))) *Indexes = SCI‐EXPANDED, SSCI, A&HCI, CPCI‐S, CPCI‐SSH, ESCI, CCR‐EXPANDED, IC Timespan = 2010‐2021*
# 2	159,017	TS = ((labo$r market NEAR/1(policy OR policies OR program* OR reform * OR standard OR supply)) OR (employment NEAR/1(scheme OR support OR service)) OR (training NEAR/1 (vocational OR job OR support OR employee)) OR (subsid* NEAR/1(wage OR hiring OR health)) OR ("activation policies" OR "skills development" OR "skill development" or "entrepreneurship promotion" or "public works") OR (insurance NEAR/2 (health OR household OR community‐based OR national OR unemployment OR micro‐health OR "micro health")) OR (child NEAR/2(support OR welfare OR grant OR care OR "care cash")) OR (assistance NEAR/2 (medical OR employee OR nutrition OR food OR public OR social OR "old age")) OR (benefit* NEAR/1(paternity OR maternity OR system OR retirement OR unemployment OR fringe OR employee OR health OR child OR death OR system OR insurance)) OR ((wage OR housing) NEAR/1(subsidy OR subsidies OR public OR elderly))) *Indexes = SCI‐EXPANDED, SSCI, A&HCI, CPCI‐S, CPCI‐SSH, ESCI, CCR‐EXPANDED, IC Timespan = 2010‐2021*
# 1	102,539	TS = (((social NEAR/1(care* OR protection OR insurance OR policy OR policies OR inclusion OR security or assistance or service* OR welfare OR grant OR pension* OR "safety net"))) OR (program* NEAR/2 ("cash plus" OR lunch OR breakfast OR "school health" OR "food security" OR "poverty alleviation" OR "poverty reduction" OR livelihood* OR "employee assistance" OR employment OR empowerment OR entrepreneurship OR enterpreneurship OR employment OR "employee assistance" OR "youth employment" OR integrated OR community‐based)) OR (transfer NEAR/1(resource OR in‐kind OR conditional OR unconditional OR financial OR monetary)) OR (pension NEAR/1(retirement OR public OR "old age")) OR (financial NEAR/1(incentive OR protection))) *Indexes = SCI‐EXPANDED, SSCI, A&HCI, CPCI‐S, CPCI‐SSH, ESCI, CCR‐EXPANDED, IC Timespan = 2010‐2021*

## APPENDIX I SCREENING FRAMEWORK

**Table:**

Screening phase	Criteria	Note
Screening on title and abstract	Exclude if not systematic review	Is the study a systematic review? A systematic review summarizes the best available evidence on a specific question using transparent procedures to locate, evaluate, and integrate the findings of relevant research—see review protocol for additional criteria Alternative terms include systematic literature review, rapid review, rapid evidence assessment, meta‐analysis or evidence synthesis.
	Exclude on date	Was the review published before 01/01/2010?
	Exclude if not social protection intervention	Does the review investigate social protection programmes or a programme sub‐type? Table 1 of systematic review protocol contains full list of the four categories (social assistance, social insurance, labour market programmes, social care services), definitions and examples. Loans, credits, life insurance, savings and financial training alone are not considered social protection.
	Exclude if only includes studies from high‐income countries	Is the review only focused on studies conducted in countries classified as High Income Countries? Countries classified as LMICs and included in the review https://epoc.cochrane.org/lmic-filters
	Exclude if interventions from only private organisations	Does the review only investigate social protection programmes implemented by private organisations? Public‐private partnerships are included but solely private efforts are not.
	Exclude if retracted	Was the review retracted?
	Include for second opinion	Unsure
	Include on title and abstract	Include
Screening on full‐text	Exclude if not systematic review	Is the study a systematic review? A systematic review summarizes the best available evidence on a specific question using transparent procedures to locate, evaluate, and integrate the findings of relevant research – see review protocol for additional criteria Alternative terms include systematic literature review, rapid review, rapid evidence assessment, meta‐analysis or evidence synthesis.
	Exclude on date	Was the review published before 01/01/2010?
	Exclude if not social protection intervention	Does the review investigate social protection programmes or a programme sub‐type? Table 1 of systematic review protocol contains full list of the four categories (social assistance, social insurance, labour market programmes, social care services), definitions and examples. Loans, credits, life insurance, savings and financial training alone are not considered social protection.
	Exclude if only includes studies from high‐income countries	Is the review only focused on studies conducted in countries classified as High Income Countries? Countries classified as LMICs and included in the review https://epoc.cochrane.org/lmic-filters
	Exclude if not disaggregated by country	Do reviews including studies from countries of various income levels disaggregate results by country?
	Exclude if interventions from only private organisations	Does the review only investigate social protection programmes implemented by private organisations? Public‐private partnerships are included but solely private efforts are not.
	Exclude if retracted	Was the review retracted?
	Exclude on lack of gender disaggregation	Does the review provide results disaggregated by gender?
	Include for another opinion	Unsure
	Include on full‐text	Include
